# Ras/Raf/MEK/ERK and PI3K/PTEN/Akt/mTOR Cascade Inhibitors: How Mutations Can Result in Therapy Resistance and How to Overcome Resistance

**DOI:** 10.18632/oncotarget.659

**Published:** 2012-10-20

**Authors:** James A. McCubrey, Linda S. Steelman, William H. Chappell, Stephen L. Abrams, Richard A. Franklin, Giuseppe Montalto, Melchiorre Cervello, Massimo Libra, Saverio Candido, Grazia Malaponte, Maria C. Mazzarino, Paolo Fagone, Ferdinando Nicoletti, Jörg Bäsecke, Sanja Mijatovic, Danijela Maksimovic-Ivanic, Michele Milella, Agostino Tafuri, Francesca Chiarini, Camilla Evangelisti, Lucio Cocco, Alberto M. Martelli

**Affiliations:** ^1^ Department of Microbiology and Immunology, Brody School of Medicine at East Carolina University, Greenville, NC, USA; ^2^ Department of Internal Medicine and Specialties, University of Palermo, Palermo, Italy; ^3^ Consiglio Nazionale delle Ricerche, Istituto di Biomedicina e Immunologia Molecolare “Alberto Monroy”, Palermo, Italy; ^4^ Department of Bio-Medical Sciences, University of Catania, Catania, Italy; ^5^ Department of Medicine, University of Göttingen, Göttingen, Germany; ^6^ Department of Immunology, Instititue for Biological Research “Sinisa Stankovic”, University of Belgrade, Belgrade, Serbia; ^7^ Regina Elena National Cancer Institute, Rome, Italy; ^8^ Sapienza, University of Rome, Department of Cellular Biotechnology and Hematology, Rome, Italy; ^9^ Institute of Molecular Genetics, National Research Council-Rizzoli Orthopedic Institute, Bologna, Italy; ^10^ Department of Biomedical and Neuromotor Sciences, University of Bologna, Bologna, Italy

**Keywords:** Targeted Therapy, Therapy Resistance, Cancer Stem Cells, Raf, Akt, PI3K, mTOR

## Abstract

The Ras/Raf/MEK/ERK and PI3K/PTEN/Akt/mTOR cascades are often activated by genetic alterations in upstream signaling molecules such as receptor tyrosine kinases (RTK). Targeting these pathways is often complex and can result in pathway activation depending on the presence of upstream mutations (e.g., Raf inhibitors induce Raf activation in cells with wild type (WT) *RAF* in the presence of mutant, activated *RAS*) and rapamycin can induce Akt activation. Targeting with inhibitors directed at two constituents of the same pathway or two different signaling pathways may be a more effective approach. This review will first evaluate potential uses of Raf, MEK, PI3K, Akt and mTOR inhibitors that have been investigated in pre-clinical and clinical investigations and then discuss how cancers can become insensitive to various inhibitors and potential strategies to overcome this resistance.

## INTRODUCTION

### Predicting Sensitivity to Small Molecule Inhibitors

Recent studies have examined extensive panels of cell lines for mutations of genes implicated in cancer as well as for their sensitivity to various inhibitors and chemotherapeutic drugs commonly used to treat cancers [1,2]. The cell lines were examined by expression profiling, chromosome copy number, deep sequencing, biostatistical and systems analyses. Both studies indicated that sensitivity to inhibitors was often associated with genetic mutations at key elements in the Ras/Raf/MEK/ERK, PI3K/PTEN/Akt/mTOR and some other pathways. One study has generated a “Cancer Cell Line Encyclopedia” which will be useful for predictive modeling of inhibitor sensitivity [1]. Sensitivity to MEK and Raf inhibitors was often investigated in these studies. Sensitivity to the B-Raf inhibitor PLX4720 was shown to be highly associated with particular mutations at *BRAF* (V600E). Sensitivity to MEK inhibitors was shown to be associated with *BRAF*, *NRAS* as well as *PTEN*, *PTPN5*, *SPRY2*, *DUSP4*, *DUSP6* mutations and to a lesser extent mutations at *KRAS*. Sensitivity to MEK inhibitors in *NRAS* mutant lines was associated with aryl hydrocarbon receptor (AHR) expression [2].

### Overview of Pathway Inhibitors

Effective inhibitors specific for many of the key components of the Ras/Raf/MEK/ERK and Ras/PI3K/PTEN/mTOR pathways have been developed [3-11]. In many cases, these inhibitors have been examined in clinical trials. Furthermore, inhibitors that target the mutant protein more than the wild type (WT) protein of various genes (*e.g*., *BRAF* and *PIK3CA*) either have been or are being characterized. Thus specific inhibitors have been made and some are currently used in the clinic. Targeting some components of these pathways has proven clinically effective. In some of the diseases, there are a very large number of patients with few effective treatments [(*e.g*., Sorafenib and hepatocellular carcinoma (HCC)] [11-13].

### Raf/MEK Inhibitors

Raf inhibitors have been developed and some are being used for therapy while others are being evaluated in clinical trials. Raf inhibitors have in general exhibited greater response rates in clinical trails than MEK inhibitors which may be related to the broader therapeutic index of Raf inhibitors that suppress ERK activity in a mutant-allele specific fashion as opposed to MEK inhibitors which suppress MEK activity in tumor and normal cells [14]. Some inhibitors (*i.e,* Sorafenib, Bayer) were initially thought to specifically inhibit Raf but have been subsequently shown to have multiple targets (*e.g*., VEGF-R, Flt-3, c-Kit, PDGF-R) [15-17]. However, that does not preclude their usefulness in cancer therapy. Sorafenib is approved for the treatment of certain cancers (*e.g.,* renal cell carcinoma (RCC) and patients with unresectable HCC). Sorafenib was evaluated in the Sorafenib Hepatocellular carcinoma Assessment Randomized Protocol (SHARP) trial, which demonstrated that the drug was effective in prolonging median survival and time-to-progression in patients with advanced HCC [11,12]. Sorafenib is generally well tolerated in HCC patients with a manageable adverse events profile [11,12]. The effects of sorafenib in combination with other drugs have been evaluated in HCC [16].

**Figure 1:**
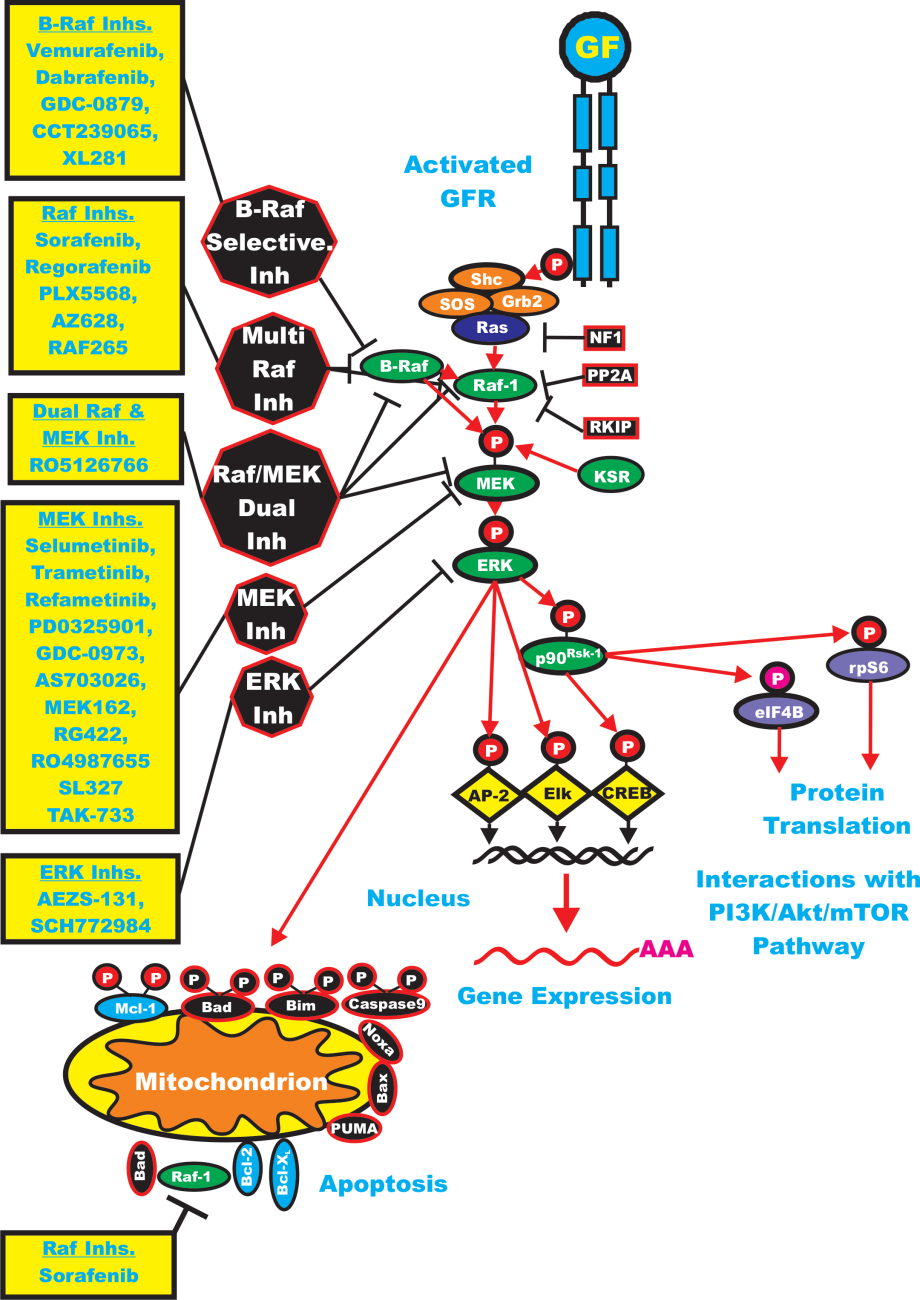
Overview of the Ras/Raf/MEK/ERK Cascade and Small Molecule Inhibitors Used for Targeting this Pathway Activation of this pathway can occur by mutations in upstream growth factor receptors (GFR) or by stimulation by the appropriate growth factors (GF). In addition, mutations can occur in intrinsic members of the pathway (*RAS RAF*, *MEK1* or the tumor suppressor Neurofibromin (*NF1*). GFR and GR are indicated in blue. Kinases are indicated in green ovals. Coupling molecules are indicated by orange ovals. The Ras molecule is indicated by a purple oval. Transcription factors are indicated by yellow diamonds. Sites where NF1, protein phosphatase 2A (PP2A) Raf kinase inhibitory protein (RKIP), kinase suppressor of Ras (KSR) interact with this pathway are on the right hand side of the Ras/Raf/MEK/ERK pathway. NF1, PP2A and RKIP are depicted in black rectangles as they normally serve to dampen the activity of this pathway. Molecules such as Mcl-1 which are anti-apoptotic and phosphorylated by ERK and Akt are indicated by blue ovals, other anti-apoptotic molecule are also indicated by blue ovals. Pro-apoptotic molecules are indicated by black ovals. Red arrows indicate activating events in pathways. Sites where various small molecule inhibitors function are in black octagons on the left hand side of the pathway. Representative inhibitors are listed in yellow boxes next to the octagons. Red arrows indicate activating events in pathways. Black arrows indicating inactivating events in pathway. Activating phosphorylation events are depicted in red circles with Ps with a black outlined circle. Inactivating phosphorylation events are depicted in black circles with Ps with a red outlined circle.

While sorafenib is not considered effective for the treatment of most melanomas with *BRAF* V600E mutations, it may be effective in the treatment of a minority of melanomas with G469E and D594G mutations which express constitutive ERK1/2 but low levels of MEK. These melanomas are sensitive to sorafenib, potentially because they signal through Raf-1 [18]. MEK inhibitors have also been examined for treating HCC in mouse models [13] but they do not appear to be as effective as Sorafenib, most likely due to the broad specificity of Sorafenib, which inhibits other targets besides Raf. An overview of where these inhibitors function is presented in Figure 1.

PLX-4032 (a.k.a., Zelborab, vemurafenib, Plexxikon/Roche) is a B-Raf inhibitor that has and is being evaluated in many clinical trials [19-22]. Vemurafenib has been approved by the US Food and Drug Administration (FDA) for the treatment of patients with unresectable or metastatic melanoma carrying the *BRAF* (V600E) mutation. For vemurafenib to be clinically effective, it needs to suppress downstream ERK activation essentially completely [22]. Vemurafenib is in phase II clinical trials (NCT0128653) for patients with metastatic or unresectable papillary thyroid cancer (PTC) which have the *BRAF* V600E mutation and are also resistant to radioactive iodine therapy. NCT01524978 is a phase I clinical trial to evaluate the effects of Vemurafenib on patients with multiple myeloma and other cancers containing the *BRAF* V600E mutation.

PLX-4720 (Plexxikon/Roche) (R7204) is a mutant B-Raf specific inhibitor that was used for preclinical studies [23]. Our accompanying manuscript published in *Oncotarget* discusses the mutations of various components of these pathways as well as their biochemical functions [24]. PLX-4720 was designed using a unique screening platform developed by Plexxikon that involved the use of structural and medicinal chemistry techniques [25]. This more selective screening approach has resulted in a series of B-Raf inhibitors based on the structural implications of *BRAF* mutation and which discriminate between the mutant and WT protein. PLX-4720 is orally available and is highly selective for the mutant B-Raf protein. PLX-4720 is effective against melanomas, as well as colorectal cancer (CRC) and other cancers, with the *BRAF* V600E mutation. *BRAF* V600E has been associated with more aggressive tumors and lower rates of patient survival [25]. The IC_50_ value for PLX-4720 is approximately 3-fold lower in *in vitro* kinase assays with mutant versus WT B-Raf proteins and demonstrates an approximately 60-fold lower IC_50_ value *in vivo* when cell lines with mutant and WT *BRAF* genes are compared [25]. The IC_50_ value for PLX-4720 was compared with sorafenib in a panel of melanomas, CRC and non small cell lung cancer (NSCLC). The *BRAF* gene status was known in all of these cell lines. The IC_50_ value for PXL-4720 was approximately 100-fold lower (range: 17.5 to 280 nM) than sorafenib in melanomas and colon carcinomas that had the *BRAF* V600E mutation; however, the IC_50_ value for PLX-4720 was approximately the same as sorafenib in colon carcinomas and NSCLC without *BRAF* mutations, but with *RAS* mutations. PLX-4720 arrests mutant but not WT *BRAF* melanoma cells at the G_0_/G_1_ cell-cycle stage and initiates apoptosis in these cells.

Studies examining the effects of sorafenib on sorafenib-resistant cell lines transfected with *BRAF* genes containing gatekeeper mutations indicated that the mutant B-Raf signaling was resistant to sorafenib, but sorafenib still inhibited tumor growth driven by the mutant B-Raf protein. In essence sorafenib was inhibiting Raf-1 activity which was induced by the mutant B-Raf protein. In contrast, PLX-4720 inhibited tumor growth by targeting oncogenic B-Raf. These studies indicated that sorafenib suppressed tumor growth independently of B-Raf while PLX-4720 directly inhibited the oncogenic effects of B-Raf [26].

GSK2118436 (a.k.a. dabrafenib) is an ATP-competitive inhibitor of mutant B-Raf, WT B-Raf and WT Raf-1 developed by GlaxoSmithKlein (GSK) in clinic trial (NCT00880321), which examined patients with melanoma, brain metastases, in other solid tumours it was determined to be safe and elicited responses. It was an active inhibitor of *BRAF* V600E in this trial [27].

CCT239065 is a mutant B-Raf inhibitor developed at the Institute of Cancer Research in London, UK [28]. It inhibits *BRAF* mutant allele (V600E) signaling and proliferation more than WT *BRAF*-mediated signaling. Its effects are more selective for cells containing mutant *BRAF* than WT *BRAF*. CCT239065 is well tolerated in mice and had good oral bioavailability. It suppressed tumors containing *BRAF* mutant genes but not WT *BRAF* tumors in mice tumor xenograft studies [28].

GDC-0879 is a *BRAF* mutant allele selective inhibitor developed by Genentech which has been evaluated in pre-clinical studies [29]. The efficacy GDC-0879 is related to the *BRAF* V600E mutational status in the cancer cells and inhibition of downstream MEK and ERK activity.

PLX5568 is a selective Raf kinase inhibitor developed by Plexicon. It is being examined for the treatment of polycystic kidney disease (PKD). In the kidney, Raf-1 is localized to the tubular cells where it is linked to many physiologically important functions. PLX5568 suppressed cyst enlargement in a rat model of PKD but did not improve kidney function as fibrosis was not suppressed [30].

Raf-265 is an ATP-competitive pan-Raf inhibitor developed by Novartis. Treatment of bronchus carcinoid NCI-H727 and insulinoma cells (CM insulinoma cell line) with Raf-265 enhanced sensitivity to TRAIL-induced apoptosis. These cells are normally resistant to PI3K/mTOR inhibitors when combined with TRAIL. Raf-265 was shown to decrease Bcl-2 levels which correlated with their sensitivity to TRAIL-mediated apoptosis. This approach may be effective in the therapy of neuroendocrine tumors [31]. Raf-265 is being evaluated in a clinical trial (NCT00304525) for treatment of patients with locally advanced or metastatic melanoma.

Regorafenib (BAY 73-4506) is an oral multikinase inhibitor of angiogenic, stromal and oncogenic RTKs developed by Bayer. Regorafenib inhibits RTKs such as VEGF-R2, VEGF-R1/3, PDGF-Rβ, fibroblast growth factor receptor-1 as well as mutant Kit, RET and B-Raf. The effects of regorafenib on tumor growth have been evaluated in human xenograft models in mice, and tumor shrinkages were observed in breast MDA-MB-231 and renal 786-O carcinoma models [32].

AZ628 is a selective Raf inhibitor developed by Astra Zenica. *BRAF*-mutant melanoma cells are normally very sensitive to AZ628. However, when AZ628 cells are grown for prolonged periods of time, they become resistant to AZ628 by upregulating the expression of Raf-1 [33].

XL281 is an orally-active WT and mutant RAF kinases selective inhibitor developed by Exelixis and Bristol-Myers Squibb. It has been examined in clinical trials primarily with patients having *BRAF* mutations (CRC, melanoma, PTC and NSCLC) [34].

### Results of Clinical Trials with Sorafenib

Some of first clinical trials with Raf inhibitors were with sorafenib (Nexavar^™^) in metastatic RCC [35]. Clinical trials with melanoma were also done around the same time period [36]. The clinical trials with melanoma patients and sorafenib as a single agent did not yield encouraging results. Due to the broad specificity of sorafenib this drug has been evaluated for the therapy of diverse cancers, including RCC, melanoma and HCC (due to the involvement of the Raf/MEK/ERK cascade, as well as altered VEGFR pathway in these cancers) and gastro-intestinal stromal tumors (GIST) (due to the involvement of *KIT* mutations in this cancer). Sorafenib has been approved for the treatment of renal cancer, including RCC in 2005 and for HCC in 2007. Although *BRAF* is not mutated in RCC, VEGFR-2 may be aberrantly expressed as there is dysregulation of its cognate ligand VEGF which can activate VEGFR2 and the Raf/MEK/ERK cascade. Sorafenib is active as a single agent in RCC, probably due to its ability to suppress the activities of essential growth-required signaling pathways.

Phase II and larger phase III clinical trails with sorafenib combined with chemotherapy or targeted therapy were performed. NCT00461851 was a phase II trial with bladder cancer patients. It combined sorafenib with gemcitabine and carboplatin. NCT01371981 was a phase II/III with sorafenib and the proteosomal inhibitor bortezomib as well as various chemotherapeutic drugs including asparaginease, cytarabine, daunorubicin and mitoxantrone in patients with acute myeloid leukemia (AML) and yielded variable results with low response rates [38].

### Effects of Sorafenib on Melanomas

As the *BRAF* gene is mutated in approximately 50 to 70% of melanomas, sorafenib was evaluated for its ability to suppress melanoma growth in mouse models [39]. Most *BRAF* mutations occur at V600E. Sorafenib had only modest activity as a single agent in advanced melanoma and it did not appear to be more effective in the treatment of melanomas that are either WT or mutant at the *BRAF* gene, hence it may be targeting a kinase other than B-Raf in these melanomas (*e.g*., VEGFR or Raf-1). Alternatively, it could be targeting an upstream receptor kinase which signals through the Ras/Raf/MEK/ERK cascade. It is relevant to examine the effects of combining sorafenib with a MEK inhibitor to treat malignant melanoma and certain other cancers. Sorafenib may target the VEGFR and other membrane receptors expressed on the particular cancer cells, whereas the MEK inhibitor would specifically suppress the Raf/MEK/ERK cascade which is abnormally activated by the *BRAF* oncogene or other mutant upstream signaling molecules. To improve the effectiveness of sorafenib in the therapy of melanoma, it is being combined with standard chemotherapeutic drugs.

### Results of Clinical Trials with Vemurafenib

Phase I, II and III clinical trials with vemurafenib have been performed. A greater than 90% reduction in active ERK was necessary for clinical response [22]. In the phase III clinical trial comparing vemurafenib with the standard of care chemotherapeutic drug decarbazine, the trial was terminated prematurely as it was apparent that vemurafenib was more effective than decarbazine [40]. Vemurafenib was approved for the treatment of unresectable metastatic *BRAF* mutant melanoma in 2011. Recently, the results of a phase II clinical trial (NCT00949702) indicated that vemurafenib induces clinical responses in greater than 50% of previously treated mutant *BRAF* (V600E or V600K) melanoma patients the median overall survival was approximately 16 months [41].

### Results of Clinical Trials with Dabrafenib (GSK2118436)

Dabrafenib has also displayed positive results in Phase I/II trials [42,43]. Dabrafenib is in ongoing Phase II clinical trials (NCT01153763) as a single agent in patients with *BRAF* mutant melanoma.

### Need for Genetic Screening Before Treatment with Raf Kinase Inhibitors

It is critical to determine the genetic status at both *BRAF* and *RAS* before treatment with Raf inhibitors [44-46]. Class I B-Raf inhibitors (active conformation inhibitors) such as (vemurafenib and dabrafenib) will inhibit *BRAF* mutants, however these ATP-competitive B-Raf inhibitors will not inhibit WT B-Raf in the presence of activated Ras expression. In fact, these B-Raf inhibitors can activate Raf-1 in these cells in the presence of active Ras. The Raf inhibitors can induce B-Raf binding to Raf-1. Vemurafenib can, to a lesser extent, induce B-Raf binding to Raf-1 when the ERK-mediated negative feedback loop on B-Raf was inhibited with a MEK inhibitor. These binding events were determined to require the presence of activated Ras (WT or mutant), which may be necessary for the translocation from the cytoplasm to the membrane and assembly into the signaling complex. This has therapeutic implications, as after treatment of patients with mutant *RAS* with certain B-Raf inhibitors, B-Raf can bind and activate Raf-1 and promote the oncogenic pathway. In fact, even kinase-dead *BRAF* mutations, which have been observed in human cancer [47], the mutant B-Raf proteins can dimerize with Raf-1, when stimulated by the mutant Ras protein and activate the Raf/MEK/ERK cascade. For Raf-selective inhibitors to be therapeutically useful, prior screening of patients for *RAS* mutations will be necessary, as well as perhaps additional screening during treatment. Otherwise resistance may develop and lead to further stimulation of the Raf/MEK/ERK cascade.

ATP-competitive Raf inhibitors inhibit ERK signaling in cells with mutant *BRAF*, but enhance signaling in cells with WT *BRAF*. Drug-mediated transactivation of Raf dimers was shown to be responsible for the activation of the enzyme by inhibitors. The Raf inhibitors bind to the ATP-binding site of the Raf dimer. The inhibitors can also bind to B-Raf:Raf-1 heterodimers. Raf activity is dependent on Ras activity. The Raf inhibitor binding to one Raf protomer results in the inhibition of that protomer, but activation of the remaining protomer. *RAS* is not normally mutated in cells with *BRAF* mutants and there is minimal Ras activity. Hence in *BRAF-*mutant cells, Raf inhibitors will be effective in inhibiting downstream MEK:ERK signaling. However in cells with active Ras, they will not [44,45]. These basic science observations have been essentially confirmed in clinical trials [19,20,22].

Raf activation occurs after treatment of certain cancer patients with Raf inhibitors. This abnormal Raf activation can lead to skin diseases such as keratoacanthomas (KAs) and cutaneous squamous cell carcinomas (cSCCs) in patients with *RAS* mutations. These results indicate that co-targeting with Raf and MEK inhibitors may be appropriate in patients who have active Raf and B-Raf [48,49].

### Resistance to Raf Inhibitors

A problem with treatment of melanoma patients with mutant *BRAF* is the emergence of inhibitor-resistance which occurs frequently and relatively rapidly after treatment with the Raf inhibitors (2-18 months) [50]. This may be due to the persistence of melanoma cancer initiating cells (CICs) [51-54]. Some of these CICs may have other mutations besides *BRAF*.

There are many different mechanisms by which melanoma cells can become resistant to Raf inhibitors [55]. Unlike resistance mechanisms observed in some other cancers such as imatinib-resistant chronic myeloid leukemia (CML) where the resistant cells often have mutations in the gatekeeper residues in *BCRABL* which allows the cells to proliferate and activate additional signaling pathways in the presence of imatinib, others mechanism for Raf inhibitor-resistance are more frequently observed in cells containing *BRAF* mutants. Gatekeeper mutations in *BRAF* can be created experimentally, and the cells are resistant to the B-Raf specific inhibitors, but these mutations do not appear to occur frequently in B-Raf inhibitor*-*resistant clinical specimens [50,59,60].

Poulikakos and colleagues demonstrated a novel resistance mechanism which involves a splice variant in the mutated *BRAF* allele that leads to a loss of the Ras binding domain in the B-Raf protein that prevents dimerization. This mutant form of *BRAF* V600E elicits enhanced dimerization in cells which contain low levels of active Ras, in comparison to cells containing the full-length *BRAF* V600E mutation. The truncated B-Raf V600E kinase can dimerize with Raf-1 and induce downstream MEK/ERK in the absence of activating Ras mutations and the cells are resistant to the Raf inhibitors [61]. This splicing mutation was determined to be present in *BRAF V600E* in six of nineteen vemurafenib-treated patient samples which had undergone relapse.

**Figure 2:**
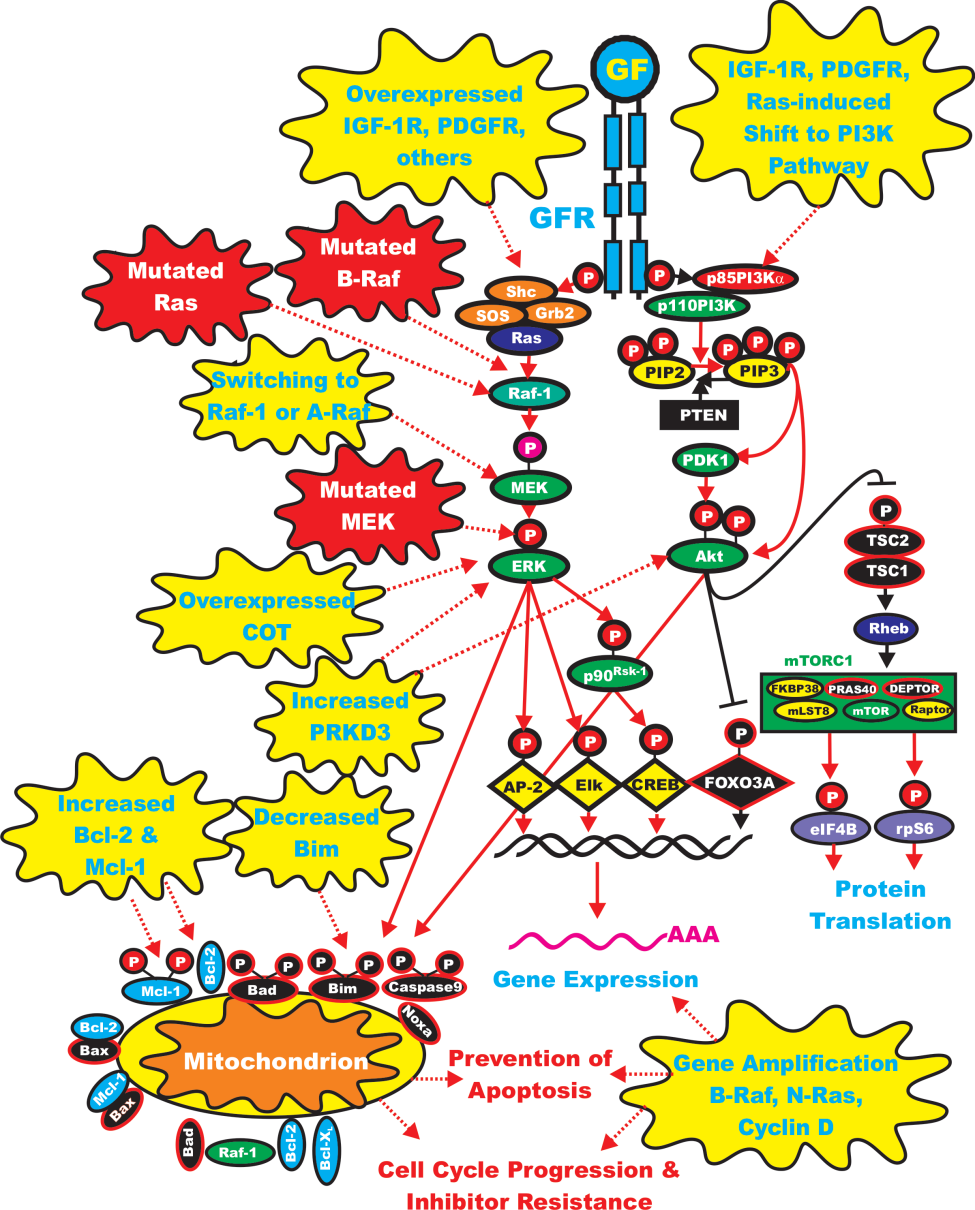
Sites of Mutations which can Result in Resistance to Raf and MEK Inhibitors Sites of mutation which result in sensitivity to Raf and MEK inhibitors are indicated in red irregular circles. The same color scheme present in Figure 1 for other signaling molecules is continuted in this figure. Signaling induced by mutations is indicated by red dashed lines. Secondary mutation/events which result in inhibitor resistance are depicted in yellow irregular circle. Signaling induced by mutations is indicated by red dashed lines.

Many different types of gene deregulation events have been observed in B-Raf inhibitor-resistant cells [62,63]. Mutations at cyclin-dependent kinase 4 (CDK4) and amplification of cyclin-D1 have been documented in clinical specimens from B-Raf inhibitor-treated patients which underwent remission [64]. A diagram illustrating some of the mechanisms by which cells become resistant to Raf and MEK inhibitors is presented in Figure 2.

Amplification of the B-Raf gene has been reported in some B-Raf inhibitor-resistant cells [65]. The B-Raf gene was determined to be amplified in a subset of some treatment-naïve cells. The authors of this study determined that treatment with B-Raf and MEK inhibitors eliminated resistance of the cells. An additional study observed that the mutant *BRAF* V600E gene was amplified in 4 out of 20 melanoma patients which were resistant to B-Raf inhibitors [66]. This mechanism of B-Raf inhibitor-resistance is distinct from resistance generated by *NRAS* mutations or overexpression as the cells with amplified *BRAF* V600E were independent of Raf-1 expression while N-Ras-mediated inhibitor resistance was dependent on Raf-1 expression.

In an attempt to identify genes which could potentially confer resistance to B-Raf inhibitors, one group expressed a panel of approximately 600 kinase-related open reading frames in normally B-Raf inhibitor-sensitive A375 melanoma cells, which contain the *BRAF* V600E mutation [67]. This group identified mitogen-activated protein kinase kinase kinase 8 (*MAP3K8*) which encodes the serine-threonine protein kinase COT/Tp12 (cancer Osaka thyroid oncogene/ tumor progression locus-2) as a MAPK pathway agonist which drives resistance to Raf inhibition in *BRAF* mutant cell lines. COT was demonstrated to induce ERK via MEK but independent of Raf [67]. COT expression was observed to inversely correlate with *BRAF* V600E expression which may suggest that B-Raf may downregulate COT protein levels by destabilizing the protein. When *BRAF* V600E expression decrease due to B-Raf inhibitor treatment, the levels of COT are predicted to rise. Combining B-Raf and MEK inhibitors would overcome the resistance to the B-Raf inhibitors in the cells which overexpressed COT. The genomic region surrounding *MAP3K8* (COT) was amplified in 2 out of 38 *BRAF*-mutant cell lines. These lines had not previously been treated with B-Raf inhibitors. The lines with amplified *MAP3K8* (COT) were demonstrated to be resistant to B-Raf inhibitors. COT expression was determined to be increased in expression in some relapse patients. COT inhibitors are being developed and may be effective in overcoming the resistance present in some B-Raf inhibitor-resistant tumors [68].

The DNA sequences of 138 cancer genes from tumor cells isolated from a patient that initially was sensitive to the vemurafenib which became resistant after treatment were examined [50]. This study observed that there was a mutation in *MEK1* (C121S) in the vemurafenib-resistant tumor which was not present in the original tumor. The *MEK1* C121S mutation conferred resistance to both Raf and MEK inhibitors.

In another study with B-Raf inhibitor-resistant patient samples, the resistant cells were observed to have mutations at *NRAS* or overexpress PDGFR-beta [60]. These authors indicated that resistance to B-Raf inhibitors was not due to secondary mutations at *BRAF*, but activation of additional signaling pathways by PDGFR-beta or by N-Ras activation of the Raf/MEK/ERK pathway. PDGFR-beta was observed to be hyperphosphorylated in the cells from one B-Raf inhibitor-resistant line, but surprisingly the cells were not sensitive to imatinib which can target PDGFR-beta.

Other studies have indicated that switching of Raf isoforms may confer resistance to B-Raf inhibitors. Switching from B-Raf to either Raf-1 or A-Raf was observed after incubation of melanoma cells containing the *BRAF* V600E mutation in the presence of the B-Raf inhibitor dabrafenib for prolonged periods of time in the recovered inhibitor-resistant cells. In these inhibitor-resistant cells, they expressed other isoforms of Raf (*e.g*., Raf-1 or A-Raf) (69). In this study some inhibitor-resistant cells were also observed to overexpress IGF-1R which can also induce the expression of the PI3K/PTEN/Akt/mTOR pathway. Combined treatment with IGF-1R/PI3K and MEK inhibitors eliminated the resistance of the cells. Increased expression of IGF-1R and activation of Akt was also demonstrated in one of five paired specimens obtained from post-relapse vemurafenib-treated patients as compared to the patient samples prior to treatment.

Suppression of pro-apoptotic Bim expression is a mechanism of resistance to B-Raf inhibitors [70]. *PTEN*-mutant cells display decreased levels of Bim. Often melanoma cells with *BRAF* mutations also contain *PTEN* or *PIK3CA* mutations. Vemurafenib increases Bim expression in *PTEN* WT cells. The involvement of Akt-3 and FOXO3a was reported in these studies. Combining B-Raf and PI3K inhibitors enhanced Bim expression via FOXO3a in the *PTEN*-mutant cells.

In a study of Raf265-resistant melanomas containing the *BRAF* V600E mutation, it was observed that protein kinase D3 (PRKD3) mediated resistance to both Raf and MEK inhibitors and siRNA knockdown of PRKD3 cooperated with Raf265 in suppressing the growth of the resistant melanoma cells [71]. CID755673 is a PRKD3 inhibitor [72]. Potentially CID755673 could be combined with B-Raf inhibitors to suppress the growth of certain B-Raf inhibitor-resistant melanomas.

Dabrafenib-resistant A375 melanoma cells were isolated by culturing the cells in dabrafenib. The resistant cells were also resistant to vemurafenib and the MEK inhibitor trametinib (GSK112012, an allosteric MEK inhibitor), in frame deletions of *MEK1* and mutations at *NRAS* mutations were observed in some cells. The in frame deletions of *MEK* occurred at *MEK1* K59del, the *NRAS* mutations occurred at *NRAS* Q61K and A146T in the presence and absence of the *MEK1* P387S mutation in the A375 *BRAF* V600E line and *NRAS* Q61K in the YUSIT1 *BRAF* V600K line. The combination of dabrafenib and trametinib suppressed cell growth in the resistant lines. These results are somewhat surprising as some of the resistant lines had *NRAS* mutations. N-Ras could potentially activate PI3K/PTEN/Akt/mTOR pathway which could promote resistance to these inhibitors. The combination of the PI3K inhibitor GSK2126458 and either B-Raf or MEK inhibitors enhanced growth suppression and decreased ribosomal S6 protein phosphorylation [27]. Combination clinical trials are planned based on these results.

Two recent studies have indicated that the tumor microenviroment may contribute to the resistance to B-Raf and other small molecule inhibitors. The tumor microenviroment can secrete growth factors such as hepatocyte growth factor (HGF) which results in activation of the HGF receptor MET and subsequent downstream Raf/MEK/ERK and PI3K/PTEN/Akt/mTOR signaling which results in resistance to the small molecule inhibitors [73,74].

### MEK Inhibitors

Specific inhibitors of MEK have been developed: PD98059, PD184352 (a.k.a. CI-1040), PD0325901 (all Pfizer), U0126 (DuPont), Selumetinib (*a.k.a*., ARRY-142886, AZD6244) (Astra-Zeneca), MEK162/ARRY-162 (Novartis), GDC-0973 (Genentech), RDEA119/Refametinib (Ardea Biosciences/Bayer), GSK112012 (GlaxoSmithKlein), TAK-733 (Takeda San Diego, Millennium Pharmaceuticals, Inc), RO4987655 (Roche) and AS703026 (EMD Serono) [3-5,13,75-85].

MEK inhibitors differ from most other kinase inhibitors as they do not compete with ATP binding (non-ATP competitive), which confers a high specificity [75]. Most MEK inhibitors are specific and do not inhibit many different protein kinases [75-79] although as will be discussed below, certain MEK inhibitors are more specific than others. The crystal structures of MEK1 and MEK2 have been solved as ternary complexes with ATP and PD184352, and have revealed that both MEK1 and MEK2 have unique inhibitor binding sites located on a hydrophobic pocket adjacent to, but not overlapping with, the ATP-binding site [75]. Furthermore, effective targeting of MEK1/MEK2 is highly specific, as ERK1/ERK2 are the only well-described downstream targets. A distinct advantage of inhibiting MEK is that it can be targeted without knowledge of the precise genetic mutation that results in its aberrant activation. This is not true with targeting Raf as certain Raf inhibitors will activate Raf and also certain B-Raf-specific inhibitors will not be effective in the presence of *RAS* mutations as discussed above.

An advantage of targeting MEK is that the Ras/Raf/MEK/ERK pathway is a convergence point where a number of upstream signaling pathways can be blocked with the inhibition of MEK. For example, MEK inhibitors, such as selumetinib (AZD6244), are also being investigated for the treatment of pancreatic cancers, breast cancers, and other cancers such as hematopoietic malignancies, including multiple myeloma [75]. ClinicalTrials.gov lists 49 clinical trials for Selumetinib, either as a single agent or combined with another inhibitor or combinined with chemotherapy or radiotherapy.

Selumetinib inhibits MEK1 *in vitro* with an IC_50_ value of 14.1 ± 0.79 nM [79]; it is specific for MEK1 as it did not appear to inhibit any of the approximately 40 other kinases in the panel tested. Selumetinib is not competitive with ATP. Molecular modeling studies indicate that selumetinib binds to an allosteric binding site on MEK1/MEK2. The binding sites on MEK1/MEK2 are relatively unique to these kinases and may explain the high specificity of MEK inhibitors. This binding may lock MEK1/2 in an inactivate conformation that enables binding of ATP and substrate, but prevents the molecular interactions required for catalysis and access to the ERK activation loop. In basic research studies, treatment with the MEK inhibitor resulted in the detection of activated MEK1/2 when the western blot is probed with an antibody that recognizes active (phosphorylated) MEK1/2, while downstream ERK1/2 did not appear activated with the activation specific ERK1/2 antibody [13,79]. Selumetinib inhibited downstream ERK1/ERK2 activation in *in vitro* cell line assays with stimulated and unstimulated cells, and also inhibited activation in tumor-transplant models. Selumetinib did not prevent the activation of the related ERK5 that occurs with some older MEK1 inhibitors, which are not being pursued in clinical trials. Inhibition of ERK1/2 suppresses their ability to phosphorylate and modulate the activity of Raf-1, B-Raf and MEK1 but not MEK2 as MEK2 lacks the ERK1/ERK2 phosphorylation site. In essence, by inhibiting ERK1/2 the negative loop of Raf-1 and MEK phosphorylation is suppressed and hence there will be an accumulation of activated Raf-1 and MEK [13,79]. This biochemical feedback loop may provide a rationale for combining Raf and MEK inhibitors in certain therapeutic situations.

In colon, melanoma, pancreatic, liver and some breast cancers, selumetinib inhibited the growth of tumors in tumor xenograft studies performed in mice. The new MEK inhibitors are also at least 10 to 100-fold more effective than earlier MEK inhibitors and hence can be used at lower concentrations [13,79]. Selumetinib also inhibits the growth of human leukemia cells, but does not affect the growth of normal human cells. Selumetinib also suppressed the growth of pancreatic BxPC3 cells, which do not have a known mutation in this pathway, suggesting that this drug may also be useful for treating cancers that lack definable mutations. However, it is likely that BxPC3 cells have some type of upstream gene mutation/amplification or autocrine growth factor loop that results in activation of the Raf/MEK/ERK pathway.

Selumetinib induced G_1_/S cell-cycle arrest in colon and melanoma cancer cell lines and activated caspase-3 and -7 in some cell lines (Malme3M and SKMEL2); however, caspase induction was not observed in other melanoma (SKMEL28) or colon cancer cell lines (HT29), demonstrating that further research needs to be performed with this inhibitor to determine if it normally induces apoptosis and whether the induction of apoptosis can be increased with other inhibitors or chemotherapeutic drugs.

Selumetinib suppressed the tumor growth of pancreatic cells, such as BxPC3, in immunocompromised mice more effectively than conventional chemotherapeutic drugs, such as gemcitabine, which is commonly used to treat pancreatic cancer; however, once treatment with selumetinib was discontinued, the tumors reappeared [13,79]. Most likely MEK inhibitors do not induce apoptosis, but rather, they inhibit proliferation. That is, MEK inhibitors are cytostatic.

PD-184352 (Pfizer) was the first MEK inhibitor to enter clinical trials and it demonstrated inhibition of activated ERK and anti-tumor activity in patients [75]; however, subsequent multicenter, phase II studies with patients with diverse solid tumors did not demonstrate encouraging results [75]. This was probably due to low oral bioavailability and high metabolism, which led to plasma drug levels that were inadequate to suppress tumor growth.

The subsequent PD-0325901 MEK inhibitor is an orally-active, potent, specific, non-ATP competitive inhibitor of MEK. PD-0325901 demonstrated improved pharmacological and pharmaceutical properties compared with PD-184352, including a greater potency for inhibition of MEK, and higher bioavailability and increased metabolic stability. PD-0325901 has a K_i_ value of 1 nM against MEK1 and MEK2 in *in vitro* kinase assays. PD-0325901 inhibits the growth of cell lines that proliferate in response to elevated signaling of the Raf/MEK/ERK pathways [75]. Clinical trials with PD-0325901 have documented some successes and some adverse side effects [75]. MEK inhibitors may be appropriate to treat only those cancers that proliferate in response to activation of the Raf/MEK/ERK pathway [75]. Furthermore, it may also be important to include an additional pathway inhibitor, chemotherapeutic drug or radiation treatment to induce death of the cancer cell. There is a phase I clinical trial (NCT01347866) examining the effects of combining PD-0329501 with the PI3K/mTOR inhibitor PF-04691502. Initially this phase I trial will examine toxicity in patients with advanced cancers. If tolerable toxicity levels are observed, then additional studies will be perfomed with CRC patients containing mutant *KRAS* genes who have had previous therapy.

RDEA119/Refametinib is a more recently described MEK inhibitor developed by Ardea Biosciences [83-85]. It is a highly selective MEK inhibitor that displays a >100-fold selectivity in kinase inhibition in a panel of 205 kinases. In contrast, in the same kinase specificity analysis, other recently developed MEK inhibitors (*e.g.,* PD0325901) also inhibited the Src and RON kinases [83-85].

Trametinib (GSK1120212) is an allosteric MEK inhibitor developed by GSK. It has been shown to be effective when combined with dabrafenib in certain dabrafenib-resistant *BRAF* V600 melanoma lines that also had mutations at *NRAS* or *MEK1* [86]. The combination of trametinib and the PI3K/mTOR dual inhibitor GSK2126458 also enhanced cell growth inhibition in these B-Raf inhibitor-resistant *BRAF* mutant melanoma lines.

GDC-0973 (XL518) is a potent and selective MEK inhibitor developed by Genentech [78,87]. The effects of combining GDC-0973 and the PI3K inhibitor GDC-0941 on the proliferation of *BRAF* and *KRAS* mutant cancer cells indicated combination efficacy both *in vitro* and *in vivo*.

AS703026 (MSC1936369B) is a MEK inhibitor developed by EMD Serono. AS703026 suppressed cetuximab-resistant CRCs which had *KRAS* mutations both *in vitro* and *in vivo* models [88]. AS703026 inhibited growth and survival of multiple myeloma (MM) cells and cytokine-induced differentiation more potently than selumetinib and importantly AS703026 was cytotoxic, where as most MEK inhibitors are cytostatic [89]. AS703026 sensitized MM cells to a variety of conventional (dexamethasone, melphalan), and novel (lenalidomide, perifosine, bortezomib, rapamycin) drugs used to treat MM.

RO4987655 (CH4987655) is an allosteric, orally available MEK inhibitor developed by Roche/Chiron. It has been tested in humans and determined to inhibit active ERK levels. At the levels of RO4987655 administered, it was determined to be safe in healthy volunteers [90].

TAK-733 is a potent and selective, allosteric MEK inhibitor developed by Takeda San Diego [91]. TAK-733 is being investigated in clinical trials. MEK162 (ARRY-162) is a MEK inhibitor developed by Novartis. SL337 is a MEK inhibitor that has been used in many neurological and drug addiction studies [92].

### MEK Inhibitors in Clinical Trials

There are approximately 84 clinical trials with MEK inhibitors listed on the ClinicalTrials.gov website. Clinical trials have been and are being performed with various cancer patients and selumetinib (AZD6244), PD0325901, CI-1040, GSK1120212, TAK-733, RO4987655, MEK162, AS703026 and RHEA119. The MEK inhibitors may be appropriate for the treatment of certain melanomas which have mutant *BRAF* [62,93]. Phase II and III clinical trials have also been performed with the allosteric MEK inhibitor GSK1120212 (trametinib). GSK1120212 is in at least 27 clinical trials. NCT01037127 is a phase II clinical trial to examine the effectiveness of GSK112012 in melanoma patients containing a mutant *BRAF* gene. The trial will examine the effects of GSK112012 in either treatment-naïve or B-Raf inhibitor-treated patients. ARRY-438162 (MEK162) is a MEKinhibitor is currently in clinical trials in patients with advanced cancer. NCT0017925 is a phase I clinical trial with RDEA119 (BAY 86-9766) for patients with advanced cancers. NCT00957580 is a clinical trial with AS703026. Phase I will evaluate the effects of AS703026 on patients advanced hemtopoietic malignancies. Phase II is a continuation of the trial with AS703026 for elderly AML patients who are not good candidates for chemotherapy. The effects of MEK inhibitors on on patients with other cancers are also being examined in clinical trials.

Selumetinib is an orally-active MEK1 inhibitor that has undergone phase II clinical trials. It is one of the first MEK1 inhibitors to be evaluated in randomized phase II trials [75,93]. Selumetinib has demonstrated significant tumor suppressive activity in preclinical models of cancer, including melanoma, pancreatic, colon, lung, liver and breast cancer. The effects of selumetinib are enhanced significantly if the tumor has a mutation that activates the Ras/Raf/MEK/ERK signaling pathway. Selumetinib shows great promise in the treatment of pancreatic cancers, which often have mutations in Ras that can lead to downstream Raf/MEK/ERK pathway activation. Due to the frequent detection of pancreatic cancer at advanced stages, it may be necessary to combine signal transduction inhibitor therapy with conventional chemotherapy after surgical removal of the pancreatic cancer if possible. There is a clinical trial (NCT01222689) combining selumetinib and erlotinib (an EGFR inhibitor) in pancreatic cancer patients who have failed gemcitabine therapy. There are approximately 49 clinical trials with selumetinib listed on the Clinical.Trials.gov website.

There are approximately 84 clinical trials with MEK inhibitors listed on the Clinical.Trials.gov webite. There are 15 trials with MEK inhibitors and lung cancer, 14 trials with MEK inhibitors and pancreatic cancer, 10 trials with MEK inhibitors and colon cancers, 4 trials with MEK inhibitors and leukemias, 4 trials with MEK inhibitors and HCC, 4 trials with MEK inhibitors and brain cancers, 2 trials with MEK inhibitors and breast cancer and interestingly 0 trials with MEK inhibitors and prostate cancer. Initial results from clinical trials have not yielded overwhelming support for the use of MEK inhibitors as a single therapeutic agent in cancer patients who are not pre-screened for pre-existing activation of the Ras/Raf/MEK/ERK pathway [75,76,93]. Indeed, there are 21 clinical trials listed on the Clinical.Trials.gov website with MEK inhibitors and melanoma patients which often have mutation of *BRAF* and hence activation of downstream MEK. The proper pre-identification of cancer patients who display activation of the Raf/MEK/ERK pathway may be necessary for prescribing MEK inhibitors as part of their therapy, as we have stated previously that MEK inhibitors are cytostatic and not cytotoxic.

HCC is the 5^th^ most common cancer world-wide and there are few current effective therapies [11,80-83]. It is the 3^rd^ most common cause of cancer deaths world-wide and unfortunately it is the first in terms of cancer deaths in improvished countries. Targeting activated signaling and metabolic pathways have been considered as alternative approaches to treat HCC and improve therapy and outcomes [94-99].

Human HCC tumors have higher expression and enhanced activity of MEK1/2 and ERK1/2 compared with adjacent non-neoplastic liver [80]. Over-expression of activated MEK1 in HCC HepG2 cells resulted in enhanced tumor growth *in vivo* [81]. Preclinical studies have demonstrated the potential of MEK inhibition to suppress hepatoma cell proliferation and tumorigenicity [13]. Huynh et al. reported that treatment of human HCC xenografts with selumetinib blocked ERK1/2 activation, reduced *in vivo* tumor growth, and induced apoptosis [13]. Moreover, targeting MEK with PD-0325901 had *in vivo* chemopreventive effects on HCC development in an animal model employing TGF-alpha-transgenic mice in which liver cancers were induced by diethylnitrosamine treatment [82]. Therefore, MEK represents a potential therapeutic target for HCC.

### Dual Raf-MEK Inhibitors

Recently a dual B-Raf/Raf-1 and MEK inhibitor has been described [100]. RO5126766 is a first-in-class dual Raf/MEK inhibitor which allosterically inhibits B-Raf, Raf-1 and MEK. RO5126766 has a different mode of action than other Raf inhibitors as binds MEK and suppresses the phosphorylation of MEK by Raf via the formation of a stable Raf:MEK complex. RO5126766 selectively inhibited Raf and MEK and not any of the other 256 kinases in the Ambit KINOME panel. It was also show to be effective in suppressing the growth of certain human tumors with various combinations of mutated and WT *KRAS*/*HRAS* and *BRAF*. This inhibitor has been evaluated in a Phase I clinical trail [100]. Three partial responses were observed in fifty-two patients. Two *BRAF*-mutant melanoma patients responded and one *NRAS*- mutant melanoma patient responded. In contrast, to treatment with certain B-Raf inhibitors there were no cases of keratoacanthomas observed which the authors postulated was due to co-inhibitor of Raf and MEK. Dual Raf/MEK inhibitors may suppress the development of inhibitor resistance.

### MEK Inhibitor Resistance

Some tumors are resistant to MEK inhibitors because they contain *EGFR*, *KRAS*, *PI3KCA* or *PTEN* mutations [101-103]. Some cells with *EGFR* or *KRAS* mutations are resistant to MEK inhibitors as these mutant oncoproteins can also activate the Ras/PI3K/Akt/mTOR pathway. These studies, which were performed *in vitro* with cells lines and *in vivo* using xenografts, also demonstrated that PI3K activation and PTEN inactivation were not always equivalent in terms of inhibitor sensitivity. The authors suggested that a possible reason for this phenomenon could be that PTEN has other functions besides the regulation of Akt (*e.g*., protein phosphatase activity). Furthermore these studies demonstrated that the combination of MEK and PI3K pathway inhibitors could be an effective approach to treat certain cancers that had activation of both pathways.

Breast cancer affects nearly 1 in 7 women and is a diverse disease for which there is not one specific treatment which can be used to treat all patients. In addition, breast cancer patients often develop resistance to certain treatments such as hormonal, chemo-, radiotherapy perhaps due to the presence of CICs. Many genes have been implicated in breast cancer and sensitivity to therapy (e.g., *HER2*, *EGFR*, *ER*, *PIK3CA*, *BRCA*, *PTEN*, *TP53* and others) [104-108]. In addition, other genetic and epigenetic mechanisms have been implicated including deregulated expression of many other types of genes including tumor suppressors [109-120], cell cycle regulatory molecules [121], and more recently miRNA have been implicated in breast cancer [122-125]. In addition various physiological and genetic events may be altered or provoked in breast cancer and contribute to tumor progression and metastasis including: EMT [126], survival and expansion of CICs [127-130] genomic instability [131,132], epigenetic modifications [133,134], changes in the tumor microenvironment and stroma [135-143], angiogenesis [144], and senescence [145,146]. Thus there are many different genetic, biochemical and physiological processes which involved in breast cancer progression and scientists and clinicians have attempted to target various events. As we have stated previously, MEK is a common site of interaction of various signaling pathways, thus the ability to inhibit breast cancer by MEK inhibitors has been investigated.

Breast cancer can be classified into three types: luminal breast cancers which are usually ER+ and have a relatively good prognosis and response rate to hormonal based therapies, HER2+ cancers which have a poor prognosis if untreated but are initially responsive to herceptin, and basal-like breast cancers which have a poor prognosis and lack expression of HER2, estrogen and progesterone receptors (referred to as “triple-negative”).

Only certain types of breast cancer are sensitive to MEK inhibitors [102,103]. Many basal breast cancers express high levels of EGFR which results in activation of the Ras/Raf/MEK/ERK cascade. Hoeflich and colleagues [103] found that basal cell breast cancers expressed a Ras-like expression profile and tested their hypothesis that these breast cancers could be sensitive to MEK inhibitors, providing that they do not have *PI3KCA* mutations or *PTEN* deletions. In contrast, many luminal and HER2-amplified tumors are resistant to MEK inhibitors. They also determined that *PTEN* loss was a negative predictor factor for response to MEK inhibitors. Furthermore, treatment with MEK inhibitors often led to an increase in activated Akt expression, providing the rationale to examine the consequences of co-addition of MEK and PI3K inhibitors. The authors also determined that co-administration of MEK and PI3K inhibitors enhanced killing of the certain breast cancers. Thus the investigations by Wee *et al,* and Hoeflich *et al*., have demonstrated the concept that elevated PI3K/Akt/mTOR expression will confer resistance to MEK inhibitors. These studies illuminate the critical role of genetics in determining the sensitivity to targeted therapy.

Other studies have also indicated that some tumors with *EGFR* mutations are resistant to MEK inhibitors. Mutations at the *BRAF*, *KRAS*, *EGFR* genes or the chromosomal fusion between anaplastic lymphoma kinase (*ALK*) and *ROS* (oncogene that belongs to the sevenless subfamily of tyrosine kinase insulin receptor genes, originally detect in the v-ros retrovirus) tyrosine kinases are detected in approximately 50% of NSCLC. NSCLC cells with *BRAF* mutations where shown to be more sensitive to MEK inhibitors than NSCLC with mutations in *EGFR*, *KRAS*, or the chimeric fusion between *ALK* and *ROS* [101]. This was determined by screening a large panel of cell lines (n=87) and tumors (n=916). In this study, cells with mutations at *EGFR* were resistant to MEK inhibitors. This may have resulted from the ability of EGFR to activate the PI3K/PTEN/Akt/mTOR pathway which as discussed below has some crucial overlapping targets with the Raf/MEK/ERK pathway. NSCLC patients with *EGFR* mutations should not be treated with MEK (or BRAF) inhibitors as the respective therapies would be ineffectual.

In some MEK inhibitor-resistant melanoma cells which contained either the G469E or D594G mutant *BRAF* alleles, activation of Raf-1 by the mutant B-Raf proteins was observed to confer resistance to MEK inhibitors [18]. The G469E and D594G *BRAF* mutants are considered weak B-Raf mutations and signal through Raf-1. In these cells, survival is mediated by the G469E- and D594G-mutant B-Raf proteins stimulating Raf-1 which becomes mitochondrial localized and regulates apoptosis though phosphorylation of Bad and enhancement of the anti-apoptotic properties of Bcl-2. Sorafenib induced a reduction of Bad phosphorylation and Bcl-2 expression in the D594G/G469E melanoma cells. The effects of Raf-1 on the prevention of apoptosis were demonstrated in the D594G/G469E but not *BRAF* V600E mutant melanoma cells by shRNA knock down of Raf-1. These studies indicate that sorafenib may be appropriate in the treatment of a minority of melanomas which survive in response to Raf-1 activation and are essentially MEK inhibitor-resistant.

Amplification of a mutant *BRAF* gene in selumetinib-resistant CRCs was observed in cells which were selected for selumetinib-resistance *in vitro* [65]. The sensitivity of the cells to the MEK inhibitor could be restored by treatment with low doses of a B-Raf inhibitor. In this study, the authors demonstrated that the amplified mutant *BRAF* gene was present in a small minority of treatment-naïve cells. In another study by a different group of investigators, resistance to selumetinib was observed in CRC lines harboring mutations in *BRAF* (COLO205 and HT29) or *KRAS* (HCT116, LoVo). The selumetinib-resistant lines did not appear to have mutations in either *MEK1* or *MEK2* but had upregulation of B-Raf or K-Ras respectively due to intrachromosomal amplification of their respective driving oncogenes, *BRAF* V600E or *KRAS* G13D which the authors demonstrated was responsible for their selumetinib-resistance [147,148].

Mutations in the allosteric binding pocket of the *MEK1* gene were observed in a different study which isolated MEK-inhibitor resistant cells from MDA-MB-231 basal breast cancer cells [149]. Basal breast cancer cells are often sensitivity to MEK inhibitors. The MDA-MB-231 cell line has mutations at *BRAF* G464V and *KRAS* G13D. The MEK inhibitor-resistance could be overcome by treatment with ERK inhibitors, even in the resistant cell line with *KRAS* amplification.

Additional MEK-inhibitor resistant lines were derived from HCT-116 and LoVo CRC cell lines [149]. The MEK inhibitor-resistant HCT-116 cell line also had mutations in the allosteric binding pocket mutations in *MEK1* while the MEK inhibitor-resistant LoVo cells had mutations in the allosteric binding pocket in *MEK2*. One MEK inhibitor-resistant HCT-116 cell line also had the allosteric binding pocket mutation as well as amplification of *KRAS* but remained sensitive to growth inhibition upon treatment with the ATP-competitive ERK inhibitor, ERKi (name of inhibitor provided by authors in manuscript). These studies also demonstrated the effectiveness of inhibiting ERK in overcoming resistance to MEK inhibitors even if *BRAF* or *KRAS* is amplified or mutated. Furthermore the combination of MEK and ERK inhibitors may be beneficial in treating certain inhibitor-resistant cells.

### Combining Raf and MEK Inhibitors

The possibility of treating certain patients with a Raf and a MEK inhibitors is a concept which is gaining more acceptance as it may be a therapeutic possibility to overcome resistance [14]. Raf inhibitors induce Raf activity in cells with WT *RAF* if Ras is active, however, the addition of a MEK inhibitor would suppress the activation of MEK and ERK in the normal cells of the cancer patient. Thus B-Raf would be suppressed by the B-Raf-selective inhibitor in the cancer patient while the consequences of Raf activation in the normal cells would be suppressed by the MEK inhibitor. These concepts are being examined in clinical trials (NCT01072175 and NCT01231594). NCT01072175 is a clinical trial with the Raf inhibitor GSK2118436 in combination with the MEK Inhibitor GSK1120212 in metastatic melanoma patients containing mutant *BRAF* gene. NCT01352273 is a clinical trial with combinations of MEK162 and RAF265 examining the effects these MEK and Raf inhibitors on adult patients with solid tumors with either *RAS* or *BRAF* V600E mutations. The MEK inhibitor RDEA119/refametinib and sorafenib have been combined in Phase I/II clinical trials (NCT00785226) with patients having various types of advanced cancer. The dual Raf/MEK inhibitor RO5126766 has been in Phase I clinical trials [100].

The effects of combining MEK and Bcl-2/Bcl-X_L_ inhibitors have been examined in pre-clincial studies with AML cell lines and patient samples [150]. The Bcl-2/Bcl-X_L_ inhibitor ABT-737 was observed to induce ERK activation and Mcl-1 expression. However, when the ABT-737 inhibitor was combined with the MEK inhibitor PD0325901, a synergistic response was observed in terms of the induction of cell death both in AML cell lines and primary tumor cells with the properties of leukemia stem cells (a.k.a. CICs). Furthermore these studies were also extended into tumor transplant models with the MOLT-13 cell line and synergy between ABT-737 and PD0325901 were also observed *in vivo*.

### ERK Inhibitors

There are at least two ERK molecules regulated by the Raf/MEK/ERK cascade, ERK1 and ERK2. Little is known about the differential *in vivo* targets of ERK1 and ERK2. The development of specific ERK1 and ERK2 inhibitors is ongoing and may be useful in the treatment of certain diseases such as those leukemias where elevated ERK activation is associated with a poor prognosis (*e.g*., AML, ALL) [151]. ERK inhibitors have been described [152]. AEZS-131 has been reported on the internet to be a highly selective ERK 1/2 inhibitor developed by AEterna Zentaris. Other ERK inhibitors (ERKi) have also been developed and evaluated for their use in overcoming MEK inhibitor resistance [149].

### Inhibitors Targeting the PI3K/Akt/mTOR Pathway

**Figure 3:**
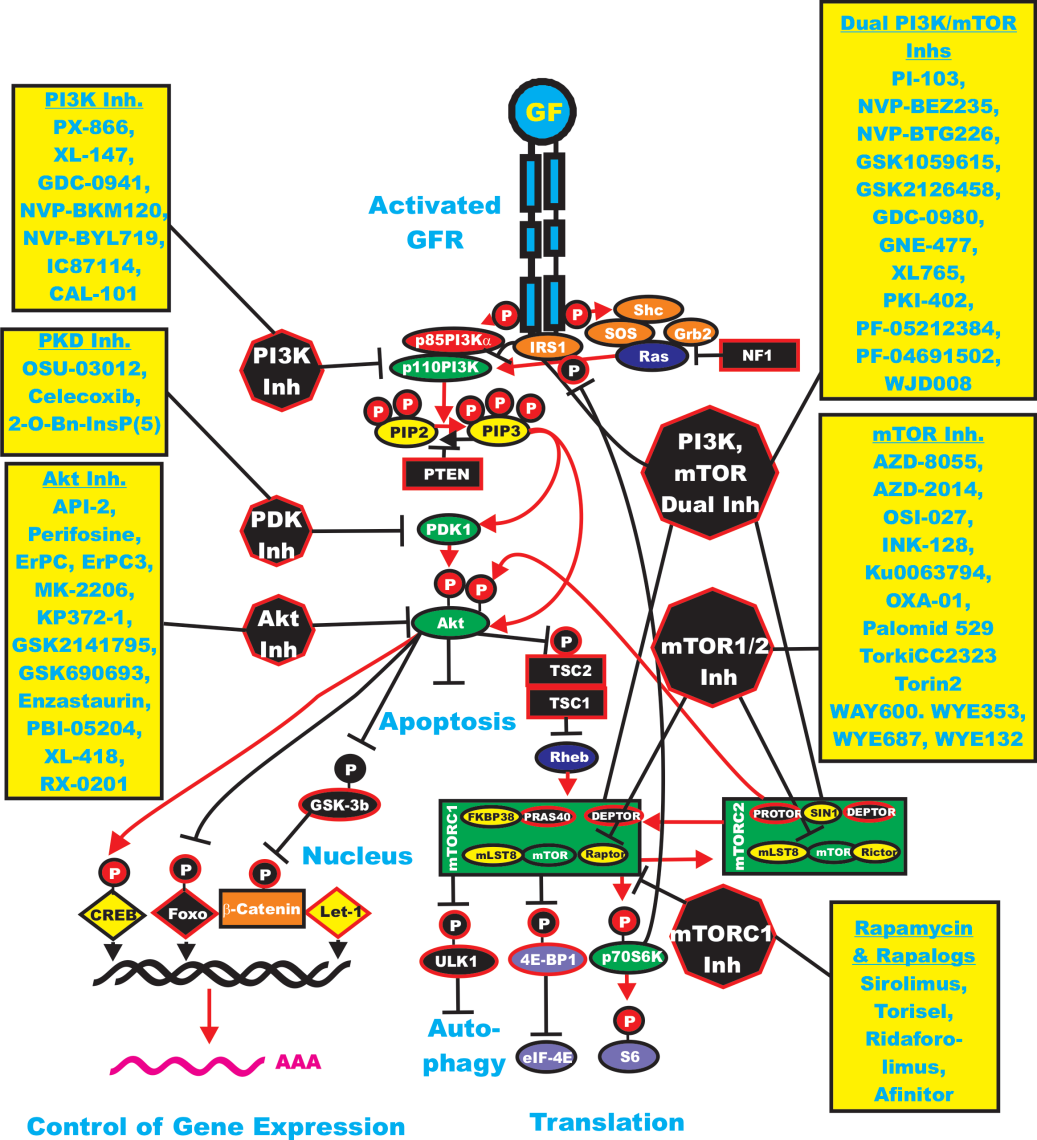
Overview of the PI3K/Akt/mTOR Cascade and Small Molecule Inhibitors Used for Targeting this Pathway Activation of this pathway can occur by mutations in upstream growth factor receptors (GFR) or by stimulation by the appropriate GF. In addition mutations can occur in intrinsic members of the pathway (*RAS PIK3CA*, *AKT* or the tumor suppressors (*NF1*, *PTEN*, *TSC1*, *TSC2*). Sites where NF1, PTEN, TSC1, TSC2 are depicted in black rectangles as they normally serve to dampen the activity of this pathway. An activated growth factor receptor is indicated in blue. Ras and Rheb are indicated in dark blue ovals. IRS1, SOS, Shc and Grb2 are indicated in orange ovals. Kinases are indicated in green ovals. The p85 regulatory subunit of PI3K is indicated in a red oval. Phosphatases are indicated in black octagons. NF1, TSC1 and TSC2 are indicated in black squares. PIP2 and PIP3 are indicated in yellow ovals. mTOR interacting proteins which positively regulate mTOR activity are indicated in yellor ovals. mTOR interacting proteins which negatively regulate mTOR activity are indicated in black ovals. Transcription factors activated by either ERK or Akt phosphorylation are indicated in yellow diamonds. The Foxo transcription factor that is inactivated by Akt phosphorylation is indicated by a black diamond. β-catenin is indicated in an orange rectangle. mRNA initiation factors and proteins associated with the ribosome are indicated in purple ovals. mTORC1 phosphorylates the unc-51-like kinase 1 (ULK1) which results in the suppression of autophagy. ULK1 is indicated in a black oval. The mTORC1 inhibitor prevents phosphorylation of ULK1 and autophagy can occur. Sites where various small molecule inhibitors function are in black octagons. Representative inhibitors are listed in boxes in yellow next to the octagons. Red arrows indicate activating events in pathways. Black arrows indicating inactivating events in pathway. Activating phosphorylation events are depicted in red circles with Ps with a black outlined circle. Inactivating phosphorylation events are depicted in black circles with Ps with a red outlined circle.

Numerous PI3K inhibitors have been developed and evaluated [3,4,6-10]. These include: LY-294002 (Lilly), Wortmannin, PX-866 (Oncothyreon), GDC-0941 (Genentech), CAL-101 (Calistoga Pharmaceuticals), XL-147 and XL-765 (Exelixis and Sanofi-Aventis). Some PDK1 inhibitors have been described but they are not specific for PDK1 including OSU-03012 (Arno Therapeutics) and Celecoxib (Pfizer). Various Akt inhibitors have been developed [153-157]. These include: A-443654 (Abbott Laboratories), GSK690693 (GlaxoSmithKline), VQD-002 (a.k.a. API-2, VioQuest Pharmaceuticals), KP372-1 (QLT, Inc), perifosine (AEterna Zentaris/Keryx Biopharmaceuticals) and MK-2206 (Merck). Inhibitors of downstream mTOR have been evaluated [158-161]. These include: rapamycin (Wyeth-Pfizer, sirolimus) and modified rapamycins (rapalogs) (CCI-779, torisel, temsirolimus, Wyeth-Pfizer), AP-23573 (ridaforolimus, Ariad-Merck) and RAD001 (afinitor, everolimus, Novartis). Rapamycin and the modified rapalogs are mTORC1 inhibitors. A diagram illustrating the sites of action of various inhibitors is presented in Figure 3.

### PI3K Inhibitors

Two well-known and isoform-nonselective PI3K inhibitors are the fungal metabolite wortmannin and LY294002. These drugs block the enzymatic activity of PI3K by different mechanisms. Wortmannin is an irreversible inhibitor (IC_50_≈2 nM) which forms a covalent bond with a conserved lysine residue involved in the phosphate-binding reaction [162], while LY294002 is a classical reversible, ATP-competitive PI3K modulator (IC_50_=1.40 μM) [163].

In spite of the crossover inhibition of other lipid and protein kinases (for example, LY294002 also inhibits mTOR, casein kinase 2 (CK2), DNA-dependent protein kinase (DNA-PK) and others) [164], and their unfavorable pharmaceutical properties, both wortmannin and LY294002 have served as important research tools for more than a decade in elucidating the role of PI3K in the biology of human cancer [165-170]. The modified wortmannin, PX-866 is a PI3K inhibitor [171,172]. It has been evaluated in Phase I clinical trials. PX-866 is currently being evaluated in approximately five clinical trials for prostate cancer, melanoma, CRC, NSCLC, squamous cell carcinoma of the head and neck, glioblastoma and other advanced cancers.

GDC-0941 is a PI3K inhibitor developed by Genentech. GDC-0941 inhibited the metastatic characteristics of thyroid carcinomas by targeting both PI3K and hypoxia-inducible factor-1alpha (HIF-1-alpha) pathways [173]. GDC-0941 synergized with the MEK inhibitor UO126 in inhibiting the growth of NSCLC [174]. It is being evaluated in a clinical trial for advanced cancers or metastatic breast cancers which are resistant to aromatase inhibitor therapy (NCT01437566).

IC87114 is a selective p110-delta PI3K inhibitor. It decreased cell proliferation and survival in AML cells, and increased sensitivity to etoposide [175-179]. It has been in clinical trials to treat AML patients (NCT00004263 and NCT00301938). CAL-101(GS-1101) is a derivative of IC-87114 [180-184]. It is an oral p110-delta PI3K inhibitor developed by Calistoga Pharmaceuticals and Gilead Sciences. CAL-101 is currently undergoing clinical evaluation in patients with various hematopoietic malignancies including: relapsed or refractory indolent B-cell NHL, mantle cell lymphoma or CLL. An additional clinical trial, will examine the effects of combining CAL-101 with chemotherapeutic drugs and the αCD20 monoclonal Ab (MoAb). The clinical trial (NCT01088048) will examine the effects of combining CAL-101 with chemotherapeutic drugs and the αCD20 monoclonal Ab. CAL-101 has displayed significant cytotoxic activity in 23% of B-ALL samples tested, but only in 3% of AML samples. CAL-101 treatment resulted in dephosphorylated Akt-1 at T308 and induced apoptosis in neoplastic B-cells [181]. Remarkably, CAL-101 did not significantly affect the survival of healthy B-, T-, and natural killer (NK) lymphocytes [182]. However, it was found that CAL-101 inhibited the production of inflammatory cytokines, such as interleukin-6 (IL-6), IL-10, tumor necrosis factor (TNF)-alpha (produced by T-lymphocytes), and interferon (IFN)-gamma (synthesized by NK lymphocytes). It remains to be established whether decreased production of TNF-alpha and IFN-gamma will impair inflammatory responses in B-ALL patients treated with CAL-101.

XL-147 (SAR245408) is a PI3K inhibitor developed by Exelixis/Sanofi-Aventis [183]. 2010). It is in at least 11 clinical trials, either as a single agent or in combination with erlotinib, hormonal therapy, chemotherapy, or MoAb therapy for various cancers including: lymphoma, breast, endometrial, glioblastoma, astrocytoma or other solid cancers.

NVP-BKM120 (BKM120) is an orally available pan-class I PI3K inhibitor developed by Novartis [184,185]. It is in clinical trials, either as a single agent or in combination with other drugs or signal transduction inhibitors [185]. NVP-BKM120 is in at least 36 clinical trials with patients having advanced cancers such as CRC, NSCLC, breast, prostate, endometrial, squamous cell carcinoma of the head and neck, GIST, RCC, melanoma and advanced leukemias.

NVP-BYL719 (BYL719) is a PI3K-alpha selective inhibitor developed by Novartis. It is in clinical trials for patients with advanced solid tumors (NCT01387321) some containing mutations at *PIK3CA* (NCT01219699). It is also being examined in a clinical trial in combination with the MEK-162 inhibitor for patients with advanced CRC, esophageal, pancreatic, NSCLC or other advanced solid tumors containing *RAS* or *BRAF* mutations (NCT01449058). Some have questioned whether inhibitors which target just PI3K will be effective in cancer therapy as single agents due to in part the complicated feed-back loops which result in the activation of certain receptor molecules [186].

### Dual PI3K/mTOR Inhibitors

The catalytic sites of PI3K and mTOR share a high degree of sequence homology. This feature has allowed the synthesis of ATP-competitive compounds that target the catalytic site of both PI3K and mTOR. Several dual PI3K/mTOR inhibitors have been developed. In preclinical settings, dual PI3K/mTOR inhibitors displayed a much stronger cytotoxicity against leukemic cells than either PI3K inhibitors or allosteric mTOR inhibitors, such as rapamycin or rapalogs. In contrast to rapamycin/rapalogs, dual PI3K/mTOR inhibitors targeted both mTOR complex 1 and mTOR complex 2, and inhibited the rapamycin-resistant phosphorylation of eIF4B-1 and inhibited protein translation of many gene products associated with oncogenesis (enhanced proliferation) in leukemic cells. The dual inhibitors strongly reduced the proliferation rate and induced an important apoptotic response [7].

The kinase selectivity profile of the dual PI3K/mTOR modulators is consistent with the high sequence homology and identity in the ATP-catalytic cleft of these kinases. Dual PI3K/mTOR inhibitors have demonstrated significant, concentration-dependent cell proliferation inhibition and induction of apoptosis in a broad panel of tumor cell lines, including those harboring *PIK3CA* activating mutations [187].

Moreover, the *in vitro* activity of these ATP-competitive PI3K/mTOR modulators has translated well in *in vivo* models of human cancer xenografted in mice. They were well tolerated and achieved disease stasis or even tumor regression when administered orally [188]. In spite of their high lipophilicity and limited water solubility, the pharmacological, biological and preclinical safety profiles of these dual PI3K/mTOR inhibitors supported their clinical development [189].

There may be some benefits to treating patients with an inhibitor that can target both PI3K and mTOR as opposed to treating patients with two inhibitors, *i.e*., one targeting PI3K and another specifically mTOR. An obvious benefit could be lowered toxicities. Treatment with a single drug could have fewer side effects than treatment with two separate drugs. The effects of detrimental Akt activation by mTOR inhibition might be avoided upon treatment with a dual kinase inhibitor. Furthermore, the negative side effects of mTOR inhibition on the activation of the Raf/MEK/ERK pathway might be eliminated with the PI3K inhibitor activity in the dual inhibitor. There remains, however, considerable uncertainty about potential toxicity of compounds that inhibit both PI3K and mTOR enzymes whose activities are fundamental to a broad range of physiological processes. Although it should be pointed out that there are some clinical trials in progress to determine whether it is beneficial to treat cancer patients with a PI3K/mTOR dual inhibitor and an mTORC1 blocker such as NVP-BEZ235 and RAD001. Pre-clinical studies have documented the benefits of combining RAD001 with NVP-BEZ235 [190].

PI-103 was the first reported ATP-competitive kinase inhibitor of mTOR which also blocked the enzymatic activity of PI3K p110 isoforms. It was developed at UCSF in 2006. PI-103 exhibits good selectivity over the rest of the human kinome in terms of non-selective inhibition of other kinases [191,192]. PI-103 is a pan-class I PI3K inhibitor with IC_50_ values in the 2 nM (p110-alpha PI3K) to 15 nM range (p110-gamma PI3K) PI-103 inhibits both mTORC1 (IC_50_=0.02 microM) and mTORC2 (IC_50_=0.083 microM).

NVP-BEZ235 is a dual PI3K/mTOR inhibitor developed by Novartis. Importantly and in contrast to rapamycin, NVP-BEZ235 inhibited the rapamycin-resistant phosphorylation of 4E-BP1, causing a marked inhibition of protein translation in AML cells. This resulted in reduced levels of the expression of c-Myc, cyclin D1, and Bcl-x_L_ known to be regulated at the translation initiation level [193]. NVP-BEZ235 suppressed proliferation and induced an important apoptotic response in AML cells without affecting healthy CD34^+^ cell survival. Importantly, it suppressed the clonogenic activity of leukemic, but not healthy, CD34^+^ cells [194]. NVP-BEZ235 targeted the side population (SP) of both T-ALL cell lines and patient lymphoblasts, which might correspond to CICs, and synergized with several chemotherapeutic agents (cyclophosphamide, cytarabine, dexamethasone) currently used for treating T-ALL patients [195]. Also, NVP-BEZ235 reduced chemoresistance to vincristine induced in Jurkat cells by co-culturing with MS-5 stromal cells, which mimic the bone marrow microenvironment [196]. In this study, NVP-BEZ235 was cytotoxic to T-ALL patient lymphoblasts displaying pathway activation, where the drug dephosphorylated 4E-BP1, in contrast to the results obtained with rapamycin. Taken together, these findings indicated that longitudinal inhibition at two nodes of the PI3K/Akt/mTOR network with NVP-BEZ235, either alone or in combination with chemotherapeutic drugs, may be an effective therapy for of those T-ALLs that have aberrant upregulation of this signaling pathway.

NVP-BEZ235 has been evaluated also in a mouse model consisting of BA/F3 cells overexpressing either WT *BCR-ABL* or its imatinib-resistant *BCR-ABL* mutants (E255K and T315I) [197]. NVP-BEZ235 inhibited proliferation of both cytokine-independent WT *BCR-ABL* and mutant *BCR-ABL* (E255K and T315I) overexpressing cells, whereas parental cytokine-dependent Ba/F3 cells were much less sensitive. The drug also induced apoptosis, and inhibited both mTORC1 and mTORC2 signaling. Remarkably the drug displayed cytotoxic activity *in vivo* against leukemic cells expressing the E255K and T315I *BCRABL* mutant forms However, in this experimental model, NVP-BEZ235 induced an over activation of MEK/ERK signaling, most likely due to the well-known compensatory feedback mechanism that involves p70S6K [198]. NVP-BEZ235 has been intensively investigated and is in at least eight clinical trials for patients with advanced cancers [199]. NCT01343498, NCT01195376 and NCT01513356 are clinical trials of NVP-BEZ235 as a single agent in patients with advanced solid tumors including breast. In the clinical trial NCT00620594, NVP-BEZ235 is being evaluated in breast cancer patients, some of whom may also be treated with herceptin. NCT01285466 is a clinical trial for patients with advanced solid cancers who will be treated with NVP-BEZ235, paclitaxel and herceptin. NVP-BTG226 is a recently developed PI3K/mTOR inhibitor by Novartis.

PKI-587 is a PI3K/mTOR inhibitor developed by Pfizer [201,202]. It is also known as PF-05212384 and it inhibits class I PI3Ks, PI3K-alpha mutants, and mTOR. PKI-587 suppressed proliferation of approximately 50 diverse human tumor cell lines with IC_50_ values less than 100 nmol/L. PKI-587 induced apoptosis in cell lines with elevated PI3K/Akt/mTOR signaling. PKI-587 inhibited the tumor growth in various models including: breast (MDA-MB-361, BT474), colon (HCT116), lung (H1975), and glioma (U87MG). The efficacy of PKI-587 efficacy was enhanced when administered in combination with the MEK inhibitor, PD0325901, the topoisomerase I inhibitor, irinotecan, or the HER2 inhibitor, neratinib.

PF-04691502 is an ATP competitive PI3K/Akt inhibitor developed by Pfizer which suppresses activation of Akt [202]. PF-04691502 suppressed transformation of avian cells in response to either WT or mutant *PIK3CA*. PF-04691502 inhibited tumor growth in various xenograft models including U87 (*PTEN* null), SKOV3 (*PIK3CA* mutant), and gefitinib (EGFR inhibitor) and erlotinib-resistant NSCLC [202]. Both PKI-587 and PF-04691502 are in clinical trials with patients having endometrial cancers (NCT01420081).

PKI-402 is a selective, reversible, ATP-competitive, PI3K and mTOR inhibitor developed by Pfizer. It suppresses mutant PI3K-alpha and mTOR equally. PKI-402 inhibited the growth of many human tumor cell lines including: breast, glioma, pancreatic, and NSCLC [203].

XL765 (SAR25409) is a dual PI3K/mTOR inhibitor developed by Exelixis/Sanofi-Aventis. XL765 has been investigated in brain and pancreatic cancer models either as a single agent or in combination with temozolomide [204] or the autophagy inhibitor chloroquine [205]. XL765, downregulated the phosphorylation of Akt induced by PI3K/mTORC2 and reduced brain tumor growth [204]. Combining XL765 with chloroquine suppressed autophagy and induced apoptotic cell death in pancreatic tumor models [205]. XL-147 (SAR245408) and XL-765 (SAR245409) are in at least 13 clinical trials, either as a single agent or in combination with erlotinib, hormonal therapy, chemotherapy, or MoAb therapy for various cancers including: lymphoma, breast, endometrial or other solid cancers. NCT01240460 is a clinical trial for recurrent glioblastoma and astrocytoma grade IV patients who are candidates for surgical resection by Exelixis and Sanofi-Aventis. XL765 (Exelixis/Sanofi-Aventis) [204] has been in clinical trials either as single agent (NCT00485719) to treat patients with advanced tumors. In one study XL765, downregulated the phosphorylation of Akt induced by PI3K/mTORC2 and reduced tumor growth. XL765 also resulted in clinical benefit in 5 out of 19 patients [188]. Other clinical trials are being performed with XL765 in combination with temozolomide to treat patients with glioblastoma (NCT00704080) or in combination with erlotinib to treat NSCLC patients (NCT00777699).

GNE-477 is a dual PI3K/mTOR inhibitor developed by Genentech. GDC-0980 is similar to GNE-477 and has high activity in cancer models driven by PI3K pathway activation [206]. GDC-0980 is in a clinical trial for patients with advanced cancers or metastatic breast cancers which are resistant to aromatase inhibitor therapy (NCT01437566).

GSK2126458 is a dual PI3K/mTOR inhibitor developed by GSK [80]. It is in at least two clinical trials with advanced cancer patients. In one trial it is being combined with the MEK inhibitor GSK1120212. GSK1059615 is a dual PI3K/mTOR inhibitor developed by GSK. It was in a clinical trial with patients with solid tumors, metastatic breast cancer, endometrial cancers and lymphomas which was terminated.

WJD008 (Chinese Academy of Sciences, Shanghai) is a dual PI3K/mTOR [207]. WJD008 inhibited the increased activity of the PI3K pathway normally induced by *PIK3CA* H1047R and suppressed proliferation and colony formation of transformed RK3E cells containing *PIK3CA* H1047R.

### Resistance to PI3K/mTOR Inhibitors

**Figure 4:**
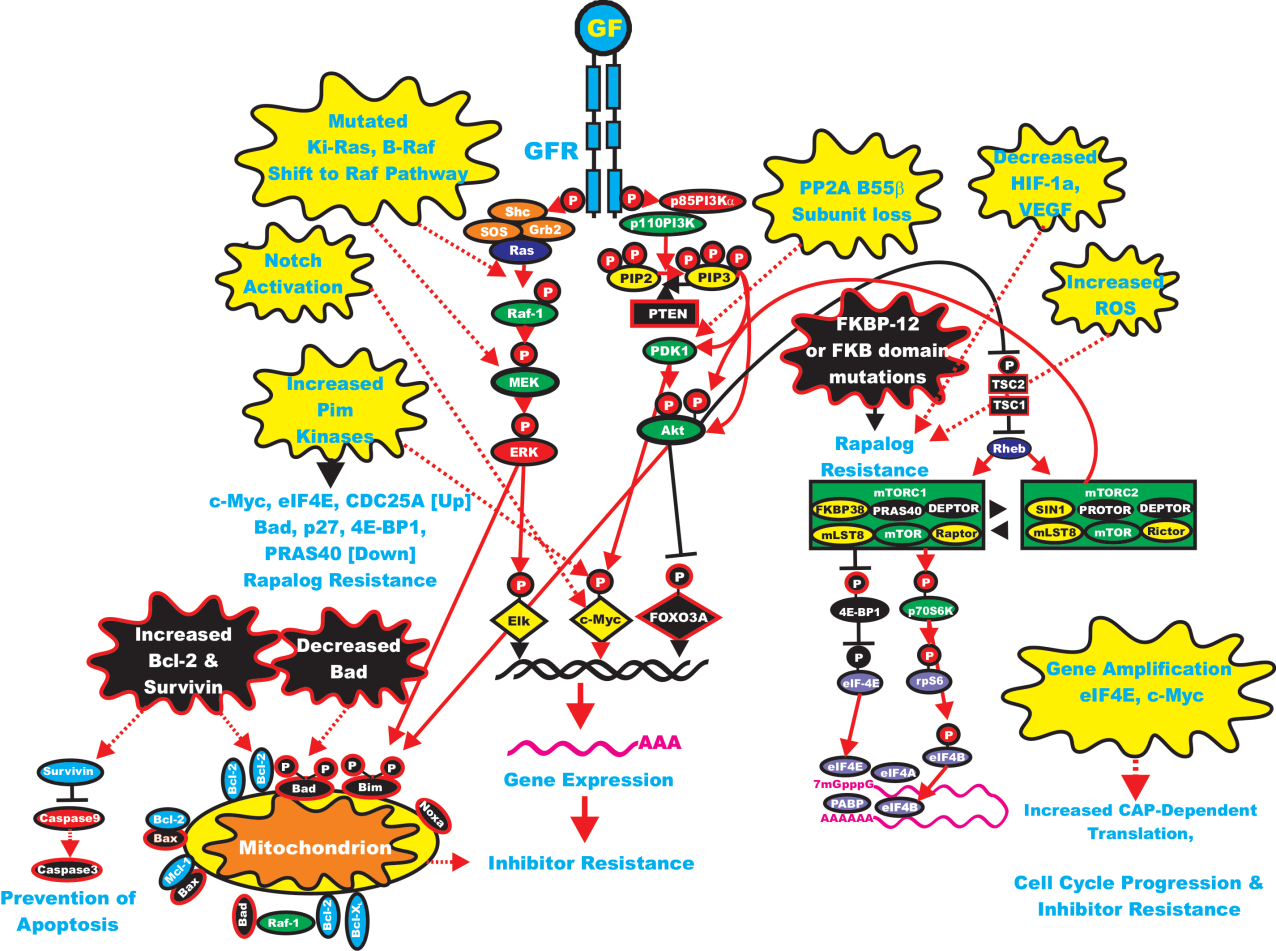
Sites of Mutation which can Result in Resistance to mTOR Inhibitors Sites of mutation which result in resistance to mTOR inhibitors are indicated in yellow irregular circles. The same color scheme present in Figures 1, 2 and 3 is continued in this figure.

Recently resistance to PI3K/mTOR inhibitors has been observed. In one case, c-Myc and eIF4E amplification were observed which result in elevated 5'cap-dependent protein translation in human mammary epithelial cells that were resistant to NVP-BEZ235 [208]. In another study, the authors observed NOTCH pathway, including downstream c-Myc activation which eliminated the dependency of the cells on the PI3K/Akt/mTOR pathway [209]. NOTCH is frequently activated in T-ALL [6-10]. This may explain the resistance of some ALLs to PI3K inhibitors. c-Myc is frequently amplified in certain cancers [3-6]. This may contribute to the inherent resistance of this type of tumor to PI3K inhibitors [209]. A diagram illustrating potential mechanisms of resistance to PI3K/mTOR inhibitors is presented in Figure 4.

### PDK Inhibitors

Some compounds have been reported to be PDK inhibitors, including the modified celecoxib, OSU-03012 [210, 211] and 2-O-BN-InsP(5) [212]. Celecoxib (Celebrex, Pfizer) obviously has other targets than PDK, such as cyclooxygenase-2 (Cox-2). Celecoxib is used to treat CRC patients to reduce the number of polyps in the colon. OSU-03012 is reported not to inhibit Cox-2 [210]. 2-O-BN-InsP(5) is based on the structure of based on the structure of inositol 1,3,4,5,6-pentakisphosphate, it may inhibit both PDK and mTOR [212].

### Akt Inhibitors

Many attempts to develop Akt inhibitors have been performed over the years. In many of the earlier attempts, the various Akt inhibitors either lacked specificity or had deleterious side effects. Part of their deleterious side effects of many “Akt” inhibitors are probably related to the numerous critical functions that Akt plays in normal physiology. Namely some Akt inhibitors will alter the downstream effects of insulin on Glut-4 translocation and glucose transport.

Triciribine (API-2) is an Akt inhibitor that has been used in many studies; at least 92 are listed on the ClinicalTrials.gov website. Triciribine suppressed phosphorylation of all three Akt isoforms *in vitro* and the growth of tumor cells overexpressing Akt in mouse xenograft models [213]. The mechanism(s) by which triciribine inhibits Akt activity are not clear. The drug has been evaluated in a phase I clinical trial in patients with advanced hematologic malignancies, including refractory/relapsed AML. In this trial (NCT00642031), triciribine was administered on a weekly schedule. The drug was well-tolerated, with preliminary evidence of pharmacodynamic activity as measured by decreased levels of activated Akt in primary blast cells [214]. Triciribine has also been examined in a clinical trial (NCT00363454) with Akt+ metastatic cancers.

MK-2206 (Merck) is an allosteric Akt inhibitor which inhibits both T308 and S473 phosphorylation. It also inhibits the downstream effects of insulin on Glut-4 translocation and glucose transport [215]. MK-2206 decreased T-acute lymphocytic leukemia (T-ALL) cell viability by the blocking the cells in the G_0_/G_1_ phase of the cell cycle and inducing apoptosis. MK-2206 also induced autophagy in the T-ALL cells. MK-2206 induced a concentration-dependent dephosphorylation of Akt and its downstream targets, GSK-3-alpha/beta and FOXO3A. MK-2206 also was cytotoxic to primary T-ALL cells and induced apoptosis in a T-ALL patient cell subset (CD34^+^/CD4^−^/CD7^−^) which is enriched in CICs. [216]. MK-2206 is in at least 43 clinical trials either as a single agent or in combination with other small molecule inhibitors or chemotherapeutic drugs with diverse types of cancer patients.

GSK690693 is a pan Akt inhibitor developed by GSK. GSK690693 is an ATP-competitive inhibitor effective at the low-nanomolar range. Daily administration of GSK690693 resulted in significant antitumor activity in mice bearing various human tumor models including SKOV-3 ovarian, LNCaP prostate, and BT474 and HCC-1954 breast carcinoma. The authors also noted that GSK690693 resulted in acute and transient increases in blood glucose level [217]. The effects of GSK690693 were also examined in 112 cell lines representing different hematologic neoplasia. Over 50% of the cell lines were sensitive to the Akt inhibitor with an EC_50_ of less than 1 μM. ALL, non-Hodgkin lymphomas, and Burkitt lymphomas exhibited 89%, 73%, and 67% sensitivity to GSK690693, respectively. Importantly GSK690693 did not inhibit the proliferation of normal human CD4^+^ peripheral T lymphocytes as well as mouse thymocytes.

GSK2141795 is an Akt inhibitor under development at GSK. It is reported by GSK to be an oral, pan Akt inhibitor which shows activity in various cancer models, including blood cancers and solid tumor models. In addition it is reported by GSK to delay tumor growth in solid tumor mouse xenograft models. It has been investigated further in clinical trials.

KP372-1 (QLT, Vancouver, Canada) inhibits PDK1, Akt and Fms-like tyrosine kinase 3 (Flt-3) signaling and induces mitochondrial dysfunction and apoptosis in AML cells but not normal hematopoietic progenitor cells [218]. It also suppressed colony formation of primary AML patient sample cells but not normal hematopoietic progenitor cells. It has also been investigated in other cancer types, including squamous cell carcinomas of the head and neck, thyroid cancers and glioblastomas.

Enzasturin (LY317615) is a protein kinase C-beta (PKC-beta) and Akt inhibitor developed by Lilly. It has been investigated in clinical trials either by itself or in combination with other agents in various types of cancer patients including: brain [219] and NSCLC [220], CRC [221] as well as other cancer types. It is reported to be in approximately 48 clinical trials on the ClinicalTrials.gov website.

Perifosine (KRX-0401, Keryx / AOI Pharmaceuticals, Inc. and licensed to AEterna Zentaris) is an alkylphospholipid that can inhibit Akt [222]. The effects of perifosine have been examined on many different tumor types. Perifosine induces caspase-dependent apoptosis and downregulates P-glycoprotein expression in multidrug-resistant T-ALL cells by a JNK-dependent mechanism [223]. Perifosine is or has been in at least 43 clinical trials to treat various cancer patients, with either blood cancers or solid tumors, either by itself, or in combination with other agents. It has advanced to phase III clinical trials for CRC and MM. In the USA it has orphan drug status for the treatment of MM and neuroblastoma.

Erucylphosphocholine (ErPC) and erucylphosphohomocholine (ErPC3) have been shown to inhibit Akt and induce apoptosis in malignant glioma cell lines which are normally resistant to the induction of apoptosis. They are structurally related to perifosine [224]. ErPC enhanced radiation-induced cell death and clonogenicity [225]. These effects on the induction of apoptosis were correlated with increased Bim levels and decreased Bad and Foxo-3 phosphorylation, potentially consequences of decreased Akt activity. ErPC3 is the first intravenously applicable alkylphosphocholine. ErPC3 was cytotoxic to AML cells through JNK2- and PP2-dependent mechanisms [226].

PBI-05204 (oleandrin) is an Akt inhibitor. PBI-05024 is a botanical drug candidate derived from *Nerium oleander* and developed by Phoenix Biotechnology. It also has other targets including FGF-2, NF-kappaB, and p70S6K. PBI-05204 is in clinical trials for cancer patients with advanced solid tumors [227]. Interesting PBI-05204 also provides significant neuroprotection to tissues damaged by glucose and oxygen deprivation which occurs in ischemic stroke [228].

RX-0201 (Akt1AO, Rexahn Pharmaceuticals, Inc.) is an Akt-1 anti-sense oligonucleotide molecule. RX-0201 downregulated Akt-1 expression at nanomolar concentrations in multiple types of human cancer. RX-0201 also inhibited tumor growth in mice xenografted with U251 human glioblastoma and MIA human pancreatic cancer cells [229]. RX-021 has been in a clinical trial in combination with gemcitabine for patients with metastatic pancreatic cancer [230].

XL-418 is reported to be a dual Akt/p70S6K inhibitor by developed by Exelixis/GSK. It was in clinical trials for patients with advanced cancer, however those trials were suspended.

### mTORC1 Inhibitors

Rapamycin (Rapamune, Pfizer) was approved by the FDA in 1999 to prevent rejection in organ transplant patients. Rapamycin/rapalogs act as allosteric mTORC1 inhibitors and do not directly affect the mTOR catalytic site [6,7]. They associate with the FK506 binding protein 12 (FKBP-12) and by so doing, they induce disassembly of mTORC1, resulting in repression of its activity [231,232]. The rapalogs have been examined in clinical trials with patients having various cancers including: brain, breast, HCC, leukemia, lymphoma, MM, NSCLC, pancreatic, prostate, and RCC [233, 234]. Furthermore rapamycins are being considered as anti-aging and anti-obestity drugs as well as to prevent diabetic neuropathy [236-239].

The rapalogs torisel (Pfizer) amd afinitor (Novartis) were approved in 2007 and 2009 (respectively) to treat RCC patients [240]. In 2008, torisel was approved to treat Mantel cell lymphoma patients. In 2010, Afinitor was approved to treat subependymal giant cell astrocytoma (SEGA) tumors in tuberous sclerosis (TS) patients. In 2011, Afinitor was approved to treat patients with pancreatic neuroendocrine tumors [241].

Ridaforolimus (also known as AP23573 and MK-8669; formerly known as deforolimus) is a rapalog developed by ARIAD and Merck. Ridaforolimus has been evaluated in clinical trials with patients having metastatic soft-tissue or bone sarcomas where it displays promising results in terms of the risk of progression or death [242]. Recently the ability of rapamycin and rapalog to treat various viral infections including AIDS has been considered [243,244]. Clearly rapamycin has proven to be a very useful drug. In addition, novel approaches to target mTORC have been developed (see below).

### Sensitivity to Rapamycin

Multiple mechanisms have been described to be responsible for sensitivity to rapamycin [245]. Rapamycin sensitivity has been associated with *PTEN* mutation/silencing (inactivation), *PIK3CA* mutation (activation) and Akt hyperactivation. RCC patients are hypersensitive to rapalogs as they often have loss of function of the von-Hippel-Lindau (*VHL*) tumor suppressor gene which is an E3 ubiquitin ligase that promotes the proteasomal degradation of HIF-1-alpha and HIF-1-beta [246]. Rapalogs promote reduction of HIF-1-alpha levels, thus RCC cells can not survive and are hyper-sensitive to rapalogs [247]. Mantel cell lymphoma grown in part due to increased levels of cyclin D1. mTOR inhibitors suppress cyclin D1 mRNA translation, thus Mantel cell lymphomas are hypersensitive to rapalogs [248]. Inhibition of IGF-1R signaling increases sensitivity to mTOR inhibitors.

### Resistance to Rapamycin/Rapalogs

Resistance to rapamycin has been associated with *KRAS* or *BRAF* mutations. Since *KRAS* is frequently mutated in human cancer, many cancers will have constitutive mTOR activity, but may not be sensitive to rapamycin as they will have Raf/MEK/ERK pathway activation. Since rapalogs function by binding FKBP-12, mutations in *FKBP12* or the FKB domain of mTOR can suppress binding affinity and lead to rapalog resistance [245,249-251]. Direct mTOR inhibitors will overcome this resistance. The presence of the IGF1R/PI3K-mediated feedback loop, which results in ERK activation, is another mechanism of resistance to rapamycin rapalogs [3,4,6-10].

Up regulation of the PIM kinases is another mechanism of resistance to rapalogs [252]. The PIM family of oncogenic serine/threonine kinases play important roles in the regulation of cell growth [253,254] Pim kinases have multiple substrates important in the regulation of cell growth including: c-Myc, p27, dual specificity phosphatase CDC25A and Bad [252].

Pim kinases also stimulate mTORC1 activity by phosphorylation of 4E-BP1, eIF4E and PRAS [255-258]. PDK1 activation also results in resistance to rapalogs [259]. This results in PDK1 phosphorylation of c-Myc after rapamycin treatment. Altering the levels of 4E-BP1 (decreasing) or eIF4E (increasing) can result in resistance to rapamycin [260]. Some cells deficient in p27^Kip-1^ are resistance to rapamycin as rapamycin normally prevents p27^Kip-1^ down regulation [261]. There are other mechanisms of resistance to rapamycin. One group has determined that the levels of cyclin E-dependent kinase activity are altered in resistant hepatic cells [262]

Increased oxidative stress induces mTORC1 modification which prevents its ability to bind the FKBP-12/rapamycin complex [263]. High levels of reactive oxygen species (ROS) promote resistance to rapalogs. mTOR kinase inhibitors may be able to inhibit ROS mediated rapalog resistance as they inhibit mTOR independently of FKBP-12 [264]. Overexpression of Bcl-2 and survivin can make certain cells resistant to the apoptosis normally induced by rapalogs [265]. Inhibition of angigogenesis is a potent aspect of rapalogs *in vivo* [266]. Since HIF-1-alpha controls VEGF expression, tumors with decreased VEGF expression are more resistant to rapalogs. There are other strategies to overcome mTOR resistance being examined. The effects of combined dual targeting of mTOR and HSP90 are being investigated [267].

### mTOR Inhibitors

Small molecules designed for inhibiting the catalytic site of mTOR have shown promising effects on suppression of signaling downstream of mTOR. mTOR kinase inhibitor have been developed which directly inhibit mTORC1 and mTORC2. The mTOR kinase inhibitors have advantages over rapamycin and rapalogs as the mTOR inhibitors will inhibit both mTORC1 and mTORC2 while rapamycin and rapalogs predominantly inhibit mTORC1. Also the mTOR kinases inhibitors do not induce the feedback pathways which result in Akt activation.

OSI-027 is a pan mTOR inhibitor developed by OSI Pharmaceuticals/Astellas Pharma Inc. OSI-027 is effective in inducing apoptosis in different types of cancer, including breast and leukemias [268,269]. OSI-027 has been shown to inhibit the growth of imatinib-resistant CML cells which contain the *BCR-ABL* T315I mutation that are resistant to all BCR-ABL inhibitors [270]. OSI-027 has been evaluated in a clinical trial (NCT00698243) with patients with advanced solid tumors and lymphoma [271].

PP-242 is a potent inhibitor of both mTORC1 and mTORC2 developed by Intellikine. INK-128 is a derivative of PP-242 which has shown anti-tumoral effects on multiple cancer types including RCC, MM, NHL and prostate neoplasia [272-274]. INK-128 is in phase I clinical trials (NCT01118689) for patients with relapsed or refractory MM or Waldenstrom macroglobulinemia or patients with solid malignancies (NCT01058707).

AZD8055 and AZD2014 are pan mTOR inhibitors with potent anti-tumor activity that have been developed by AstraZenica [275,276]. They are being evaluated in a clinical trial (NCT01316809) with individuals with gliomas who have not responded to standard glioma therapies as well as other types of cancer patients.

Palomid 529 (Paloma Pharmaceuticals) is a pan mTOR inhibitor which has potent anti-tumor affects and reduces tumor angiogenesis and vascular permeability [277]. Palomid 529 is undergoing phase I clinical trials for patients with macular degeneration (NCT01033721).

WAY600, WYE353, WYE687 and WYE132 were developed by Wyeth (Pfizer). These inhibitors were derived from WAY001 which was more specific for PI3K-alpha than either mTORC1 or mTORC2. These inhibitors were optimized which resulted in WYE132 (WYE125132)/ WYE132 has 5000-fold greater selectivity for mTOR over PI3K. It caused tumor regression in breast, glioma, lung, renal tumors [278].

Many other mTOR inhibitors have been described which include: Ku0063794 (KuDOS Pharmaceuticals) [279] and OXA-01 (OSI Pharmaceuticals) [280]. Torin2 has been developed by optimizing Torin1 [282]. TORKiCC223 is a pan mTOR inhibitor developed by Celgene. Other companies are developing mTOR inhibitors; clearly this is a very competitive but important research and clinical area.

Metformin is an indirect inhibitor of mTORC1. Metformin induces AMPK which turns on TSC1 which suppresses mTORC1 activity [8,282]. Metformin may also induce the phosphorylation and inactivation of Raptor [283]. Diabetics treated with metformin have lower incidences of cancer and also do not exhibit as much aging [284,285]. Metformin may be able to prevent the survival of certain CICs. Enhanced glycolysis (Warburg effect) is critical for CICs [286-289]. Metformin disrupts the glycolytic metabotype and alters the ATM-mediated DNA damage response resulting in the acceleration of stress-induced sencescence. Metformin in the presence of suppressed mTOR signaling slows down aging and alters the cellular senescence processes. Hence metformin can alter the ability of cells to become immortalized into CICs and slows down aging. By reducing the levels of DNA damage signaling, metformin has genoprotective affects [290,291].

A phase I clinical trial (NCT00659568) was performed on analyzing the effects of combining metformin with temsirolimus in patients with metastatic or unresectable solid tumor or lymphomas and demonstrated disease stabilization [292].

Inhibition of RHEB by farnesyltransferase (FT) inhibitors is another mechanism to inhibit mTORC1 [293]. FT inhibitors have been extensively examined in clinical trials [294].

### PP2A Activators

Successful targeting of the protein phosphatases has in general not proceeded as rapidly as targeting of protein kinases. FTY720 (fingolimod) is a PP2A activator which has been approved as an immunomodulator for oral use in patients with multiple sclerosis [295]. Reactivation of PP2A activity by FTY720 suppressed cell growth, enhanced apoptosis, impaired clonogenicity, and decreased *in vivo* leukemogenesis of imatinib- and dasatinib-sensitive and -resistant Ph^+^ B-ALL cells, as well as Ph^+^ B-ALL progenitors (CD34^+^/CD19^+^). Importantly, healthy CD34^+^ and CD34^+^/CD19^+^ bone marrow cells were unaffected by FTY720. Furthermore, pharmacologic doses of FTY720 suppressed *in vivo BCR-ABL*-driven leukemogenesis (including leukemogenesis promoted by the *BCR-ABL* T315I mutant which is resistant to imatinib and second generation TKIs) without exerting any toxicity in mice [296].

### Increasing the Effectiveness of Targeting the Raf/MEK/ERK and PI3K/PTEN/Akt/mTOR Pathways by Simultaneous Treatment with Two Pathway Inhibitors

The obvious goal of current inhibitor development is to improve the effectiveness of treatment of cancer patients with small molecule signal transduction inhibitors. This has proven to be difficult for multiple reasons: first, as previously discussed, there tends to be a distinct genetic susceptibility for the success of a signal transduction inhibitor in suppressing growth, second, many of the small molecule signal transduction inhibitors are cytostatic as opposed to being cytotoxic and therefore will need to be combined with a therapeutic modality that induces cell death, and third, more than one signal transduction pathway may be activated in the cancer cells, which will be discussed in detail below.

Previously, we have predominantly discussed studies that employed a single Raf or MEK inhibitor, sometimes in combination with a chemotherapeutic drug. In the following section, we discuss the potential of combining inhibitors that target two pathways to more effectively limit cancer growth. In addition to the *BRAF* mutations present in melanomas that we have previously discussed, the *PTEN* phosphatase tumor suppressor gene is also deleted in approximately 45% of melanomas and the downstream *AKT* gene is amplified in approximately 45%. Both of these mutations result in increased expression/activity of Akt which is often associated with a poor prognosis in human cancer. Increased Akt expression will lead to mTOR activation and increased efficiency of protein translation. Preclinical studies performed in human melanoma cell lines have highlighted that co-targeting of the Raf/MEK/ERK and PI3K/PTEN/Akt/mTOR pathways with Raf and Akt/mTOR inhibitors resulted in synergistic inhibition [297]. Treatment of inducible murine lung cancers containing *KRAS* and *PIK3CA* mutations with PI3K/mTOR (NVP-BEZ235) and MEK (selumetinib) inhibitors led to an enhanced response [298]. Synergistic responses between sorafenib and mTOR inhibitors were observed in xenograft studies with a highly metastatic human HCC tumor [299]. Some recent studies in thyroid cancer have documented the benefit of combining Raf and PI3K/mTOR inhibitors [300].

Intermittent dosing of MEK and PI3K inhibitors has been observed to suppress the growth of tumor xenografts in mice [87]. This study demonstrated that continuous administration of MEK and PI3K inhibitors is not required to suppress xenograft growth. These important results were obtained by performing washout studies *in vitro* and alternate dosing schedules in mice with MEK and PI3K inhibitors with *BRAF* and *KRAS* mutant cancer cells.

The combined effects of inhibiting MEK with PD-0329501 and mTOR with rapamycin or its analog AP-23573 (ARIAD Pharmaceuticals/Merck) were examined in human NSCLC cell lines, as well as in animal models of human lung cancer [301]. PD-0325901 and rapamycin demonstrated synergistic inhibition of proliferation and protein translation. Suppression of both MEK and mTOR inhibited ribosomal biogenesis and was associated with a block in the initiation phase of translation. The pan mTOR inhibitor AZD-8055 has been examined as a single agent and in combination with the MEK inhibitor AZD-6244 in a NSCLC xenograft model. The combination resulted in increased cell death and tumor regression [275,302]. These preclinical results support suppression of both the MEK and mTOR pathways in lung cancer therapy and indicate that both pathways converge to regulate the initiation of protein translation. ERK phosphorylates Mnk1/2 and p90^Rsk^, which regulate the activity of the eukaryotic translation initiation factor eIF4E. The phosphorylation of 4EBP1 is altered in cells with the *BRAF* mutation. It should also be pointed out that the 4EBP1 is also regulated by Akt, mTOR and p70S6K. This may result in the efficient translation of certain mRNAs in *BRAF-*mutant cells. This could explain how co-inhibition of MEK and mTOR synergize to inhibit protein translation and growth in certain lung cancer cells. mTOR inhibitors have been combined with HSP90 inhibitors to overcome resistance to rapamycin [267].

The effects of combining the MEK inhibitor RDEA119 and rapamycin have been examined in various cancers including pancreatic cancer [303]. The effects of dual inhibition of IGF-1R and mTOR have been examined in myeloma and other cancers [304]. Also the effectiveness of combination of rapalogs and EGFR inhibitors to inhibit glioblastoma growth is being examined [305]. The antiproliferative effects of the Akt inhibitor perifosine is improved when combined with nanoparticle-bound rapamycin on multiple myeloma cells [306].

Treatment of vemurafenib-resistant *BRAF*-mutant colorectal cancer cells with an Akt inhibitor (MK-2206) overcame their resistance to vemurafenib [307]. Heat shock inhibitors such as the HSP90 inhibitor XL888, have been shown to inhibit proliferation of some vemurafenib-resistant melanoma cells [308]. XL888 increased pro-apoptotic Bim expression and decreased Mcl-1 expression. Also decreases in PDGFR-beta, COT, IGF-1R, Raf-1, A-Raf, S6, cyclin D1 and Akt were observed. This lead to nuclear accumulation of FOXO3a and resulted in expression of the proapoptotic Bim protein.

### Clinical Trials Based upon Inhibiting both the Raf/MEK/ERK and PI3K/PTEN/Akt/mTOR Pathways

**Figure 5:**
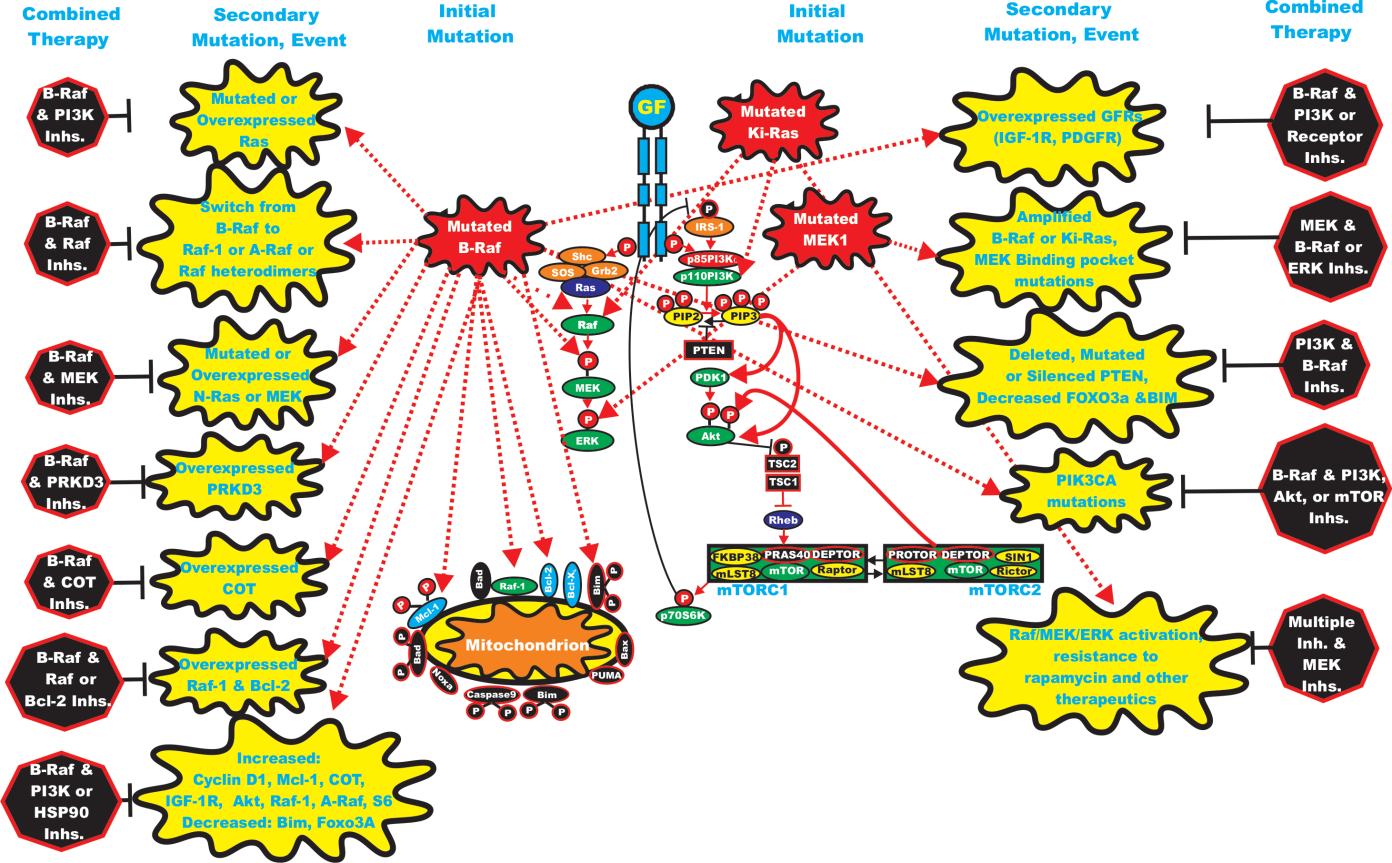
Rationale for Targeting Both the Ras/Raf/MEK/ERK and Ras/PI3K/PTEN/Akt/mTOR Pathways for Suppressing Inhibitor Resistant Cells Initial mutations are depicted in red irregular circle. Secondary mutation/events which result in inhibitor resistance are depicted in yellow irregular circle. Potential combination inhibitor therapeutic approaches are indicated in black octagons. The remaining color scenarios are as presented in Figures 1, 2, 3 and 4. Normal activating signaling is indicated in either solid red or solid black lines. Signaling induced by mutations is indicated by red dashed lines.

Combinations of Raf and PI3K/mTOR or MEK and PI3K/mTOR inhibitors are in clinical trials. The results of a phase 1 clinical trial on patients with advanced solid tumors indicate that the combined dosing appears to be well tolerated, at least as well as single agent dosing. Some anti-tumor effects were observed and dose-escalation trials were performed [309]. NCT01138085 is a clinical trial combining MEK and Akt inhibitors (GSK1120212 and GSK2141795 respectively). NCT01347866 is a clinical trial for patients with advanced cancers combining the PI3K/mTOR inhibitors (PF-04691502 & PF-05212384) with the MEK inhibitor (PD-0325901) or irinotecan. The study will include patients with metastatic CRC who have received previous therapy for their disease and whose cancers have a mutant *KRAS* gene. The dual PI3K/mTOR inhibitor NVP-BEZ235 is in a combination clinical trial (NCT01482156) with RAD001 (everolimus) in patients with advanced solid cancers. A phase 1 clinical trial (NCT01337765) is in progress combining the MEK1/2 inhibitor MEK162 and the PI3K/mTOR dual inhibitor NVP-BEZ-235. This combination will be evaluated in various cancer patients, for example in NSCLC patients containing mutations at *EGFR* who have progressed after treatment with EGFR inhibitors or with patients with triple negative breast, CRC, melanoma, and pancreatic cancers. In addition, patients with other advanced solid tumors with *KRAS*, *NRAS*, and/or *BRAF* mutations will be included in this trial. NCT01390818 is a research trial testing a combination of two experimental drugs, MSC1936369B (a MEK inhibitor) and SAR245409 (a PI3K/mTOR inhibitor), (EMD Serono and Sanofi) for the treatment of locally advanced or metastatic solid tumors. Patients with breast, NSCLC, melanoma and colorectal cancers will be treated with this inhibitor combination. A clinical trial NCT01021748 is examining the effects of combining MK2206 (Akt inhibitor) and AZD6244 (selumetinib, MEK inhibitor) in cancer patients with advanced solid tumors. NCT01519427 is a clinical trial combining the MEK inhibitor selumetinib and the Akt inhibitor MK2206 in patients with stage III or stage IV melanoma that previously failed after treatment with vemurafenib or dabrafenib. A diagram illustrating potential combined inhibitor therapy to overcome resistance is presented in Figure 5.

### Enhancing Effectiveness of Raf/MEK and PI3K/Akt/mTOR Inhibitors with Chemotherapy

Classical chemotherapy often remains the most prescribed anti-cancer therapy for many different types of cancer treatment. Optimizing chemotherapy with targeted therapy may require genetic analysis to obtain the best response which may also depend on the timing of individual drug treatment [310-320]. Drugs such as doxorubicin and taxol are effective in the treatment of many cancers, even though in some cases drug resistance develops after prolonged treatment. Doxorubicin, taxol and other chemotherapeutic drugs alter cellular events, such as DNA replication [321], DNA repair [322], cell division [323-325], polyploidy [326], autophagy [327,328], angiogenesis [329] or the tumor microenvironment [330]. Often the effects of the chemotherapeutic drug are dependent upon the *TP53* gene status [331-334].

Chemotherapeutic drugs can activate the Ras/Raf/MEK/ERK pathway by diverse mechanisms. Drugs such as doxorubicin can activate p53 which can lead to increased expression of the discoidin domain receptor (DDR), which in turn can result in Raf/MEK/ERK pathway activation. Activated ERK can phosphorylate p53 and regulate its activity. Doxorubicin can also activate the calcium calmodulin dependent kinase (CaM-K) cascade via ROS [4,335]. Activation of this cascade can also result in stimulation of the Raf/MEK/ERK cascade which induces the transcription of genes which are involved in DNA repair and lead to drug resistance [4,335]. Taxols can also stimulate activation of the Raf/MEK/ERK cascade and lead to their increased association with proteins involved in cell division [336,337] Thus, by combining classical chemotherapy with targeted therapy, it may be possible to enhance toxicity, while lowering the prescribed concentrations of classical chemotherapeutics necessary for effective elimination of the tumor [337]. Activation of the Raf/MEK/ERK cascade can alter the activity and subcellular localization of many proteins that play critical roles in apoptotic cascades. Also the Raf/MEK/ERK cascade can regulate the transcription of many critical genes involved in cell cycle progression, growth and differentiation [3,4].

The 5 year survival rate for CRC is less than 10%, thus novel therapies are required to improve treatment of this cancer. *KRAS* is often mutated in CRC, thus the Raf/MEK/ERK pathway will be activated. The effects of combining the MEK inhibitor selumetinib with vorinostat [a histone deacetylase (HDAC) inhibitor] were examined in a recent study [338]. Combining the two inhibitors resulted in a synergistic response *in vitro*, while an additive response was observed *in vivo*.

Treatment of mice xenografted with vemurafenib-resistant *BRAF*-mutant CRCs with various combinations of vermurafenib and chemotherapeutic drugs (capecitabine, irinotecan), monoclonal antibodies [bevacizumab a.k.a avastin, targets VEGF-alpha, Roche/Genentech), cetuximab (a.k.a erbitux, targets EGFR, Imclone/Eli Lilly)], or the small molecule Akt inhibitor MK-2206, or the EGFR inhibitor erlotinib increased survival [307]. Combination of the Akt inhibitor MK-2206 and either EGFR/HER2 targeted therapy [erlotinib (a.k.a tarceva, an EGFR inhibitor from Genenetec/OSI/Roche) or lapatinib (a.k.a. tykerb, a dual EGFR and HER2 inhibitor from GSK) or chemotherpapeutic drugs (doxorubicin, camptothechin, gemcitabine, 5-flurouracil, docetaxel or carboplatin resulted in synergistic responses in lung (NCI-H460) and ovarian (A2780) cancer cell lines. In some cases, the timing of drug addition was determined to be important as MK-2206 suppressed the Akt activation induced by carboplatin and gemcitabine [339]. The effects of combining the dual PI3K/mTOR inhibitor NVP-BEZ235 and various chemotherapeutic drugs as well as other targeted therapies are being examined (doxorubicin, melphalan, vincristine, bortezomib) [340,341]. The effects of the pan mTOR inhibitor INK-128 could be enhanced by the addition of sorafenib and avastin [272,273]. A clinical trial (NCT01351350) with INK-128 in combination with paclitaxel, either in the absence or presence of herceptin, is in progress in patients with advanced solid malignancies. The anti-tumor effects of the mTOR inhibitor WYE132 could be enhanced upon combination with avastin in lung and breast xenograft models [278].

Clinical trials are ongoing based on combining NVP-BEZ235 using inhibitors (BKM120, MEK162) and the chemotherapeutic drug (paclitaxel) and herceptin to treat advanced solid cancers and metastatic breast cancers which are difficult to treat (see below). BKM120 is a pan-PI3K inhibitor. It is being included in some clinical studies since NVP-BEZ235 does not inhibit PI3K-P110-β [242]. Furthermore NVP-BEZ235 is not effective in suppressing the growth of tumors which have the *KRAS G12D* mutation [343]. Thus to achieve effective suppression of cancer growth in some situations, it maybe be important to combine PI3K/mTOR inhibitors with pan PI3K inhibitors.

Palomid 529, a pan mTOR inhibitor, in some circumstances is effective as a single agent. Importantly when Palomid 529 was combined with either cisplatin or docetaxel it had a better effect on hormone-refractory prostate cancers [344]. It also improved the effects of radiotherapy on prostate cancer cells [345].

As mentioned previously, a side effect of some chemotherapeutic drugs, such as paclitaxel, is the induction of the Raf/MEK/ERK pathway. Activation of this pathway, can under certain circumstances, promote proliferation and prevent apoptosis. Also the PI3K/PTEN/Akt/mTOR pathway can modulate the Raf/MEK/ERK pathway and altering MEK activity can have opposing effects on different cell types [346-349]. Combining paclitaxel treatment with PI3K inhibitors enhances apoptosis and inhibits growth of ovarian carcinoma cell lines, and this may have been mediated in part by suppression of inhibitory phosphorylation of Raf by Akt [346]. In addition, the effects of combined treatment with MEK inhibitors and paclitaxel have been examined. The synergistic effects of paclitaxel and MEK inhibitors are complex and not fully elucidated, but may be in part mediated by inhibition of Bad phosphorylation at S112 by ERK in UM-SCC-23 squamous carcinoma cell line [348].

The cytotoxic effects of combinations of MEK inhibitors and paclitaxel may be specific for cells of certain origins and may depend on the levels of endogenous activated MEK/ERK present in those cells. In a study with NSCLC cells which constitutively-expressed activated MEK/ERK, no increase in paclitaxel-induced apoptosis was observed when the cells were treated with a MEK inhibitor [347]. In contrast, addition of a dominant negative (DN) MEK gene to these cells potentiated paclitaxel-induced apoptosis.

Cisplatin-induced apoptosis was associated with increased levels of both p53 and the downstream Bax protein in a study with neuroblastoma cells [348]. Activated ERK1/ERK2 levels also increased in these cells upon cisplatin treatment. MEK inhibitors blocked apoptotic cell death, which prevented the cisplatin-induced accumulation of p53 and Bax proteins [348].

It should be noted that the combination of MEK inhibitors and chemotherapeutic drugs may not always result in a synergistic interaction leading to cell death. In some cases, combination therapy results in an antagonistic response. For example, combining MEK inhibitors with betulinic acid, a drug toxic for melanoma cells, antagonized the normal enhancing effects of betulinic acid on apoptosis *in vitro* [349]. Furthermore, the precise timing of the addition of two agents is important as they may differentially affect cell-cycle progression; therefore, the order of administration may be important for a synergistic response to be obtained and perhaps to prevent an antagonistic response.

There are few effect therapeutic options for HCC. Combination of rapamycin with conventional cytostatic drugs such as doxorubicin and vinblastine enhances the antineoplastic activity of the respective monotherapeutic HCC treatment obtained with either doxorubicin or vinblastine alone [350,351]. Taken together, the *in vitro* and preclinical *in vivo* data as well as the clinical trials conducted so far demonstrate that mTOR inhibitors are promising agents for HCC treatment, particularly in combination with conventional chemotherapeutic drug therapy.

The effects of sorafenib on the treatment of HCC patients were examined in a clinical trial [350]. A phase II trial demonstrated that the combination of sorafenib and doxorubicin improved progression-free and overall survival of patients with advanced HCC [351]. Moreover, a phase II trial (NCT00464919) was performed to determine the progression-free survival of sorafenib plus tegafur/uracil (UFUR) for the treatment of advanced or metastatic HCC. The study indicated that UFUR can be safely combined with sorafenib and may improve the efficacy of sorafenib in advanced HCC patients [352].

The effects of inhibiting Akt in combination with other signaling pathways and chemotherapy are being evaluated in numerous phase I clinical trials. These trials highlight the importance of targeting multiple molecules to suppress the growth of cancer which are resistant to most therapies. A combination clinical trial (NCT01245205) with the Akt inhibitor MK-2206 and the dual EGFR/HER2 inhibitor lapatinib is in progress with patients having advanced or metastatic solid tumors or breast cancer patients. NCT00848718 is a clinical trial with patients having advanced cancers to examine the effects of combining MK-2206 and the EGFR inhibitor erlotinib, docetaxel, or carboplatin + paclitaxel. NCT00963547 was a clinical trial with HER2+ breast cancer patients to examine the effects of combining MK2206 with trastuzumab (herceptin) and lapatinib. NCT01245205 and NCT01281163 are clinical trials examining the effects of combining MK2206 with lapatinib in cancer patients with advanced or metastatic solid tumors or breast cancer or just breast cancers, respectively. NCT01147211 is a clinical trial with NSCLC patients examining the effects of combining MK-2206 with gefitinib (a.k.a. Iressa, EGFR inhibitor developed by AstraZenica). NCT01344031 is a clinical trial with post menopausal metastatic breast cancer patients examining the effects of combining anastrozole, letrozole, exemestane (aromatase-inhibitors), or fulvestrant (an estrogen receptor antagonist). NCT01369849 is a clinical trial examining the effects of combining MK2206, with bendamustin (nitrogen mustard alkylating agent) and rituximab (a.k.a. Rituxan, a chimeric monoclonal antibody targeting CD20 from IDEC Pharmaceuticals/Genenetec) on CLL cancer patients who have relapsed or cancer patients with small lymphocytic lymphoma. NCT01243762 is a clinical trial combining MK-2206 and dalotuzumab (monoclonal Ab targeting IGF-1R from Merck), MK-0752 a (Y-secretase inhibitor which inhibits the NOTCH pathway from Merck) and dalotuzumab and MK-8669 (ridaforolimus a mTOR inhibitor from Merck) and dalotuzumab in cancer patients with advanced cancers. NCT01263145 is a clinical trial combining MK2206 and paclitaxel in cancer patients with locally advanced or metastatic solid tumors or metastatic breast cancers. The above mentioned clinical trials document the importance of targeting Akt and other signaling molecules as well as critical targets involved in cellular division. Furthermore the clinical trials document how basis research experimentation on these pathways is being translated into clinical therapy for cancer and other types of patients.

### Enhancing Effectiveness of Raf/MEK and PI3K/mTOR Inhibitors with Radiotherapy

Radiotherapy is a common therapeutic approach for treatment of many diverse cancers [353]. Radiotherapy often induces DNA double strand breaks [354]. The successfulness of radiotherapy is often governed by the functionality of p53 and its affects on apoptosis [355,356]. The ability to improve the effects of radiotherapy with small molecule inhibitors is an area of active research interest [357].

A side effect of radiotherapy in some cells is induction of the Ras/Raf/MEK/ERK cascade [3,4]. Various signal transduction inhibitors have been evaluated as radiosensitizers. The effects of pre-treatment of lung, pancreatic and prostate cancer cells with selumetinib were evaluated *in vitro* using human cell lines and *in vivo* employing xenografts [358]. The MEK inhibitor treatment radiosensitized various cancer cell lines *in vitro* and *in vivo*. The MEK inhibitor treatment was correlated with decreased Chk1 phosphorylation 1-2 hrs after radiation. The authors noticed the effects of the MEK inhibitor on the G_2_ checkpoint activation after irradiation, as the MEK inhibitor suppressed G_2_ checkpoint activation. Since ERK1/ERK2 activity is necessary for carcinoma cells to arrest at the G_2_ checkpoint, suppression of phosphorylated Chk1 was speculated to lead to the abrogated G_2_ checkpoint, increased mitotic catastrophe and impaired activation of cell cycle checkpoints. Chk1/Chk2 as serine/threonine kinases. Chk/Chk2 are important controlling regulators of DNA repair and cell cycle progression. DNA damage responses which signal through ATM and ATR activate the DNA damage transducers Chk1 and Chk2 [359-383].

Mitotic catastrophe was increased in cancer cells receiving both the MEK inhibitor selumetinib and radiation when compared to the solo-treated cells [358]. Suppression of MEK activity resulted in decreased phosphorylated Chk1 leading to the abrogated G_2_ checkpoint. It was also postulated in this study that the MEK inhibitor suppressed the autocrine cascade in DU145 prostate cancer cells that normally resulted from EGF secretion and EGFR activation. Suppression of this autocrine cascade by the MEK inhibitor may have served as a radiosensitizer to the radiation therapy. The other two cancer cell lines examined in this study (A549 and MiaPaCa2) had *KRAS* mutations and both were radiosensitized by the MEK inhibitor. Although these studies document the ability of a MEK inhibitor to radiosensitize certain cells, clearly other cancer cell lines without activating mutations in the Ras/Raf/MEK/ERK pathway or autocrine growth stimulation should be examined for radiosensitization by the MEK inhibitor as the *KRAS* mutation may also activate the PI3K pathway which could lead to therapy resistance.

PI3K/Akt/mTOR inhibitors will sensitize the tumor vasculature to radiation both *in vitro* in cell lines and *in vivo* in xenografts [384,385]. mTOR and radiation play critical roles in the regulation of autophagy [386,387]. These studies document the potential beneficial use of combining mTOR inhibitors and radiation to improve the induction of autophagy in the treatment of solid tumors. This is important as apoptotic cell death is a minor component to cell death in solid tumors. When mTOR is blocked by rapamycin there is an increase in autophagy [388-393]. mTORC1 is a repressor of autophagy, a lysosome-dependent degradation pathway which allows cells to recycle damaged or superfluous cytoplasmic content, such as lipids, proteins, and organelles. As a consequence, cells produce metabolic precursors for macromolecular biosynthesis or ATP generation [392]. In cancer cells, autophagy fulfils a dual role, as it has both tumor-promoting and tumor-suppressing properties. Autophagy is also an important component in hematopoietic cancers and some therapy-resistant cells have defects in autophagy [394-396] Functional autophagy prevents necrosis and inflammation, which can lead to genetic instability. However, autophagy might be important for tumor progression by providing energy through its recycling mechanism during unfavorable metabolic circumstances, which are very common in tumors [397-399].

## CONCLUSIONS

Inhibitors to the Ras/Raf/MEK/ERK and Ras/PI3K/PTEN/Akt/mTOR pathways have been isolated and developed by various screening approaches and then in some cases modified by medicinal chemistry. Initially MEK and mTOR inhibitors were demonstrated to have the most specificity. However, MEK inhibitors may have limited effectiveness in treating human cancers, unless the particular cancer proliferates directly in response to the Raf/MEK/ERK pathway. A similar scenario is also true with mTOR inhibitors, they are most effective when there is a mutation which deregulates the PI3K/PTEN/Akt/mTOR pathways. Moreover, MEK inhibitors are often cytostatic as opposed to cytotoxic, thus their ability to function as effective anti-cancer agents in a monotherapeutic setting is limited, and they may be more effective when combined with chemo- or radiotherapy or an inhibitor which targets a different pathway or even an inhibitor which targets the same pathway. Rapamycin and rapalogs are being used to treat certain cancers which proliferate in response to mutations in regulatory genes which control the PI3K/PTEN/Akt/mTOR pathway. Raf inhibitors have also been developed and some are being used to treat various cancer patients (*e.g.,* sorafenib, HCC). This particular Raf inhibitor also inhibits other receptors and kinases which may be required for the growth of the particular cancer. This promiscuous nature of sorafenib has contributed to the effectiveness of this particular Raf inhibitor for certain cancers. Raf inhibitors such as vemurafenib, dabrafenib, and GDC-0879 are promising for the treatment of melanoma, CRC, thyroid and other solid cancers and leukemias/lymphomas/myelomas which have mutations at *BRAF* V600E. However, problems have been identified with certain *BRAF* mutant allele inhibitors as they will also result in Raf-1 activation if *RAS* is mutated/amplified of if an exon of *BRAF* is deleted, or if *BRAF* is amplified or if there are mutations at *MEK1* and other genetic mechanisms. Combination therapy with either a traditional drug/physical treatment or another inhibitor that targets a specific molecule in a different signal transduction pathway is also a key approach for improving the effectiveness and usefulness of MEK and Raf inhibitors.

Modified rapamycins, rapalogs are being used to treat various cancer patients, (*e.g.,* patients with RCC). While rapalogs are effective and their toxicity profiles are well known, one inherent property is that they are not very cytotoxic when it comes to killing tumor cells. This inherent property of rapamycins, may also contribute to their low toxicity in humans. Interestingly and highly relevant, it has been observed that certain inhibitors which target “growth and metabolism” such as rapamycin and metformin may have very potent anti-cancer and anti-aging effects [233-239, 284-291]

Mutations at many of the upstream receptor genes or *RAS* can result in abnormal Raf/MEK/ERK and PI3K/PTEN/Akt/mTOR pathway activation. Hence targeting these cascade components with small-molecule inhibitors may inhibit cell growth. The usefulness of these inhibitors may depend on the mechanism of transformation of the particular cancer. If the tumor exhibits a dependency on the Ras/Raf/MEK/ERK pathway, then it may be sensitive to Raf and MEK inhibitors. In contrast, tumors that do not display enhanced expression of the Ras/Raf/MEK/ERK pathway may not be sensitive to either Raf or MEK inhibitors but if the Ras/PI3K/Akt/mTOR pathway is activated, it may be sensitive to specific inhibitors that target this pathway. Some promising recent observations indicate that certain CICs may be sensitive to mTOR inhibitors [3,4.^6^,^7^,^195^,^196^,^216^,^400^-^404^] and metformin [8,129,269,286,287,405,406], documenting their potential use in the elimination of the cells responsible for cancer re-emergence. Finally, it is likely that many of the inhibitors that we have discussed in this review will be more effective in inhibiting tumor growth in combination with cytotoxic chemotherapeutic drugs or radiation.

Some scientists and clinicians have considered that the simultaneous targeting of Raf and MEK by individual inhibitors may be more effective in cancer therapy than just targeting Raf or MEK by themselves. This is based in part on the fact that there are intricate feed-back loops from ERK which can inhibit Raf and MEK. For example when MEK1 is targeted, ERK1,2 is inhibited and the negative feed-back loop on MEK is broken and activated MEK accumulates. However, if Raf is also inhibited, it may be possible to completely shut down the pathway. This is a rationale for treatment with either dual Raf/MEK inhibitors or simultaneously with both Raf and MEK individual inhibitors. Likewise targeting both PI3K and mTOR may be more effective than targeting either PI3K or mTOR by themselves. If it is a single inhibitor which targets both molecules, such as the new PI3K and mTOR dual inhibitors this becomes a realistic therapeutic option. Also in some cases it may be necessary to eliminate the cancer by treatment with a dual PI3K/mTOR inhibitor as well as with an additional PI3K inhibitor which suppresses the PI3K-p110-delta isoform as certain dual PI3K/mTOR inhibitors do not effectively suppress this isoform. Finally, an emerging concept is the dual targeting of two different signal transduction pathways, Raf/MEK/ERK and PI3K/PTEN/Akt/mTOR for example. This has been explored in some preclinical models as well as clinical trials as discussed in the text. The rationale for the targeting of both pathways may be dependent on the presence of mutations in either/or both pathways or in upstream Ras in the particular cancer which can activate both pathways.

It is not always clear why a particular combination of a signal transduction inhibitor and chemotherapeutic drug works in one tumor type but not at all in a different tumor type. This has also been experience with the development of individual chemotherapeutic drugs, some work in some cancers but not others. This may result from many different complex interacting events. Some of these events could include: percentage of cells in different phases of the cell cycle, persistence of CICs, presence of multiple mutated activated oncogene or repressed tumor suppressor genes, epigenetic modifications and many other factors. Finally, chemotherapeutic drug therapy and other types of therapy (radiotherapy, antibody therapy) may induce certain signalling pathways (*e.g*., the ROS generated by chemotherapy and radiotherapy induce the Ras/Raf/MEK/ERK pathway). The induction of these signaling pathways may counteract some of the effects of the signal transduction inhibitors.

A problem with some of the preceding studies is that most of the resistant cells were derived after culturing cells *in vitro* for prolonged periods of time in the presence of increasing doses of B-Raf inhibitors. The clinical relevance of these mechanisms of resistance awaits their identification in resistant samples from melanoma and other cancer patients treated with these inhibitors. Furthermore, many of the studies were performed on different established melanoma cell lines which have various additional mutations besides those in *BRAF* that may or may not be relevant for actual melanomas present in patients. Finally the various melanoma cell lines may be at different stages of differentiation and thus the genes involved in resistance *in vitro*, may be different from what is observed in other classes (stages of differentiation) of melanoma *in vivo*. Interesting, increased drug transporter activity has not been reported in the limited number of B-Raf inhibitor-resistant samples investigated, where it has been observed in other cancer types treated with diverse small molecule inhibitors and/or chemotherapeutic drugs.

Scientists and clinicians often have an intentionally narrow view of a particular topic. For example, cancer researchers predominantly feel that Raf, MEK, PI3K, Akt and mTOR inhibitors will suppress the growth of malignant cancer cells. Yet MEK and mTOR and other inhibitors may also be useful in the treatment of autoimmune or allergic disorders where there is abnormal cellular proliferation. Recently it has been observed that the suppression of the Ras/Raf/MEK/ERK and Ras/PI3K/Akt/mTOR pathways may prevent the induction of cellular senescence and aging. Clearly, these later two clinical topics, immune disorders and aging, greatly enhance the potential clinical uses of these targeted therapeutic drugs.

## References

[1] Barretina J, Caponigro G, Stransky N, Venkatesan K, Margolin AA, Kim S, Wilson CJ, Lehár J, Kryukov GV, Sonkin D, Reddy A, Liu M, Murray L, Berger MF, Monahan JE, Morais P (2012). The Cancer Cell Line Encyclopedia enables predictive modelling of anticancer drug sensitivity. Nature.

[2] Garnett MJ, Edelman EJ, Heidorn SJ, Greenman CD, Dastur A, Lau KW, Greninger P, Thompson IR, Luo X, Soares J, Liu Q, Iorio F, Surdez D, Chen L, Milano RJ, Bignell GR (2012). Systematic identification of genomic markers of drug sensitivity in cancer cells. Nature.

[3] Chappell WH, Steelman LS, Long JM, Kempf RC, Abrams SL, Franklin RA, Bäsecke J, Stivala F, Donia M, Fagone P, Malaponte G, Mazzarino MC, Nicoletti F, Libra M, Maksimovic-Ivanic D, Mijatovic S (2011). Ras/Raf/MEK/ERK and PI3K/PTEN/Akt/mTOR Inhibitors: Rationale and Importance to Inhibiting These Pathways in Human Health. Oncotarget.

[4] McCubrey JA, Steelman LS, Kempf CR, Chappell W, Abrams SL, Stivala F, Malaponte G, Nicoletti F, Libra M, Bäsecke J, Maksimovic-Ivanic D, Mijatovic S, Montalto G, Cervello M, Cocco L, Martelli AM (2011). Therapeutic resistance resulting from mutations in Raf/MEK/ERK and PI3K/PTEN/Akt/mTOR signaling pathways. J Cell Physiol.

[5] Solit DB, Garraway LA, Pratilas CA, Sawai A, Getz G, Basso A, Ye Q, Lobo JM, She Y, Osman I, Golub TR, Sebolt-Leopold J, Sellers WR, Rosen N (2006). BRAF mutation predicts sensitivity to MEK inhibition. Nature.

[6] Martelli AM, Evangelisti C, Chappell W, Abrams SL, Bäsecke J, Stivala F, Donia M, Fagone P, Nicoletti F, Libra M, Ruvolo V, Ruvolo P, Kempf CR, Steelman LS, McCubrey JA (2011). Targeting the translational apparatus to improve leukemia therapy: roles of the PI3K/PTEN/Akt/mTOR pathway. Leukemia.

[7] Martelli AM, Chiarini F, Evangelisti C, Cappellini A, Buontempo F, Bressanin D, Fini M, McCubrey JA (2012). Dual Inhibitors of phosphatidylinositol 3-kinase and mammalian target of rapamycin: a novel therapeutic strategy for acute leukemia treatment? Oncotarget.

[8] Martelli AM, Chiarini F, Evangelisti C, Ognibene A, Bressanin D, Billi AM, Manzoli L, Cappellini A, McCubrey JA (2012). Targeting the liver kinase B1/AMP-dependent kinase pathway as a therapeutic strategy for hematological malignancies. Expert Opinion Therapeutic Targets.

[9] McCubrey JA, Steelman LS, Abrams SL, Chappell WH, Russo S, Ove R, Milella M, Tafuri A, Lunghi P, Bonati A, Stivala F, Nicoletti F, Libra M, Martelli AM, Montalto G, Cervello M (2010). Emerging MEK inhibitors. Exp Opin Emerging Drugs.

[10] Steelman LS, Chappell WH, Abrams SL, Kempf RC, Long J, Laidler P, Mijatovic S, Maksimovic-Ivanic D, Stivala F, Mazzarino MC, Donia M, Fagone P, Malaponte G, Nicoletti F, Libra M, Milella M (2011). Roles of the Raf/MEK/ERK and PI3K/PTEN/Akt/mTOR pathways in controlling growth and sensitivity to therapy-implications for cancer and aging. Aging.

[11] Cervello M, McCubrey JA, Cusimano A, Lampiasi N, Azzolina A, Montalto G (2012). Targeted therapy for hepatocellular carcinoma: novel agents on the horizon. Oncotarget.

[12] Rimassa L, Santoro A (2009). Sorafenib therapy in advanced hepatocellular carcinoma: the SHARP trial. Expert Rev Anticancer Ther.

[13] Huynh H, Soo KC, Chow PK, Tran E (2007). Targeted inhibition of the extracellular signal-regulated kinases kinase pathway with AZD-6244 (ARRY-142886) in the treatment of hepatocellular carcinoma. Mol Cancer Ther.

[14] Poulikakos PI, Solit DB (2011). Resistance to MEK inhibitors: should we co-target upstream?. Sci Signal.

[15] Wilhelm SM, Carter C, Tang LY, Wilkie D, McNabola A, Rong H, Chen C, Zhang X, Vincent P, McHugh M, Cao Y, Shujath J, Gawlak S, Eveleigh D, Rowley B, Liu L (2004). BAY 43-9006 Exhibits broad spectrum oral antitumor activity and targets the RAF/MEK/ERK pathway and receptor tyrosine kinases involved in tumor progression and angiogenesis. Cancer Research.

[16] Mulder K, Koski S, Scarfe A, Chu Q, King K, Spratlin J (2010). Antiangiogenic agents in advanced gastrointestinal malignancies: past, present and a novel future. Oncotarget.

[17] Cervello M, Bachvarov D, Lampiasi N, Cusimano A, Azzolina A, McCubrey JA, Montalto G (2012). Molecular mechanisms of sorafenib action in liver cancer cells. Cell Cycle.

[18] Smalley KS, Xiao M, Villanueva J, Nguyen TK, Flaherty KT, Letrero R, Van Belle P, Elder DE, Wang Y, Nathanson KL, Herlyn M (2009). CRAF inhibition induces apoptosis in melanoma cells with non-V600E BRAF mutations. Oncogene.

[19] Flaherty KT, Puzanov I, Kim KB, Ribas A, McArthur GA, Sosman JA, O'Dwyer PJ, Lee RJ, Grippo JF, Nolop K, Chapman PB (2010). Inhibition of mutated, activated BRAF in metastatic melanoma. N Engl J Med.

[20] Chapman PB, Hauschild A, Robert C, Haanen JB, Ascierto P, Larkin J, Dummer R, Garbe C, Testori A, Maio M, Hogg D, Lorigan P, Lebbe C, Jouary T, Schadendorf D, Ribas A, BRIM-3 Study Group (2011). Improved survival with vemurafenib in melanoma with BRAF V600E mutation. N Engl J Med.

[21] Sambade MJ, Peters EC, Thomas NE, Kaufmann WK, Kimple RJ, Shields JM (2011). Melanoma cells show a heterogeneous range of sensitivity to ionizing radiation and are radiosensitized by inhibition of B-RAF with PLX-4032. Radiother Oncol.

[22] Bollag G, Hirth P, Tsai J, Zhang J, Ibrahim PN, Cho H, Spevak W, Zhang C, Zhang Y, Habets G, Burton EA, Wong B, Tsang G, West BL, Powell B, Shellooe R (2010). Clinical efficacy of a RAF inhibitor needs broad target blockade in BRAF-mutant melanoma. Nature.

[23] Tiacci E, Trifonov V, Schiavoni G, Holmes A, Kern W, Martelli MP, Pucciarini A, Bigerna B, Pacini R, Wells VA, Sportoletti P, Pettirossi V, Mannucci R, Elliott O, Liso A, Ambrosetti A (2011). “BRAF mutations in hairy-cell leukemia”. N Engl J Med.

[24] McCubrey JA, Steelman LS, Chappell WH, Abrams SL, Franklin RA, Montalto G, Cervello M, Nicoletti F, Fagone P, Malaponte G, Mazzarino MC, Candido S, Libra M, Bäsecke J, Milella M, Tafuri A (2012). Mutations and deregulation of Ras/Raf/MEK/ERK and PI3K/PTEN/Akt/mTOR cascades which alter therapy response. Oncotarget.

[25] Tsai J, Lee JT, Wang W, Zhang J, Cho H, Mamo S, Bremer R, Gillette S, Kong J, Haass NK, Sproesser K, Li L, Smalley KS, Fong D, Zhu YL, Marimuthu A (2008). Discovery of a selective inhibitor of oncogenic B-Raf kinase with potent antimelanoma activity. Proc Natl Acad Sci USA.

[26] Whittaker S, Kirk R, Hayward R, Zambon A, Viros A, Cantarino N, Affolter A, Nourry A, Niculescu-Duvaz D, Springer C, Marais R (2010). Gatekeeper mutations mediate resistance to BRAF-targeted therapies. Sci Transl Med.

[27] Falchook GS, Long GV, Kurzrock R, Kim KB, Arkenau TH, Brown MP, Hamid O, Infante JR, Millward M, Pavlick AC, O'Day SJ, Blackman SC, Curtis CM, Lebowitz P, Ma B, Ouellet D (2012). Dabrafenib in patients with melanoma, untreated brain metastases, and other solid tumours: a phase 1 dose-escalation trial. Lancet.

[28] Whittaker S, Ménard D, Kirk R, Ogilvie L, Hedley D, Zambon A, Lopes F, Preece N, Manne H, Rana S, Lambros M, Reis-Filho JS, Marais R, Springer CJ (2010). A novel, selective, and efficacious nanomolar pyridopyrazinone inhibitor of V600EBRAF. Cancer Res.

[29] Hoeflich KP, Herter S, Tien J, Wong L, Berry L, Chan J, O'Brien C, Modrusan Z, Seshagiri S, Lackner M, Stern H, Choo E, Murray L, Friedman LS, Belvin M (2009). Antitumor Efficacy of the Novel RAF Inhibitor GDC-0879 Is Predicted by BRAFV600E Mutational Status and Sustained Extracellular Signal-Regulated Kinase/Mitogen-Activated Protein Kinase Pathway Suppression. Cancer Research.

[30] Buchholz B, Klanke B, Schley G, Bollag G, Tsai J, Kroening S, Yoshihara D, Wallace DP, Kraenzlin B, Gretz N, Hirth P, Eckardt KU, Bernhardt WM (2011). The Raf kinase inhibitor PLX5568 slows cyst proliferation in rat polycystic kidney disease but promotes renal and hepatic fibrosis. Nephrol Dial Transplant.

[31] Zitzmann K, de Toni E, von Rüden J, Brand S, Göke B, Laubender RP, Auernhammer CJ (2011). The novel Raf inhibitor Raf265 decreases Bcl-2 levels and confers TRAIL-sensitivity to neuroendocrine tumour cells. Endocr Relat Cancer.

[32] Wilhelm SM, Dumas J, Adnane L, Lynch M, Carter CA, Schütz G, Thierauch KH, Zopf D (2011). Regorafenib (BAY 73-4506): a new oral multikinase inhibitor of angiogenic, stromal and oncogenic receptor tyrosine kinases with potent preclinical antitumor activity. Int J Cancer.

[33] Montagut C, Sharma SV, Shioda T, McDermott U, Ulman M, Ulkus LE, Dias-Santagata D, Stubbs H, Lee DY, Singh A, Drew L, Haber DA, Settleman J (2008). Elevated CRAF as a potential mechanism of acquired resistance to BRAF inhibition in melanoma. Cancer Res.

[34] Schwartz GK, Robertson S, Shen A, Wang E, Pace L, Dials H, Mendelson D, Shannon P, Gordon M (2009). A phase I study of XL281, a selective oral RAF kinase inhibitor, in patients (Pts) with advanced solid tumors. J Clin Oncol.

[35] Ratain MJ, Eisen T, Stadler WM, Flaherty KT, Kaye SB, Rosner GL, Gore M, Desai AA, Patnaik A, Xiong HQ, Rowinsky E, Abbruzzese JL, Xia C, Simantov R, Schwartz B, O'Dwyer PJ (2006). Phase II placebo-controlled randomized discontinuation trial of sorafenib in patients with metastatic renal cell carcinoma. J. Clin. Oncol.

[36] Eisen T, Ahmad T, Flaherty KT, Gore M, Kaye S, Marais R, Gibbens I, Hackett S, James M, Schuchter LM, Nathanson KL, Xia C, Simantov R, Schwartz B, Poulin-Costello M, O'Dwyer PJ (2006). Sorafenib in advanced melanoma: a Phase II randomised discontinuation trial analysis. Br J Cancer.

[37] Escudier B, Eisen T, Stadler WM, Szczylik C, Oudard S, Siebels M, Negrier S, Chevreau C, Solska E, Desai AA, Rolland F, Demkow T, Hutson TE, Gore M, Freeman S, Schwartz B, TARGET Study Group (2007). Sorafenib in advanced clear-cell renal-cell carcinoma. N Engl J Med.

[38] Takimoto CH, Awada A (2008). Safety and anti-tumor activity of sorafenib (Nexavar) in combination with other anti-cancer agents: a review of clinical trials. Cancer Chemother Pharmacol.

[39] Sharma A, Tran MA, Liang S, Sharma AK, Amin S, Smith CD, Dong C, Robertson GP (2006). Targeting mitogen-activated protein kinase/extracellular signal-regulated kinase kinase in the mutant (V600E) B-Raf signaling cascade effectively inhibits melanoma lung metastases. Cancer Res.

[40] Chapman PB, Hauschild A, Robert C, Haanen JB, Ascierto P, Larkin J, Dummer R, Garbe C, Testori A, Maio M, Hogg D, Lorigan P, Lebbe C, Jouary T, Schadendorf D, Ribas A, BRIM-3 Study Group (2011). Improved survival with vemurafenib in melanoma with BRAF V600E mutation. N Engl J Med.

[41] Sosman JA, Kim KB, Schuchter L, Gonzalez R, Pavlick AC, Weber JS, McArthur GA, Hutson TE, Moschos SJ, Flaherty KT, Hersey P, Kefford R, Lawrence D, Puzanov I, Lewis KD, Amaravadi RK (2012). Survival in BRAF V600-mutant advanced melanoma treated with vemurafenib. N Engl J Med.

[42] Kefford R, Arkenau H, Brown MP, Millward M, Infante JR, Long GV, Ouellet D, Curtis M, Lebowitz PF, Falchook GS (2010). Phase I/II study of GSK2118436, a selective inhibitor of oncogenic mutant BRAF kinase, in patients with metastatic melanoma and other solid tumors. J Clin Oncol.

[43] Long GV, Kefford RF, Carr PJA, Brown MP, Curtis M, Ma B, Lebowitz P, Kim KB, Kurzrock R, Flachook G (2010). Phase 1/2 study of GSK2118436, a selective inhibitor of V600 mutant (mut) BRAF kinase: evidence of activity in melanoma brain metastases (mets). Annals of Oncology.

[44] Poulikakos PI, Zhang C, Bollag G, Shokat KM, Rosen N (2010). RAF inhibitors transactivate RAF dimers and ERK signalling in cells with wild-type BRAF. Nature.

[45] Poulikakos PI, Rosen N (2011). Mutant BRAF melanomas--dependence and resistance. Cancer Cell.

[46] Joseph EW, Pratilas CA, Poulikakos PI, Tadi M, Wang W, Taylor BS, Halilovic E, Persaud Y, Xing F, Viale A, Tsai J, Chapman PB, Bollag G, Solit DB, Rosen N (2010). The RAF inhibitor PLX4032 inhibits ERK signaling and tumor cell proliferation in a V600E BRAF-selective manner. Proc Natl Acad Sci U S A.

[47] Heidorn SJ, Milagre C, Whittaker S, Nourry A, Niculescu-Duvas I, Dhomen N, Hussain J, Reis-Filho JS, Springer CJ, Pritchard C, Marais R (2010). Kinase-dead BRAF and oncogenic RAS cooperate to drive tumor progression through CRAF. Cell.

[48] Su F, Viros A, Milagre C, Trunzer K, Bollag G, Spleiss O, Reis-Filho JS, Kong X, Koya RC, Flaherty KT, Chapman PB, Kim MJ, Hayward R, Martin M, Yang H, Wang Q (2012). RAS mutations in cutaneous squamous-cell carcinomas in patients treated with BRAF inhibitors. N Engl J Med.

[49] Oberholzer PA, Kee D, Dziunycz P, Sucker A, Kamsukom N, Jones R, Roden C, Chalk CJ, Ardlie K, Palescandolo E, Piris A, MacConaill LE, Robert C, Hofbauer GF, McArthur GA, Schadendorf D (2012). RAS mutations are associated with the development of cutaneous squamous cell tumors in patients treated with RAF inhibitors. J Clin Oncol.

[50] Wagle N, Emery C, Berger MF, Davis MJ, Sawyer A, Pochanard P, Kehoe SM, Johannessen CM, Macconaill LE, Hahn WC, Meyerson M, Garraway LA (2011). Dissecting therapeutic resistance to RAF inhibition in melanoma by tumor genomic profiling. J Clin Oncol.

[51] Schmidt P, Abken H (2011). The beating heart of melanomas: a minor subset of cancer cells sustains tumor growth. Oncotarget.

[52] Schlaak M, Schmidt P, Bangard C, Kurschat P, Mauch C, Abken H (2012). Regression of metastatic melanoma in a patient by antibody targeting of cancer stem cells. Oncotarget.

[53] Caputo E, Maiorana L, Vasta V, Pezzino FM, Sunkara S, Wynne K, Elia G, Marincola FM, McCubrey JA, Libra M, Travali S, Kane M (2011). Characterization of human melanoma cell lines and melanocytes by proteome analysis. Cell Cycle.

[54] Bao W, Chen M, Zhao X, Kumar R, Spinnler C, Thullberg M, Issaeva N, Selivanova G, Stromblad S (2011). PRIMA-1Met/APR-246 induces wild-type p53-dependent suppression of malignant melanoma tumor growth in 3D culture and in vivo. Cell Cycle.

[55] Koomen JM, Smalley KS (2011). Using quantitative proteomic analysis to understand genotype specific intrinsic drug resistance in melanoma. Oncotarget.

[56] Chomel JC, Turhan AG (2011). Chronic myeloid leukemia stem cells in the era of targeted therapies: resistance, persistence and long-term dormancy. Oncotarget.

[57] Jagani Z, Dorsch M, Warmuth M (2010). Hedgehog pathway activation in chronic myeloid leukemia. Cell Cycle.

[58] Hochhaus A, La Rosee P, Muller MC, Ernst T, Cross NC (2011). Impact of BCR-ABL mutations on patients with chronic myeloid leukemia. Cell Cycle.

[59] Whittaker S, Kirk R, Hayward R, Zambon A, Viros A, Cantarino N, Affolter A, Nourry A, Niculescu-Duvaz D, Springer C, Marais R (2010). Gatekeeper mutations mediate resistance to BRAF-targeted therapies. Sci Transl Med.

[60] Nazarian R, Shi H, Wang Q, Kong X, Koya RC, Lee H, Chen Z, Lee MK, Attar N, Sazegar H, Chodon T, Nelson SF, McArthur G, Sosman JA, Ribas A, Lo RS (2010). Melanomas acquire resistance to B-RAF(V600E) inhibition by RTK or N-RAS upregulation. Nature.

[61] Poulikakos PI, Persaud Y, Janakiraman M, Kong X, Ng C, Moriceau G, Shi H, Atefi M, Titz B, Gabay MT, Salton M, Dahlman KB, Tadi M, Wargo JA, Flaherty KT, Kelley MC (2011). RAF inhibitor resistance is mediated by dimerization of aberrantly spliced BRAF(V600E). Nature.

[62] Kudchadkar R, Paraiso KH, Smalley KS (2012). Targeting mutant BRAF in melanoma: current status and future development of combination therapy strategies. Cancer J.

[63] Rebecca VW, Sondak VK, Smalley KS (2012). A brief history of melanoma: from mummies to mutations. Melanoma Res.

[64] Smalley KS, Lioni M, Dalla Palma M, Xiao M, Desai B, Egyhazi S, Hansson J, Wu H, King AJ, Van Belle P, Elder DE, Flaherty KT, Herlyn M, Nathanson KL (2008). Increased cyclin D1 expression can mediate BRAF inhibitor resistance in BRAF V600E-mutated melanomas. Mol Cancer Ther.

[65] Corcoran RB, Dias-Santagata D, Bergethon K, Iafrate AJ, Settleman J, Engelman JA (2010). BRAF gene amplification can promote acquired resistance to MEK inhibitors in cancer cells harboring the BRAF V600E mutation. Sci Signal.

[66] Shi H, Moriceau G, Kong X, Lee MK, Lee H, Koya RC, Ng C, Chodon T, Scolyer RA, Dahlman KB, Sosman JA, Kefford RF, Long GV, Nelson SF, Ribas A, Lo RS (2012). Melanoma whole-exome sequencing identifies (V600E) B-RAF amplification-mediated acquired B-RAF inhibitor resistance. Nat Commun.

[67] Johannessen CM, Boehm JS, Kim SY, Thomas SR, Wardwell L, Johnson LA, Emery CM, Stransky N, Cogdill AP, Barretina J, Caponigro G, Hieronymus H, Murray RR, Salehi-Ashtiani K, Hill DE, Vidal M (2010). COT drives resistance to RAF inhibition through MAP kinase pathway reactivation. Nature.

[68] Cusack K, Allen H, Bischoff A, Clabbers A, Dixon R, Fix-Stenzel S, Friedman M, Gaumont Y, George D, Gordon T, Grongsaard P, Janssen B, Jia Y, Moskey M, Quinn C, Salmeron A (2009). Identification of a selective thieno[2,3-c]pyridine inhibitor of COT kinase and TNF-alpha production. Bioorg Med Chem Lett.

[69] Villanueva J, Vultur A, Lee JT, Somasundaram R, Fukunaga-Kalabis M, Cipolla AK, Wubbenhorst B, Xu X, Gimotty PA, Kee D, Santiago-Walker AE, Letrero R, D'Andrea K, Pushparajan A, Hayden JE, Brown KD (2010). Acquired resistance to BRAF inhibitors mediated by a RAF kinase switch in melanoma can be overcome by cotargeting MEK and IGF-1R/PI3K. Cancer Cell.

[70] Paraiso KH, Xiang Y, Rebecca VW, Abel EV, Chen YA, Munko AC, Wood E, Fedorenko IV, Sondak VK, Anderson AR, Ribas A, Palma MD, Nathanson KL, Koomen JM, Messina JL, Smalley KS (2011). PTEN loss confers BRAF inhibitor resistance to melanoma cells through the suppression of BIM expression. Cancer Res.

[71] Chen J, Shen Q, Labow M, Gaither LA (2011). Protein kinase D3 sensitizes RAF inhibitor RAF265 in melanoma cells by preventing reactivation of MAPK signaling. Cancer Res.

[72] Sharlow ER, Giridhar KV, LaValle CR, Chen J, Leimgruber S, Barrett R, Bravo-Altamirano K, Wipf P, Lazo JS, Wang QJ (2008). Potent and selective disruption of protein kinase D functionality by a benzoxoloazepinolone. J Biol Chem.

[73] Straussman R, Morikawa T, Shee K, Barzily-Rokni M, Qian ZR, Du J, Davis A, Mongare MM, Gould J, Frederick DT, Cooper ZA, Chapman PB, Solit DB, Ribas A, Lo RS, Flaherty KT (2012). Tumour micro-environment elicits innate resistance to RAF inhibitors through HGF secretion. Nature.

[74] Wilson TR, Fridlyand J, Yan Y, Penuel E, Burton L, Chan E, Peng J, Lin E, Wang Y, Sosman J, Ribas A, Li J, Moffat J, Sutherlin DP, Koeppen H, Merchant M (2012). Widespread potential for growth-factor-driven resistance to anticancer kinase inhibitors. Nature.

[75] Sebolt-Leopold JS (2008). Advances in the development of cancer therapeutics directed against the Ras-mitogen-activated protein kinase pathway. Clin Cancer Res.

[76] Haura EB, Ricart AD, Larson TG, Stella PJ, Bazhenova L, Miller VA, Cohen RB, Eisenberg PD, Selaru P, Wilner KD, Gadgeel SM (2010). A phase II study of PD-0325901, an oral MEK inhibitor, in previously treated patients with advanced non-small cell lung cancer. Clin Cancer Res.

[77] LoRusso PM, Krishnamurthi SS, Rinehart JJ, Nabell LM, Malburg L, Chapman PB, DePrimo SE, Bentivegna S, Wilner KD, Tan W, Ricart AD (2010). Phase I pharmacokinetic and pharmacodynamic study of the oral MAPK/ERK kinase inhibitor PD-0325901 in patients with advanced cancers. Clin Cancer Res.

[78] Wong H, Vernillet L, Peterson A, Ware JA, Lee L, Martini JF, Yu P, Li C, Del Rosario G, Choo EF, Hoeflich KP, Shi Y, Aftab BT, Aoyama R, Lam ST, Belvin M (2012). Bridging the gap between preclinical and clinical studies using pharmacokinetic-pharmacodynamic (PK-PD) modeling: An analysis of GDC-0973, a MEK Inhibitor. Clin Cancer Res.

[79] Davies BD, Logie A, McKay JS, Martin P, Steele S, Jenkins R, Cockerill M, Cartlidge S, Smith PD (2007). AZD6244 (ARRY 142886) a potent inhibitor of mitogen-activated protein kinase/extracellular signal-related kinase kinase 1 /2 kinases: mechanism of action in vivo, pharmacokinetic/pharmacodynamic relationship and potential for combination in preclinical models. Mol Cancer Ther.

[80] Schmidt CM, McKillop IH, Cahill PA, Sitzmann JV (1997). Increased MAPK expression and activity in primary human hepatocellular carcinoma. Biochem Biophys Res Commun.

[81] Wiesenauer CA, Yip-Schneider MT, Wang Y, Schmidt CM (2004). Multiple anticancer effects of blocking MEK-ERK signaling in hepatocellular carcinoma. J Am Coll Surg.

[82] Wentz SC, Wu H, Yip-Schneider MT, Hennig M, Klein PJ, Sebolt-Leopold, Schmidt CM (2008). Targeting MEK is effective chemoprevention of hepatocellular carcinoma in TGF-alpha-transgenic mice. J Gastrointest Surg.

[83] Iverson C, Larson G, Lai C, Yeh LT, Dadson C, Weingarten P, Appleby T, Vo T, Maderna A, Vernier JM, Hamatake R, Miner JN, Quart B (2009). RDEA119/BAY 869766: a potent, selective, allosteric inhibitor of MEK1/2 for the treatment of cancer. Cancer Res.

[84] Liu D, Xing J, Trink B, Xing M (2010). BRAF mutation-selective inhibition of thyroid cancer cells by the novel MEK inhibitor RDEA119 and genetic-potentiated synergism with the mTOR inhibitor temsirolimus. Int J Cancer.

[85] Chang Q, Chapman MS, Miner JN, Hedley DW (2010). Antitumour activity of a potent MEK inhibitor RDEA119/BAY 869766 combined with rapamycin in human orthotopic primary pancreatic cancer xenografts. BMC Cancer.

[86] Greger J, Eastman S, Zhang V, Bleam MR, Hughes A, Smitheman KN, Dickerson S, Laquerre S, Liu L, Gilmer TM (2012). Combinations of BRAF, MEK, and PI3K/mTOR inhibitors overcome acquired resistance to the BRAF inhibitor GSK2118436 dabrafenib, mediated by NRAS or MEK mutations. Mol Cancer Ther.

[87] Hoeflich KP, Merchant M, Orr C, Chan J, Den Otter D, Berry L, Kasman I, Koeppen H, Rice K, Yang NY, Engst S, Johnston S, Friedman LS, Belvin M (2012). Intermittent administration of MEK inhibitor GDC-0973 plus PI3K inhibitor GDC-0941 triggers robust apoptosis and tumor growth inhibition. Cancer Res.

[88] Yoon J, Koo KH, Choi KY (2011). MEK1/2 inhibitors AS703026 and AZD6244 may be potential therapies for KRAS mutated colorectal cancer that is resistant to EGFR monoclonal antibody therapy. Cancer Res.

[89] Kim K, Kong SY, Fulciniti M, Li X, Song W, Nahar S, Burger P, Rumizen MJ, Podar K, Chauhan D, Hideshima T, Munshi NC, Richardson P, Clark A, Ogden J, Goutopoulos A (2010). Blockade of the MEK/ERK signalling cascade by AS703026, a novel selective MEK1/2 inhibitor, induces pleiotropic anti-myeloma activity in vitro and in vivo. Br J Haematol.

[90] Lee L, Niu H, Rueger R, Igawa Y, Deutsch J, Ishii N, Mu S, Sakamoto Y, Busse-Reid R, Gimmi C, Goelzer P, De Schepper S, Yoshimura Y, Barrett J, Ishikawa Y, Weissgerber G (2009). The safety, tolerability, pharmacokinetics, and pharmacodynamics of single oral doses of CH4987655 in healthy volunteers: target suppression using a biomarker. Clin Cancer Res.

[91] Dong Q, Dougan DR, Gong X, Halkowycz P, Jin B, Kanouni T, O'Connell SM, Scorah N, Shi L, Wallace MB, Zhou F (2011). Discovery of TAK-733, a potent and selective MEK allosteric site inhibitor for the treatment of cancer. Bioorg Med Chem Lett.

[92] Longoni R, Spina L, Vinci S, Acquas E (2011). The MEK inhibitor SL327 blocks acquisition but not expression of lithium-induced conditioned place aversion: a behavioral and immunohistochemical study. Psychopharmacology (Berl).

[93] Tanios Bekaii-Saab T, Phelps MA, Li X, Saji M, Goff L, Kauh JSW, O'Neil BH, Balsom S, Balint S, Liersemann R, Vasko VV, Bloomston M, Marsh W, Doyle LA, Ellison G, Grever M (2011). Multi-institutional phase II study of selumetinib in patients with metastatic biliary cancers. JCO.

[94] Cusimano A, Azzolina A, Iovanna JL, Bachvarov D, McCubrey JA, D'Alessandro N, Montalto G, Cervello M (2010). Novel combination of celecoxib and proteasome inhibitor MG132 provides synergistic antiproliferative and proapoptotic effects in human liver tumor cells. Cell Cycle.

[95] Lampiasi N, Azzolina A, Umezawa K, Montalto G, McCubrey JA, Cervello M (2012). The novel NF-kappaB inhibitor DHMEQ synergizes with celecoxib to exert antitumor effects on human liver cancer cells by a ROS-dependent mechanism. Cancer Lett.

[96] Kuo MT, Savaraj N, Feun LG (2010). Targeted cellular metabolism for cancer chemotherapy with recombinant arginine-degrading enzymes. Oncotarget.

[97] Vucur M, Roderburg C, Bettermann K, Tacke F, Heikenwalder M, Trautwein C, Luedde T (2010). Mouse models of hepatocarcinogenesis: what can we learn for the prevention of human hepatocellular carcinoma?. Oncotarget.

[98] Dang CV (2010). Glutaminolysis: supplying carbon or nitrogen or both for cancer cells?. Cell Cycle.

[99] Liu Y, Fuchs J, Li C, Lin J (2010). IL-6, a risk factor for hepatocellular carcinoma: FLLL32 inhibits IL-6-induced STAT3 phosphorylation in human hepatocellular cancer cells. Cell Cycle.

[100] Martinez-Garcia M, Banerji U, Albanell J, Bahleda R, Dolly S, Kraeber-Bodéré F, Rojo F, Routier E, Guarin E, Xu ZX, Rueger R, Tessier JJ, Shochat E, Blotner S, Naegelen VM, Soria JC (2012). First-in-Human, Phase I Dose-Escalation Study of the Safety, Pharmacokinetics, and Pharmacodynamics of RO5126766, a First-in-Class Dual MEK/RAF Inhibitor in Patients with Solid Tumors. Clin Cancer Res.

[101] Pratilas CA, Hanrahan AJ, Halilovic E, Persaud Y, Soh J, Chitale D, Shigematsu H, Yamamoto H, Sawai A, Janakiraman M, Taylor BS, Pao W, Toyooka S, Ladanyi M, Gazdar A, Rosen N (2008). Genetic predictors of MEK dependence in non-small cell lung cancer. Cancer Res.

[102] Wee S, Jagani Z, Xiang KX, Loo A, Dorsch M, Yao YM, Seller WR, Lengauer C, Stegmeier F (2099). PI3K pathway activation mediates resistance to MEK inhibitors in KRAS mutant cancers. Cancer Res.

[103] Hoeflich KP, O'Brien C, Boyd Z, Cavet G, Guerrero S, Jung K, Januario T, Savage H, Punnoose E, Truong T, Zhou W, Berry L, Murray L, Amler L, Belvin M, Friedman LS (2009). In vivo antitumor activity of MEK and phosphatidylinositol 3-kinase in basal-like breast cancer models. Clin Cancer Res.

[104] Faber AC, Wong KK, Engelman JA (2010). Differences underlying EGFR and HER2 oncogene addiction. Cell Cycle.

[105] Rudloff U, Samuels Y (2010). A growing family: adding mutated Erbb4 as a novel cancer target. Cell Cycle.

[106] Raven JF, Williams V, Wang S, Tremblay ML, Muller WJ, Durbin JE, Koromilas AE (2011). Stat1 is a suppressor of ErbB2/Neu-mediated cellular transformation and mouse mammary gland tumor formation. Cell Cycle.

[107] Ponzo MG, Park M (2010). The Met receptor tyrosine kinase and basal breast cancer. Cell Cycle.

[108] Steelman LS, Navolanic P, Chappell WH, Abrams SL, Wong EW, Martelli AM, Cocco L, Stivala F, Libra M, Nicoletti F, Drobot LB, Franklin RA, McCubrey JA (2011). Cell Cycle. Involvement of Akt and mTOR in chemotherapeutic- and hormonal-based drug resistance and response to radiation in breast cancer cells. Cell Cycle.

[109] Jiang Z, Jones R, Liu JC, Deng T, Robinson T, Chung PE, Wang S, Herschkowitz JI, Egan SE, Perou CM, Zacksenhaus E (2011). RB1 and p53 at the crossroad of EMT and triple-negative breast cancer. Cell Cycle.

[110] Lehn S, Ferno M, Jirstrom K, Ryden L, Landberg G (2011). A non-functional retinoblastoma tumor suppressor (RB) pathway in premenopausal breast cancer is associated with resistance to tamoxifen. Cell Cycle.

[111] Musgrove EA, Sutherland RL (2010). RB in breast cancer: differential effects in estrogen receptor-positive and estrogen receptor-negative disease. Cell Cycle.

[112] Glazer RI (2010). A new therapeutic basis for treating Li-Fraumeni Syndrome breast tumors expressing mutated TP53. Oncotarget.

[113] Herbert BS, Chanoux RA, Liu Y, Baenziger PH, Goswami CP, McClintick JN, Edenberg HJ, Pennington RE, Lipkin SM, Kopelovich L (2010). A molecular signature of normal breast epithelial and stromal cells from Li-Fraumeni syndrome mutation carriers. Oncotarget.

[114] Susila A, Chan H, Loh AX, Phang HQ, Wong ET, Tergaonkar V, Koh CG (2010). The POPX2 phosphatase regulates cancer cell motility and invasiveness. Cell Cycle.

[115] Harris JL, Khanna KK (2011). BRCA1 A-complex fine tunes repair functions of BRCA1. Aging.

[116] Dever SM, Golding SE, Rosenberg E, Adams BR, Idowu MO, Quillin JM, Valerie N, Xu B, Povirk LF, Valerie K (2011). Mutations in the BRCT binding site of BRCA1 result in hyper-recombination. Aging.

[117] Napoli M, Girardini JE, Piazza S, Del Sal G (2011). Wiring the oncogenic circuitry: Pin1 unleashes mutant p53. Oncotarget.

[118] Azmi AS, Banerjee S, Ali S, Wang Z, Bao B, Beck FW, Maitah M, Choi M, Shields TF, Philip PA, Sarkar FH, Mohammad RM (2011). Network modeling of MDM2 inhibitor-oxaliplatin combination reveals biological synergy in wt-p53 solid tumors. Oncotarget.

[119] Ertel A, Dean JL, Rui H, Liu C, Witkiewicz AK, Knudsen KE, Knudsen ES (2010). RB-pathway disruption in breast cancer: differential association with disease subtypes, disease-specific prognosis and therapeutic response. Cell Cycle.

[120] Santarosa M, Del Col L, Viel A, Bivi N, D'Ambrosio C, Scaloni A, Tell G, Maestro R (2010). BRCA1 modulates the expression of hnRNPA2B1 and KHSRP. Cell Cycle.

[121] Caldon CE, Sutherland RL, Musgrove E (2010). Cell cycle proteins in epithelial cell differentiation: implications for breast cancer. Cell Cycle.

[122] Radojicic J, Zaravinos A, Vrekoussis T, Kafousi M, Spandidos DA, Stathopoulos EN (2011). MicroRNA expression analysis in triple-negative (ER, PR and Her2/neu) breast cancer. Cell Cycle.

[123] Ma S, Guan XY (2011). MiRegulators in cancer stem cells of solid tumors. Cell Cycle.

[124] Valastyan S, Weinberg RA (2010). miR-31: a crucial overseer of tumor metastasis and other emerging roles. Cell Cycle.

[125] Chang S, Sharan SK (2012). Epigenetic control of an oncogenic microRNA, miR-155, by BRCA1. Oncotarget.

[126] Viloria-Petit AM, Wrana JL (2010). The TGFbeta-Par6 polarity pathway: linking the Par complex to EMT and breast cancer progression. Cell Cycle.

[127] Jordan NV, Johnson GL, Abell AN (2011). Tracking the intermediate stages of epithelial-mesenchymal transition in epithelial stem cells and cancer. Cell Cycle.

[128] Cufi S, Vazquez-Martin A, Oliveras-Ferraros C, Martin-Castillo B, Joven J, Menendez JA (2010). Metformin against TGFbeta-induced epithelial-to-mesenchymal transition (EMT): from cancer stem cells to aging-associated fibrosis. Cell Cycle.

[129] Vazquez-Martin A, Oliveras-Ferraros C, Cufi S, Del Barco S, Martin-Castillo B, Menendez JA (2010). Metformin regulates breast cancer stem cell ontogeny by transcriptional regulation of the epithelial-mesenchymal transition (EMT) status. Cell Cycle.

[130] Basu D, Montone KT, Wang LP, Gimotty PA, Hammond R, Diehl JA, Rustgi AK, Lee JT, Rasanen K, Weinstein GS, Herlyn M (2011). Detecting and targeting mesenchymal-like subpopulations within squamous cell carcinomas. Cell Cycle.

[131] Guirouilh-Barbat JK, Wilhelm T, Lopez BS (2010). AKT1/BRCA1 in the control of homologous recombination and genetic stability: the missing link between hereditary and sporadic breast cancers. Oncotarget.

[132] Zhou XZ (2011). PinX1: a sought-after major tumor suppressor at human chromosome 8p23. Oncotarget.

[133] Kutanzi KR, Koturbash I, Kovalchuk O (2010). Reversibility of pre-malignant estrogen-induced epigenetic changes. Cell Cycle.

[134] Prencipe M, McGoldrick A, Perry AS, O'Grady A, Phelan S, McGrogan B, Fitzpatrick P, Watson JA, Furlong F, Brennan DJ, Lawler M, Kay E, McCann A (2010). MAD2 downregulation in hypoxia is independent of promoter hypermethylation. Cell Cycle.

[135] Chiavarina B, Whitaker-Menezes D, Migneco G, Martinez-Outschoorn UE, Pavlides S, Howell A, Tanowitz HB, Casimiro MC, Wang C, Pestell RG, Grieshaber P, Caro J, Sotgia F, Lisanti MP (2010). HIF1-alpha functions as a tumor promoter in cancer associated fibroblasts, and as a tumor suppressor in breast cancer cells: Autophagy drives compartment-specific oncogenesis. Cell Cycle.

[136] Pavlides S, Tsirigos A, Migneco G, Whitaker-Menezes D, Chiavarina B, Flomenberg N, Frank PG, Casimiro MC, Wang C, Pestell RG, Martinez-Outschoorn UE, Howell A, Sotgia F, Lisanti MP (2010). The autophagic tumor stroma model of cancer: Role of oxidative stress and ketone production in fueling tumor cell metabolism. Cell Cycle.

[137] Martinez-Outschoorn UE, Trimmer C, Lin Z, Whitaker-Menezes D, Chiavarina B, Zhou J, Wang C, Pavlides S, Martinez-Cantarin MP, Capozza F, Witkiewicz AK, Flomenberg N, Howell A, Pestell RG, Caro J, Lisanti MP (2010). Autophagy in cancer associated fibroblasts promotes tumor cell survival: Role of hypoxia, HIF1 induction and NFkappaB activation in the tumor stromal microenvironment. Cell Cycle.

[138] Pavlides S, Tsirigos A, Vera I, Flomenberg N, Frank PG, Casimiro MC, Wang C, Pestell RG, Martinez-Outschoorn UE, Howell A, Sotgia F, Lisanti MP (2010). Transcriptional evidence for the “Reverse Warburg Effect” in human breast cancer tumor stroma and metastasis: similarities with oxidative stress, inflammation, Alzheimer's disease, and “Neuron-Glia Metabolic Coupling”. Aging.

[139] Martinez-Outschoorn UE, Whitaker-Menezes D, Lin Z, Flomenberg N, Howell A, Pestell RG, Lisanti MP, Sotgia F (2011). Cytokine production and inflammation drive autophagy in the tumor microenvironment: role of stromal caveolin-1 as a key regulator. Cell Cycle.

[140] Howell A (2011). Defining bad stroma in human breast tumors. Cell Cycle.

[141] Witkiewicz AK, Kline J, Queenan M, Brody JR, Tsirigos A, Bilal E, Pavlides S, Ertel A, Sotgia F, Lisanti MP (2011). Molecular profiling of a lethal tumor microenvironment, as defined by stromal caveolin-1 status in breast cancers. Cell Cycle.

[142] Martinez-Outschoorn UE, Prisco M, Ertel A, Tsirigos A, Lin Z, Pavlides S, Wang C, Flomenberg N, Knudsen ES, Howell A, Pestell RG, Sotgia F, Lisanti MP (2011). Ketones and lactate increase cancer cell “stemness,” driving recurrence, metastasis and poor clinical outcome in breast cancer: achieving personalized medicine via Metabolo-Genomics. Cell Cycle.

[143] Ambs S, Glynn SA (2011). Candidate pathways linking inducible nitric oxide synthase to a basal-like transcription pattern and tumor progression in human breast cancer. Cell Cycle.

[144] Gubin MM, Calaluce R, Davis JW, Magee JD, Strouse CS, Shaw DP, Ma L, Brown A, Hoffman T, Rold TL, Atasoy U (2010). Overexpression of the RNA binding protein HuR impairs tumor growth in triple negative breast cancer associated with deficient angiogenesis. Cell Cycle.

[145] Miller KR, Kelley K, Tuttle R, Berberich SJ (2010). HdmX overexpression inhibits oncogene induced cellular senescence. Cell Cycle.

[146] Kent S, Hutchinson J, Balboni A, Decastro A, Cherukuri P, Direnzo J (2011). ΔNp63alpha promotes cellular quiescence via induction and activation of Notch3. Cell Cycle.

[147] Little AS, Smith PD, Cook SJ (2012). Mechanisms of acquired resistance to ERK1/2 pathway inhibitors. Oncogene.

[148] Little S, Balmanno K, Sale MJ, Newman S, Dry JR, Hampson M, Edwards PAW, Smith PD, Cook SJ (2011). Amplification of the driving oncogene, KRAS or BRAF, underpins acquired resistance to MEK1/2 inhibitors in colorectal cancer cells. Sci Signal.

[149] Hatzivassiliou G, Liu B, O'Brien C, Spoerke JM, Hoeflich KP, Haverty PM, Soriano R, Forrest WF, Heldens S, Chen H, Toy K, Ha C, Zhou W, Song K, Friedman LS, Amler LC (2012). ERK inhibition overcomes acquired resistance to MEK inhibitors. Mol Cancer Ther.

[150] Konopleva M, Milella M, Ruvolo P, Watts JC, Ricciardi MR, Korchin B, McQueen T, Bornmann W, Tsao T, Bergamo P, Mak DH, Chen W, McCubrey J, Tafuri A, Andreeff M (2012). MEK inhibition enhances ABT-737-induced leukemia cell apoptosis via prevention of ERK-activated MCL-1 induction and modulation of MCL-1/BIM complex. Leukemia.

[151] Ricciardi MR, Scerpa MC, Bergamo P, Ciuffreda L, Petrucci MT, Chiaretti S, Tavolaro S, Mascolo MG, Abrams SL, Steelman LS, Tsao T, Marchetti A, Konopleva M, Del Bufalo D, Cognetti F, Foà R (2012). Therapeutic potential of MEK inhibition in acute myelogenous leukemia: rationale for “vertical” and “lateral” combination strategies. J Mol Med (Berl).

[152] Aronov AM, Tang Q, Martinez-Botella G, Bemis GW, Cao J, Chen G, Ewing NP, Ford PJ, Germann UA, Green J, Hale MR, Jacobs M, Janetka JW, Maltais F, Markland W, Namchuk MN (2009). Structure-guided design of potent and selective pyrimidylpyrrole inhibitors of extracellular signal-regulated kinase (ERK) using conformational control. J Med Chem.

[153] Yang L, Dan HC, Sun M, Liu Q, Sun XM, Feldman RI, Hamilton AD, Polokoff M, Nicosia SV, Herlyn M, Sebti SM, Cheng JQ (2004). Akt/protein kinase B signaling inhibitor-2, a selective small molecule inhibitor of Akt signaling with antitumor activity in cancer cells overexpressing Akt. Cancer Res.

[154] Fala F, Blalock WL, Tazzari P, Cappellini A, Chiarini F, Martinelli G, Tafuri A, McCubrey JA, Cocco L, Martelli AM (2008). Proapoptotic activity and chemosensitizing effect of the novel Akt inhibitor (2S)-1-(1H-Indol-3-yl)-3-[5-(3-methyl-2H-indazol-5-yl)pyridin-3-yl]oxypropan2-amine (A443654) in T acute lymphoblastic leukemia. Molecular Pharmacology.

[155] Mandal M, Younes M, Swan EA, Jasser SA, Doan D, Yigitbasi O, McMurphey A, Ludwick J, El-Naggar AK, Bucana C, Mills GB, Myers JN (2006). The Akt inhibitor KP372-1 inhibits proliferation and induces apoptosis and anoikis in squamous cell carcinoma of the head and neck. Oral Oncol.

[156] Tazzari PL, Tabellini G, Ricci F, Papa V, Bortul R, Chiarini F, Evangelisti C, Martinelli G, Bontadini A, Cocco L, McCubrey JA, Martelli AM (2008). Synergistic proapoptotic activity of recombinant trail plus the akt inhibitor perifosine in acute myelogenous leukemia cells. Cancer Res.

[157] Bressanin D, Evangelisti C, Ricci F, Tabellini G, Chiarini F, Tazzari PL, Melchionda F, Buontempo F, Pagliaro P, Pession A, McCubrey JA, Martelli AM (2012). Harnessing the PI3K/Akt/mTOR pathway in T-cell acute lymphoblastic leukemia: Eliminating activity by targeting at different levels. Oncotarget.

[158] Owonikoko T, Khuri ER, Ramalingam SS (2009). Preoperative therapy for early-stage NSCLC: oppurtunities and challenges. Oncology.

[159] Tamburini J, Green AS, Chapuis N, Bardet V, Lacombe C, Mayeux P, Bouscary D (2009). Targeting translation in acute myeloid leukemia: a new paradigm for therapy?. Cell Cycle.

[160] Donia M, McCubrey JA, Bendtzen K, Nicoletti F (2010). Potential use of rapamycin in HIV infection. Br J Clin Pharmacol.

[161] Fouladi M, Laningham F, Wu J, O'Shaughnessy MA, Molina K, Broniscer A, Spunt SL, Luckett I, Stewart CF, Houghton PJ, Gilbertson RJ, Furman WL (2007). Phase I study of Everolimus in pediatric patients with refractory solid tumors. JCO.

[162] Wymann MP, Bulgarelli-Leva G, Zvelebil MJ, Pirola L, Vanhaesebroeck B, Waterfield MD, Panayotou G (1996). Wortmannin inactivates phosphoinositide 3-kinase by covalent modification of Lys-802, a residue involved in the phosphate transfer reaction. Mol Cell Biol.

[163] Vlahos CJ, Matter WF, Hui KY, Brown RF (1994). A specific inhibitor of phosphatidylinositol 3-kinase, 2-(4-morpholinyl)-8-phenyl-4H-1-benzopyran-4-one (LY294002). J Biol Chem.

[164] Gharbi SI, Zvelebil MJ, Shuttleworth SJ, Hancox T, Saghir N, Timms JF, Waterfield MD (2007). Exploring the specificity of the PI3K family inhibitor LY294002. Biochem J.

[165] Garcia-Echeverria C, Sellers WR (2008). Drug discovery approaches targeting the PI3K/Akt pathway in cancer. Oncogene.

[166] Xu R, Spencer VA, Groesser DL, Bissell MJ (2010). Laminin regulates PI3K basal localization and activation to sustain STAT5 activation. Cell Cycle.

[167] Kandouz M, Haidara K, Zhao J, Brisson ML, Batist G (2010). The EphB2 tumor suppressor induces autophagic cell death via concomitant activation of the ERK1/2 and PI3K pathways. Cell Cycle.

[168] Kim DA, Lee BL, Suh EK (2011). Ionizing radiation-induced TAp63alpha phosphorylation at C-terminal S/TQ motifs requires the N-terminal transactivation (TA) domain. Cell Cycle.

[169] Chen Y, Chen CF, Riley DJ, Chen PL (2011). Nek1 kinase functions in DNA damage response and checkpoint control through a pathway independent of ATM and ATR. Cell Cycle.

[170] Leontieva OV, Blagosklonny MV (2011). Yeast-like chronological senescence in mammalian cells: phenomenon, mechanism and pharmacological suppression. Aging.

[171] Ihle NT, Williams R, Chow S, Chew W, Berggren MI, Paine-Murrieta G, Minion DJ, Halter RJ, Wipf P, Abraham R, Kirkpatrick L, Powis G (2004). Molecular pharmacology and antitumor activity of PX-866, a novel inhibitor of phosphoinositide-3-kinase signaling. Mol Cancer Ther.

[172] Koul D, Shen R, Kim YW, Kondo Y, Lu Y, Bankson J, Ronen SM, Kirkpatrick DL, Powis G, Yung WK (2010). Cellular and in vivo activity of a novel PI3K inhibitor, PX-866, against human glioblastoma. Neuro Oncol.

[173] Burrows N, Babur M, Resch J, Ridsdale S, Mejin M, Rowling EJ, Brabant G, Williams KJ (2011). GDC-0941 inhibits metastatic characteristics of thyroid carcinomas by targeting both the phosphoinositide-3 kinase (PI3K) and hypoxia-inducible factor-1-alpha (HIF-1-alpha) pathways. J Clin Endocrinol Metab.

[174] Zou ZQ, Zhang LN, Wang F, Bellenger J, Shen YZ, Zhang XH (2012). The novel dual PI3K/mTOR inhibitor GDC-0941 synergizes with the MEK inhibitor U0126 in non-small cell lung cancer cells. Mol Med Report.

[175] Sujobert P, Bardet V, Cornillet-Lefebvre P, Hayflick JS, Prie N, Verdier F, Vanhaesebroeck B, Muller O, Pesce F, Ifrah N, Hunault-Berger M, Berthou C, Villemagne B, Jourdan E, Audhuy B, Solary E (2005). Essential role for the p110d isoform in phosphoinositide 3-kinase activation and cell proliferation in acute myeloid leukemia. Blood.

[176] Billottet C, Grandage VL, Gale RE, Quattropani A, Rommel C, Vanhaesebroeck B, Khwaja A (2006). A selective inhibitor of the p110d isoform of PI 3-kinase inhibits AML cell proliferation and survival and increases the cytotoxic effects of VP16. Oncogene.

[177] Tamburini J, Chapuis N, Bardet V, Park S, Sujobert P, Willems L, Ifrah N, Dreyfus F, Mayeux P, Lacombe C, Bouscary D (2008). Mammalian target of rapamycin (mTOR) inhibition activates phosphatidylinositol 3-kinase/Akt by up-regulating insulin-like growth factor-1 receptor signaling in acute myeloid leukemia: rationale for therapeutic inhibition of both pathways. Blood.

[178] Workman P, van Montfort RL (2010). PI(3) kinases: revealing the delta lady. Nat Chem Biol.

[179] Workman P, Clarke PA, Raynaud FI, van Montfort RL (2010). Drugging the PI3 kinome: from chemical tools to drugs in the clinic. Cancer Res.

[180] Berndt A, Miller S, Williams O, Le DD, Houseman BT, Pacold JI, Gorrec F, Hon WC, Liu Y, Rommel C, Gaillard P, Rückle T, Schwarz MK, Shokat KM, Shaw JP, Williams RL (2010). The p110 delta structure: mechanisms for selectivity and potency of new PI(3)K inhibitors. Nat Chem Biol.

[181] Lannutti BJ, Meadows SA, Herman SE, Kashishian A, Steiner B, Johnson AJ, Byrd C, Tyner JW, Loriaux MM, Deininger M, Druker BJ, Puri KD, Ulrich RG, Giese NA (2011). CAL-101, a p110d selective phosphatidylinositol-3-kinase inhibitor for the treatment of B-cell malignancies, inhibits PI3K signaling and cellular viability. Blood.

[182] Meadows SA, Vega F, Kashishian A, Johnson D, Diehl V, Miller LL, Younes A, Lannutti BJ (2012). PI3K-delta inhibitor, GS-1101 (CAL-101), attenuates pathway signaling, induces apoptosis, and overcomes signals from the microenvironment in cellular models of Hodgkin lymphoma. Blood.

[183] Gale S, Croasdell G (2010). 28th Annual JP Morgan healthcare conference--Exelixis and Nektar therapeutics. IDrugs.

[184] Maira SM, Pecchi S, Huang A, Burger M, Knapp M, Sterker D, Schnell C, Guthy D, Nagel T, Wiesmann M, Brachmann S, Fritsch C, Dorsch M, Chène P, Shoemaker K, De Pover A (2012). Identification and characterization of NVP-BKM120, an orally available pan-class I PI3-kinase inhibitor. Mol Cancer Ther.

[185] Bendell JC, Rodon J, Burris HA, de Jonge M, Verweij J, Birle D, Demanse D, De Buck SS, Ru QC, Peters M, Goldbrunner M, Baselga J (2012). Phase I, dose-escalation study of BKM120, an oral pan-Class I PI3K inhibitor, in patients with advanced solid tumors. J Clin Oncol.

[186] Garrett JT, Chakrabarty A, Arteaga CL (2011). Will PI3K pathway inhibitors be effective as single agents in patients with cancer?. Oncotarget.

[187] Brachmann S, Fritsch C, Maira SM, Garcia-Echeverria C (2009). PI3K and mTOR inhibitors: a new generation of targeted anticancer agents. Curr Opin Cell Biol.

[188] Molckovsky A, Siu LL (2008). First in class, first in human phase I results of targeted agents: highlights of the 2008 American Society of Clinical Oncology meeting. J Hematol Oncol.

[189] Altman JK, Sassano A, Platanias LC (2011). Targeting mTOR for the treatment of AML. New agents and new directions. Oncotarget.

[190] Xu CX, Li Y, Yue P, Owonikoko TK, Ramalingam SS, Khuri FR, Sun SY (2011). The combination of RAD001 and NVP-BEZ235 exerts synergistic anticancer activity against non-small cell lung cancer in vitro and in vivo. PLoS One.

[191] Fan QW, Knight ZA, Goldenberg DD, Yu W, Mostov KE, Stokoe D, Shokat KM, Weiss WA (2006). A dual PI3 kinase/mTOR inhibitor reveals emergent efficacy in glioma. Cancer Cell.

[192] Fan QW, Cheng CK, Nicolaides TP, Hackett CS, Knight ZA, Shokat KM, Weiss WA (2007). A dual phosphoinositide-3-kinase a/mTOR inhibitor cooperates with blockade of epidermal growth factor receptor in PTEN-mutant glioma. Cancer Res.

[193] Maira SM, Stauffer F, Brueggen J, Furet P, Schnell C, Fritsc C, Brachmann S, Chene P, De Pover A, Schoemaker K, Fabbro D, Gabriel D, Simonen M, Murphy L, Finan P, Sellers W (2008). Identification and characterization of NVP-BEZ235, a new orally available dual phosphatidylinositol 3-kinase/mammalian target of rapamycin inhibitor with potent in vivo antitumor activity. Mol Cancer Ther.

[194] Chapuis N, Tamburini J, Green AS, Vignon C, Bardet V, Neyret A, Pannetier M, Willems L, Park S, Macone A, Maira SM, Ifrah N, Dreyfus F, Herault O, Lacombe C, Mayeux P (2010). Dual inhibition of PI3K and mTORC1/2 signaling by NVP-BEZ235 as a new therapeutic strategy for acute myeloid leukemia. Clin Cancer Res.

[195] Chiarini F, Fala F, Tazzari PL, Ricci F, Astolfi A, Pession A, Pagliaro P, McCubrey JA, Martelli AM (2009). Dual inhibition of class IA phosphatidylionsitol 3-kinase and mTOR as a new therapeutic option for T-cell acute lymphoblastic leukemia. Cancer Research.

[196] Chiarini F, Grimaldi C, Ricci F, Tazzari PL, Evangelisti C, Ognibene A, Battistelli M, Falcieri E, Melchionda F, Pession A, Pagliaro P, McCubrey JA, Martelli AM (2010). Activity of the novel dual phosphatidylinositol 3-kinasse/mammalian target of rapamycin inhibitor NVP-BEZ235 against T-cell acute lymphoblastic leukemia. Cancer Research.

[197] Schuster K, Zheng J, Arbini AA, Zhang CC, Scaglioni PP (2011). Selective targeting of the mTORC1/2 protein kinase complexes leads to antileukemic effects in vitro and in vivo. Blood Cancer J.

[198] Carracedo A, Ma L, Teruya-Feldstein J, Rojo F, Salmena L, Alimonti A, Egia A, Sasaki AT, Thomas G, Kozma SC, Papa A, Nardella C, Cantley LC, Baselga J, Pandolfi PP (2008). Inhibition of mTORC1 leads to MAPK pathway activation through a PI3K-dependent feedback loop in human cancer. J Clin Invest.

[199] Shuttleworth SJ, Silva FA, Cecil AR, Tomassi CD, Hill TJ, Raynaud FI, Clarke PA, Workman P (2011). Progress in the preclinical discovery and clinical development of class I and dual class I/IV phosphoinositide 3-kinase (PI3K) inhibitors. Curr Med Chem.

[200] Mallon R, Feldberg LR, Lucas J, Chaudhary I, Dehnhardt C, Santos ED, Chen Z, dos Santos O, Ayral-Kaloustian S, Venkatesan A, Hollander I (2011). Antitumor efficacy of PKI-587, a highly potent dual PI3K/mTOR kinase inhibitor. Clin Cancer Res.

[201] Gedaly R, Angulo P, Hundley J, Daily MF, Chen C, Evers BM (2011). PKI-587 and Sorafenib Targeting PI3K/AKT/mTOR and Ras/Raf/MAPK pathways synergistically inhibit HCC cell proliferation. J Surg Res.

[202] Yuan J, Mehta PP, Yin MJ, Sun S, Zou A, Chen J, Rafidi K, Feng Z, Nickel J, Engebretsen J, Hallin J, Blasina A, Zhang E, Nguyen L, Sun M, Vogt PK (2011). PF-04691502, a potent and selective oral inhibitor of PI3K and mTOR kinases with antitumor activity. Mol Cancer Ther.

[203] Mallon R, Hollander I, Feldberg L, Lucas J, Soloveva V, Venkatesan A, Dehnhardt C, Delos Santos E, Chen Z, Dos Santos O, Ayral-Kaloustian S, Gibbons J (2010). Antitumor efficacy profile of PKI-402, a dual phosphatidylinositol 3-kinase/mammalian target of rapamycin inhibitor. Mol Cancer Ther.

[204] Prasad G, Sottero T, Yang X, Mueller S, James CD, Weiss WA, Polley MY, Ozawa T, Berger MS, Aftab DT, Prados MD, Haas-Kogan DA (2011). Inhibition of PI3K/mTOR pathways in glioblastoma and implications for combination therapy with temozolomide. Neuro Oncol.

[205] Mirzoeva OK, Hann B, Hom YK, Debnath J, Aftab D, Shokat K, Korn WM (2011). Autophagy suppression promotes apoptotic cell death in response to inhibition of the PI3K-mTOR pathway in pancreatic adenocarcinoma. J Mol Med (Berl).

[206] Wallin JJ, Edgar KA, Guan J, Berry M, Prior WW, Lee L, Lesnick JD, Lewis C, Nonomiya J, Pang J, Salphati L, Olivero AG, Sutherlin DP, O'Brien C, Spoerke JM, Patel S (2011). GDC-0980 is a novel class I PI3K/mTOR kinase inhibitor with robust activity in cancer models driven by the PI3K pathway. Mol Cancer Ther.

[207] Li T, Wang J, Wang X, Yang N, Chen SM, Tong LJ, Yang CH, Meng LH, Ding J (2010). WJD008, a dual phosphatidylinositol 3-kinase (PI3K)/mammalian target of rapamycin inhibitor, prevents PI3K signaling and inhibits the proliferation of transformed cells with oncogenic PI3K mutant. J Pharmacol Exp Ther.

[208] Ilic N, Utermark T, Widlund HR, Roberts TM (2011). PI3K-targeted therapy can be evaded by gene amplification along the MYC-eukaryotic translation initiation factor 4E (eIF4E) axis. Proc Natl Acad Sci USA.

[209] Muellner MK, Uras IZ, Gapp BV, Kerzendorfer C, Smida M, Lechtermann H, Craig-Mueller N, Colinge J, Duernberger G, Nijman SM (2011). A chemical-genetic screen reveals a mechanism of resistance to PI3K inhibitors in cancer. Nat Chem Biol.

[210] Ding H, Han C, Guo D, Wang D, Duan W, Chen CS, D'Ambrosio SM (2008). Sensitivity to the non-COX inhibiting celecoxib derivative, OSU03012, is p21(WAF1/CIP1) dependent. Int J Cancer.

[211] Lee TX, Packer MD, Huang J, Akhmametyeva EM, Kulp SK, Chen CS, Giovannini M, Jacob A, Welling DB, Chang LS (2009). Growth inhibitory and anti-tumour activities of OSU-03012, a novel PDK-1 inhibitor, on vestibular schwannoma and malignant schwannoma cells. Eur J Cancer.

[212] Falasca M, Chiozzotto D, Godage HY, Mazzoletti M, Riley AM, Previdi S, Potter BV, Broggini M, Maffucci T (2010). A novel inhibitor of the PI3K/Akt pathway based on the structure of inositol 1,3,4,5,6-pentakisphosphate. Br J Cancer.

[213] Yang L, Dan HC, Sun M, Liu Q, Sun XM, Feldman RI, Hamilton AD, Polokoff M, Nicosia SV, Herlyn M, Sebti SM, Cheng JQ (2004). Akt/protein kinase B signaling inhibitor-2, a selective small molecule inhibitor of Akt signaling with antitumor activity in cancer cells overexpressing Akt. Cancer Res.

[214] Garrett CR, Coppola D, Wenham RM, Cubitt CL, Neuger AM, Frost TJ, Lush RM, Sullivan DM, Cheng JQ, Sebti SM (2010). Phase I pharmacokinetic and pharmacodynamic study of triciribine phosphate monodrate, a small-molecule inhibitor of AKT phosphorylation, in adult subjects with solid tumors containing activated AKT. Invest New Drugs.

[215] Tan S, Ng Y, James DE (2011). Next-generation Akt inhibitors provide greater specificity: effects on glucose metabolism in adipocytes. Biochem J.

[216] Simioni C, Neri LM, Tabellini G, Ricci F, Bressanin D, Chiarini F, Evangelisti C, Cani A, Tazzari PL, Melchionda F, Pagliaro P, Pession A, McCubrey JA, Capitani S, Martelli AM (2012). Cytotoxic activity of the novel Akt inhibitor, MK-2206, in T-cell acute lymphoblastic leukemia. Leukemia.

[217] Rhodes N, Heerding DA, Duckett DR, Eberwein DJ, Knick VB, Lansing TJ, McConnell RT, Gilmer TM, Zhang SY, Robell K, Kahana JA, Geske RS, Kleymenova EV, Choudhry AE, Lai Z, Leber JD (2008). Characterization of an Akt kinase inhibitor with potent pharmacodynamic and antitumor activity. Cancer Res.

[218] Zeng Z, Samudio IJ, Zhang W, Estrov Z, Pelicano H, Harris D, Frolova O, Hail N, Chen W, Kornblau SM, Huang P, Lu Y, Mills GB, Andreeff M, Konopleva M (2006). Simultaneous inhibition of PDK1/AKT and Fms-like tyrosine kinase 3 signaling by a small-molecule KP372-1 induces mitochondrial dysfunction and apoptosis in acute myelogenous leukemia. Cancer Res.

[219] Rampling R, Sanson M, Gorlia T, Lacombe D, Lai C, Gharib M, Taal W, Stoffregen C, Decker R, van den Bent MJ (2012). A phase I study of LY317615 (enzastaurin) and temozolomide in patients with gliomas (EORTC trial 26054). Neuro Oncol.

[220] Vansteenkiste J, Ramlau R, von Pawel J, San Antonio B, Eschbach C, Szczesna A, Kennedy L, Visseren-Grul C, Chouaki N, Reck M (2012). A phase II randomized study of cisplatin-pemetrexed plus either enzastaurin or placebo in chemonaive patients with advanced non-small cell lung cancer. Oncology.

[221] Wolff RA, Fuchs M, Di Bartolomeo M, Hossain AM, Stoffregen C, Nicol S, Heinemann V (2012). A double-blind, randomized, placebo-controlled, phase 2 study of maintenance enzastaurin with 5-fluorouracil/leucovorin plus bevacizumab after first-line therapy for metastatic colorectal cancer. Cancer.

[222] Kondapaka SB, Singh SS, Dasmahapatra GP, Sausville EA, Roy KK (2003). Perifosine, a novel alkylphospholipid, inhibits protein kinase B activation. Mol Cancer Ther.

[223] Chiarini F, Del Sole M, Mongiorgi S, Gaboardi GC, Cappellini A, Mantovani I, Follo MY, McCubrey JA, Martelli AM (2008). The novel Akt inhibitor perifosine induces caspase-dependent apoptosis and downregulates P-glycoprotein expression in multidrug-resistant T-acute leukemia cells by a JNK-dependent mechanism. Leukemia.

[224] Pal SK, Reckamp K, Yu H, Figlin RA (2010). Akt inhibitors in clinical development for the treatment of cancer. Expert Opin Investig Drugs.

[225] Handrick R, Rübel A, Faltin H, Eibl H, Belka C, Jendrossek V (2006). Increased cytotoxicity of ionizing radiation in combination with membrane-targeted apoptosis modulators involves downregulation of protein kinase B/Akt-mediated survival-signaling. Radiother Oncol.

[226] Martelli AM, Papa V, Tazzari PL, Evangelesti C, Chiarini F, Grimaldi C, Ricci F, Martinelli G, Ottaviani E, Pagliaro P, Horn S, Basecke J, Linder LH, Eibl H, McCubrey JA (2010). Erucylphosphohomocholine, the first intravenously applicable alkylphosphocholine, is cytotoxic to acute myelogenous leukemia cells through JNK2- and PP2-dependent mechanisms. Leukemia.

[227] Bidyasar S, Kurzrock R, Falchook GS, Naing A, Wheler JJ, Durand J, Yang P, Johansen MJ, Newman RA, Khan R, Hong D (2009). A first-in-human phase I trial of PBI-05204 (oleandrin), an inhibitor of Akt, FGF-2, NF-Kb, and p70S6K in advanced solid tumor patients. Journal of Clinical Oncology, ASCO Annual Meeting Proceedings (Post-Meeting Edition).

[228] Dunn DE, He DN, Yang P, Johansen M, Newman RA, Lo DC (2011). In vitro and in vivo neuroprotective activity of the cardiac glycoside oleandrin from Nerium oleander in brain slice-based stroke models. J Neurochem.

[229] Yoon H, Kim DJ, Ahn EH, Gellert GC, Shay JW, Ahn CH, Lee YB (2009). Antitumor activity of a novel antisense oligonucleotide against Akt1. J Cell Biochem.

[230] Marshall J, Posey J, Hwang J, Malik S, Shen R, Kazempour K, White LR, Fraser KM, Chang CG, Ahn CH (2007). A phase I trial of RX-0201 (AKT anti-sense) in patients with an advanced cancer. Journal of Clinical Oncology, ASCO Annual Meeting Proceedings Part I.

[231] Oshiro N, Yoshino K, Hidayat S, Tokunaga C, Hara K, Eguchi S, Avruch J, Yonezawa K (2004). Dissociation of raptor from mTOR is a mechanism of rapamycin-induced inhibition of mTOR function. Genes Cells.

[232] Bai X, Ma D, Liu A, Shen X, Wang QJ, Liu Y, Jiang Y (2007). Rheb activates mTOR by antagonizing its endogenous inhibitor, FKBP38. Science.

[233] Fouladi M, Laningham F, Wu J, O'Shaughnessy MA, Molina K, Broniscer A, Spunt SL, Luckett I, Stewart CF, Houghton PJ, Gilbertson RJ, Furman WL (2007). Phase I study of Everolimus in pediatric patients with refractory solid tumors. JCO.

[234] Major P (2011). Potential of mTOR inhibitors for the treatment of subependymal giant cell astrocytomas in tuberous sclerosis complex. Aging.

[235] Apontes P, Leontieva OV, Demidenko ZN, Li F, Blagosklonny MV (2011). Exploring long-term protection of normal human fibroblasts and epithelial cells from chemotherapy in cell culture. Oncotarget.

[236] Blagosklonny MV (2011). Molecular damage in cancer: an argument for mTOR-driven aging. Aging.

[237] Leontieva OV, Blagosklonny MV (2011). Yeast-like chronological senescence in mammalian cells: phenomenon, mechanism and pharmacological suppression. Aging.

[238] Williamson DL (2011). Normalizing a hyperactive mTOR initiates muscle growth during obesity. Aging.

[239] Lu MK, Gong XG, Guan KL (2011). mTOR in podocyte function: is rapamycin good for diabetic nephropathy?. Cell Cycle.

[240] Rini BI, Campbell SC, Escudier B (2009). Renal cell carcinoma. Lancet.

[241] Benjamin D, Colombi M, Moroni C, Hall MN (2011). Rapamycin passes the torch: a new generation of mTOR inhibitors. Nat Rev Drug Discov.

[242] Chawla SP, Staddon AP, Baker LH, Schuetze SM, Tolcher AW, D'Amato GZ, Blay JY, Mita MM, Sankhala KK, Berk L, Rivera VM, Clackson T, Loewy JW, Haluska FG, Demetri GD (2012). Phase II study of the mammalian target of rapamycin inhibitor ridaforolimus in patients with advanced bone and soft tissue sarcomas. J Clin Oncol.

[243] Donia M, McCubrey JA, Bendtzen K, Nicoletti F (2010). Potential use of rapamycin in HIV infection. Br J Clin Pharmacol.

[244] Nicoletti F, Fagone P, Meroni P, McCubrey J, Bendtzen K (2011). mTOR as a multifunctional therapeutic target in HIV infection. Drug Discov Today.

[245] Carew JS, Kelly KR, Nawrocki ST (2011). Mechanisms of mTOR inhibitor resistance in cancer therapy. Target Oncol.

[246] Ohh M, Park CW, Ivan M, Hoffman MA, Kim TY, Huang LE, Pavletich N, Chau V, Kaelin WG (2000). Ubiquitination of hypoxiainducible factor requires direct binding to the beta-domain of the von Hippel-Lindau protein. Nat Cell Biol.

[247] Mahalingam D, Medina EC, Esquivel JA, Espitia CM, Smith S, Oberheu K, Swords R, Kelly KR, Mita MM, Mita AC, Carew JS, Giles FJ, Nawrocki ST (2010). Vorinostat enhances the activity of temsirolimus in renal cell carcinoma through suppression of survivin levels. Clin Cancer Res.

[248] Witzig TE, Geyer SM, Ghobrial I, Inwards DJ, Fonseca R, Kurtin P, Ansell SM, Luyun R, Flynn PJ, Morton RF, Dakhil SR, Gross H, Kaufmann SH (2005). Phase II trial of single-agent temsirolimus (CCI-779) for relapsed mantle cell lymphoma. J Clin Oncol.

[249] Dumont FJ, Staruch MJ, Grammer T, Blenis J, Kastner CA, Rupprecht KM (1995). Dominant mutations confer resistance to the immunosuppressant, rapamycin, in variants of a T cell lymphoma. Cell Immunol.

[250] Fruman DA, Wood MA, Gjertson CK, Katz HR, Burakoff SJ, Bierer BE (1995). FK506 binding protein 12 mediates sensitivity to both FK506 and rapamycin in murine mast cells. Eur J Immunol.

[251] Lorenz MC, Heitman J (1995). TOR mutations confer rapamycin resistance by preventing interaction with FKBP12-rapamycin. J Biol Chem.

[252] Fox CJ, Hammerman PS, Thompson CB (2005). The Pim kinases control rapamycin-resistant T cell survival and activation. J Exp Med.

[253] Siu A, Virtanen C, Jongstra J (2011). PIM kinase isoform specific regulation of MIG6 expression and EGFR signaling in prostate cancer cells. Oncotarget.

[254] Forshell LP, Li Y, Forshell TZ, Rudelius M, Nilsson L, Keller U, Nilsson J (2011). The direct Myc target Pim3 cooperates with other Pim kinases in supporting viability of Myc-induced B-cell lymphomas. Oncotarget.

[255] Hammerman PS, Fox CJ, Birnbaum MJ, Thompson CB (2005). Pim and Akt oncogenes are independent regulators of hematopoietic cell growth and survival. Blood.

[256] Beharry Z, Mahajan S, Zemskova M, Lin YW, Tholanikunnel BG, Xia Z, Smith CD, Kraft AS (2011). The Pim protein kinases regulate energy metabolism and cell growth. Proc Natl Acad Sci USA.

[257] Lilly M, Kraft A (1997). Enforced expression of the Mr 33,000 Pim-1 kinase enhances factor-independent survival and inhibits apoptosis in murine myeloid cells. Cancer Res.

[258] Zhang F, Beharry ZM, Harris TE, Lilly MB, Smith CD, Mahajan S, Kraft AS (2009). PIM1 protein kinase regulates PRAS40 phosphorylation and mTOR activity in FDCP1 cells. Cancer Biol Ther.

[259] Tan J, Lee PL, Li Z, Jiang X, Lim YC, Hooi SC, Yu Q (2010). B55beta-associated PP2A complex controls PDK1-directed myc signaling and modulates rapamycin sensitivity in colorectal cancer. Cancer Cell.

[260] Dilling MB, Germain GS, Dudkin L, Jayaraman AL, Zhang X, Harwood FC, Houghton PJ (2002). 4E-binding proteins, the suppressors of eukaryotic initiation factor 4E, are downregulated in cells with acquired or intrinsic resistance to rapamycin. J Biol Chem.

[261] Luo Y, Marx SO, Kiyokawa H, Koff A, Massague J, Marks AR (1996). Rapamycin resistance tied to defective regulation of p27Kip1. Mol Cell Biol.

[262] Gruppuso PA, Boylan JM, Sanders JA (2011). The physiology and pathophysiology of rapamycin resistance: implications for cancer. Cell Cycle.

[263] Neklesa TK, Davis RW (2008). Superoxide anions regulate TORC1 and its ability to bind Fpr1: rapamycin complex. Proc Natl Acad Sci USA.

[264] Trachootham D, Alexandre J, Huang P (2009). Targeting cancer cells by ROS-mediated mechanisms: a radical therapeutic approach?. Nat Rev Drug Discov.

[265] Majumder PK, Febbo PG, Bikoff R, Berger R, Xue Q, McMahon LM, Manola J, Brugarolas J, McDonnell TJ, Golub TR, Loda M, Lane HA, Sellers WR (2004). mTOR inhibition reverses Akt-dependent prostate intraepithelial neoplasia through regulation of apoptotic and HIF-1-dependent pathways. Nat Med.

[266] Guba M, von Breitenbuch P, Steinbauer M, Koehl G, Flegel S, Hornung M, Bruns CJ, Zuelke C, Farkas S, Anthuber M, Jauch KW, Geissler EK (2002). Rapamycin inhibits primary and metastatic tumor growth by antiangiogenesis: involvement of vascular endothelial growth factor. Nat Med.

[267] Stoeltzing O (2010). Dual-targeting of mTOR and HSP90 for cancer therapy: facing oncogenic feed-back-loops and acquired mTOR resistance. Cell Cycle.

[268] Bhagwat SV, Gokhale PC, Crew AP, Cooke A, Yao Y, Mantis C, Kahler J, Workman J, Bittner M, Dudkin L, Epstein DM, Gibson NW, Wild R, Arnold LD, Houghton PJ, Pachter JA (2011). Preclinical characterization of OSI-027, a potent and selective inhibitor of mTORC1 and mTORC2: distinct from rapamycin. Mol Cancer Ther.

[269] Grimaldi C, Chiarini F, Tabellini G, Ricci F, Tazzari PL, Battistelli M, Falcieri E, Bortul R, Melchionda F, Iacobucci I, Pagliaro P, Martinelli G, Pession A, Barata JT, McCubrey JA, Martelli AM (2012). AMP-dependent kinase/mammalian target of rapamycin complex 1 signaling in T-cell acute lymphoblastic leukemia: therapeutic implications. Leukemia.

[270] Carayol N, Vakana E, Sassano A, Kaur S, Goussetis DJ, Glaser H, Druker BJ, Donato NJ, Altman JK, Barr S, Platanias LC (2010). Critical roles for mTORC2-and rapamycin-insensitive mTORC1 complexes in growth and survival of BCR ABL expressing leukemic cells. Proc Natl Acad Sci USA.

[271] Tan DS, Dumez H, Olmos D, Sandhu SK, Hoeben A, Stephens AW, Poondru S, Gedrich R, Kaye SB, Schoffski P (2010). First-in-human phase I study exploring three schedules of OSI-027, a novel small molecule TORC1/TORC2 inhibitor, in patients with advanced solid tumors and lymphoma. J Clin Onco.

[272] Jessen K, Jessen K, Wang S, Kessler L, Guo X, Kucharski J, Staunton J, Lan L, Elia M, Stewart J, Brown J, Li L, Chan K, Martin M, Ren P, Rommel C (2009). INK128 is a potent and selective TORC1/2 inhibitor with broad oral antitumor activity. Mol. Cancer Ther.

[273] Hsieh AC, Ruggero D (2010). Targeting eukaryotic translation initiation factor 4E (eIF4E) in cancer. Clin Cancer Res.

[274] Hsieh AC, Liu Y, Edlind MP, Ingolia NT, Janes MR, Sher A, Shi EY, Stumpf CR, Christensen C, Bonham MJ, Wang S, Ren P, Martin M, Jessen K, Feldman ME, Weissman JS (2012). The translational landscape of mTOR signalling steers cancer initiation and metastasis. Nature.

[275] Chresta CM, Davies BR, Hickson I, Harding T, Cosulich S, Critchlow SE, Vincent JP, Ellston R, Jones D, Sini P, James D, Howard Z, Dudley P, Hughes G, Smith L, Maguire S (2010). AZD8055 is a potent, selective, and orally bioavailable ATP-competitive mammalian target of rapamycin kinase inhibitor with in vitro and in vivo antitumor activity. Cancer Res.

[276] Banerji U, Aghajanian C, Raymond E, Kurzrock R, Blanco-Codesido M, Oelmann E, Grinsted L, Burke W, Kaye SB, Naing A (2011). First results from a phase I trial of AZD8055, a dual mTORC1 and mTORC2 inhibitor. J Clin Oncol.

[277] Xue Q, Hopkins B, Perruzzi C, Udayakumar D, Sherris D, Benjamin LE (2008). Palomid 529, a novel small-molecule drug, is a TORC1/TORC2 inhibitor that reduces tumor growth, tumor angiogenesis, and vascular permeability. Cancer Res.

[278] Yu K, Shi C, Toral-Barza L, Lucas J, Shor B, Kim JE, Zhang WG, Mahoney R, Gaydos C, Tardio L, Kim SK, Conant R, Curran K, Kaplan J, Verheijen J, Ayral-Kaloustian S (2010). Beyond rapalog therapy: preclinical pharmacology and antitumor activity of WYE 125132, an ATP-competitive and specific inhibitor of mTORC1 and mTORC2. Cancer Res.

[279] García-Martínez JM, Moran J, Clarke RG, Gray A, Cosulich SC, Chresta CM, Alessi DR Ku (2009). 0063794 is a specific inhibitor of the mammalian target of rapamycin (mTOR). Biochem J.

[280] Falcon BL, Barr S, Gokhale PC, Chou J, Fogarty J, Depeille P, Miglarese M, Epstein DM, McDonald DM (2011). Reduced VEGF production, angiogenesis, and vascular regrowth contribute to the antitumor properties of dual mTORC1/mTORC2 inhibitors. Cancer Res.

[281] Liu Q, Wang J, Kang SA, Thoreen CC, Hur W, Ahmed T, Sabatini DM, Gray NS (2011). Discovery of 9 (6 aminopyridin-3 yl)-1 (3-(trifluoromethyl)phenyl)benzo[h][1,6] naphthyridin 2(1H)-one (Torin2) as a potent, selective, and orally available mammalian target of rapamycin (mTOR) inhibitor for treatment of cancer. J Med Chem.

[282] Shackelford DB, Shaw RJ (2009). The LKB1-AMPK pathway: metabolism and growth control in tumour suppression. Nat Rev Cancer.

[283] Gwinn DM, Shackelford DB, Egan DF, Mihaylova MM, Mery A, Vasquez DS, Turk BE, Shaw RJ (2008). AMPK phosphorylation of raptor mediates a metabolic checkpoint. Mol Cell.

[284] Noto H, Goto A, Tsujimoto T, Noda M (2012). Cancer Risk in Diabetic Patients Treated with Metformin: A Systematic Review and Meta-analysis. PLoS One.

[285] Anisimov VN, Berstein LM, Popovich IG, Zabezhinski MA, Egormin PA, Piskunova TS, Semenchenko AV, Tyndyk ML, Yurova MN, Kovalenko IG, Poroshina TE (2011). If started early in life, metformin treatment increases life span and postpones tumors in female SHR mice. Aging (Albany NY).

[286] Del Barco S, Vazquez-Martin A, Cufi S, Oliveras-Ferraros C, Bosch-Barrera J, Joven J, Martin-Castillo B, Menendez JA (2011). Metformin: multi-faceted protection against cancer. Oncotarget.

[287] Richardson AD, Scott DA (2011). Reversing the Warburg effect through stromal autophagy. Cell Cycle.

[288] Demaria M, Giorgi C, Lebiedzinska M, Esposito G, D'Angeli L, Bartoli A, Gough DJ, Turkson J, Levy DE, Watson CJ, Wieckowski MR, Provero P, Pinton P, Poli V (2010). A STAT3-mediated metabolic switch is involved in tumour transformation and STAT3 addiction. Aging.

[289] Darnell JE (2010). STAT3, HIF-1, glucose addiction and Warburg effect. Aging.

[290] Menendez JA, Cufi S, Oliveras-Ferraros C, Martin-Castillo B, Joven J, Vellon L, Vazquez-Martin A (2011). Metformin and the ATM DNA damage response (DDR): accelerating the onset of stress-induced senescence to boost protection against cancer. Aging.

[291] Halicka HD, Zhao H, Li J, Traganos F, Zhang S, Lee M, Darzynkiewicz Z (2011). Genome protective effect of metformin as revealed by reduced level of constitutive DNA damage signaling. Aging.

[292] Mackenzie MJ, Ernst S, Johnson C, Winquist E (2010). A phase I study of temsirolimus and metformin in advanced solid tumours. Invest New Drugs.

[293] McMahon LP, Yue W, Santen RJ, Lawrence JC (2005). Farnesylthiosalicylic acid inhibits mammalian target of rapamycin (mTOR) activity both in cells and in vitro by promoting dissociation of the mTOR-raptor complex. Mol Endocrinol.

[294] Appels NM, Beijnen JH, Schellens JH (2005). Development of farnesyl transferase inhibitors: a review. Oncologist.

[295] Killestein J, Rudick RA, Polman CH (2011). Oral treatment for multiple sclerosis. Lancet Neurol.

[296] Neviani P, Santhanam R, Oaks JJ, Eiring AM, Notari M, Blaser BW, Liu S, Trotta R, Muthusamy N, Gambacorti-Passerini C, Druker BJ, Cortes J, Marcucci G, Chen CS, Verrills NM, Roy DC (2007). FTY720, a new alternative for treating blast crisis chronic myelogenous leukemia and Philadelphia chromosome-positive acute lymphocytic leukemia. J Clin Invest.

[297] Molhoek KR, Brautigan DL, Slingluff CL (2003). Synergistic inhibition of human melanoma proliferation by combination treatment with B-Raf inhibitor BAY43-9006 and mTOR inhibitor rapamycin. J Transl Med.

[298] Engleman JA, Chen L, Tan X, Crosby K, Guimaraes AR, Upadhyay R, Maira M, McNamara K, Perera SA, Song Y, Chirieac LR, Kaur R, Lightbown A, Simendinger J, Li T, Padera RF (2008). Effective use of PI3K and MEK inhibitors to treat mutant Kras G12D and PIK3CA H104R murine lung cancers. Nat Med.

[299] Wang Z, Zhou J, Fan J, Qiu SJ, Yu Y, Huang XW, Tang ZY (2008). Effects of rapamycin alone and in combination with sorafenib in an orthotopic model of human hepatocellular carcinoma. Clin Cancer Res.

[300] Jin N, Jiang T, Rosen DM, Nelkin BD, Ball DW (2011). Synergistic action of a RAF inhibitor and a dual PI3K/mTOR inhibitor in thyroid cancer. Clin Cancer Res.

[301] Legrier ME, Yang CP, Yan HG, Lopez-Barcons L, Keller SM, Perez-Soler R, Horwitz SB, McDaid HM (2007). Targeting protein translation in human non-small cell lung cancer via combined MEK and mammalian target of rapamycin suppression. Cancer Res.

[302] Marshall G, Howard Z, Dry J, Fenton S, Heathcote D, Gray N, Keen H, Logie A, Holt S, Smith P, Guichard SM (2011). Benefits of mTOR kinase targeting in oncology: pre-clinical evidence with AZD8055. Biochem Soc Trans.

[303] Chang Q, Chapman MS, Miner JN, Hedley DW (2010). Antitumour activity of a potent MEK inhibitor RDEA119/BAY 869766 combined with rapamycin in human orthotopic primary pancreatic cancer xenografts. BMC Cancer.

[304] Baumann P, Hagemeier H, Mandl-Weber S, Franke D, Schmidmaier R (2009). Myeloma cell growth inhibition is augmented by synchronous inhibition of the insulin-like growth factor 1 receptor by NVP AEW541 and inhibition of mammalian target of rapamycin by Rad001. Anticancer Drugs.

[305] Rao RD, Mladek AC, Lamont JD, Goble JM, Erlichman C, James CD, Sarkaria JN (2005). Disruption of parallel and converging signaling pathways contributes to the synergistic antitumor effects of simultaneous mTOR and EGFR inhibition in GBM cells. Neoplasia.

[306] Cirstea D, Hideshima T, Rodig S, Santo L, Pozzi S, Vallet S, Ikeda H, Perrone G, Gorgun G, Patel K, Desai N, Sportelli P, Kapoor S, Vali S, Mukherjee S, Munshi NC (2010). Dual inhibition of Akt/mammalian target of rapamycin pathway by nanoparticle albumin bound rapamycin and perifosine induces antitumor activity in multiple myeloma. Mol Cancer Ther.

[307] Yang H, Higgins B, Kolinsky K, Packman K, Bradley WD, Lee RJ, Schostack K, Simcox ME, Kopetz S, Heimbrook D, Lestini B, Bollag G, Su F (2012). Antitumor activity of BRAF inhibitor vemurafenib in preclinical models of BRAF-mutant colorectal cancer. Cancer Res.

[308] Paraiso KH, Haarberg E, Wood E, Rebecca VW, Chen YA, Xiang Y, Ribas A, Lo RS, Weber JS, Sondak VK, John J, Sarnaik AA, Koomen J, Smalley KS (2012). The heat shock protein-90 inhibitor XL888 overcomes BRAF inhibitor resistance mediated through diverse mechanisms. Clin Cancer Res.

[309] Shapiro G, LoRusso P, Kwak EL, Cleary JM, Musib L, Jones C, de Crespigny A, Belvin M, McKenzie M, Gates MR, Chan IT, Bendell JC (2011). GDC-0941: A first-in-human phase Ib study testing daily and intermittent dosing schedules in patients with advanced solid tumors. J Clin Oncol.

[310] Flaherty KT (2006). Chemotherapy and targeted therapy combinations in advanced melanoma. Clin Cancer Res.

[311] Borst P, Wessels L (2010). Do predictive signatures really predict response to cancer chemotherapy?. Cell Cycle.

[312] Bertucci F, Finetti P, Sabatier R, Birnbaum D (2010). The CINSARC signature: prognostic and predictive of response to chemotherapy?. Cell Cycle.

[313] Bao W, Chen M, Zhao X, Kumar R, Spinnler C, Thullberg M, Issaeva N, Selivanova G, Stromblad S (2011). PRIMA-1Met/APR-246 induces wild-type p53-dependent suppression of malignant melanoma tumor growth in 3D culture and in vivo. Cell Cycle.

[314] Raffaghello L, Safdie F, Bianchi G, Dorff T, Fontana L, Longo VD (2010). Fasting and differential chemotherapy protection in patients. Cell Cycle.

[315] Hodny Z, Hubackova S, Bartek J (2010). Cytokines shape chemotherapy-induced and ‘bystander’ senescence. Aging.

[316] Luchenko VL, Salcido CD, Zhang Y, Agama K, Komlodi-Pasztor E, Murphy RF, Giaccone G, Pommier Y, Bates SE, Varticovski L (2011). Schedule-dependent synergy of histone deacetylase inhibitors with DNA damaging agents in small cell lung cancer. Cell Cycle.

[317] Dienstmann R, Martinez P, Felip E (2011). Personalizing therapy with targeted agents in non-small cell lung cancer. Oncotarget.

[318] Heasman SA, Zaitseva L, Bowles KM, Rushworth SA, Macewan DJ (2011). Protection of acute myeloid leukaemia cells from apoptosis induced by front-line chemotherapeutics is mediated by haem oxygenase-1. Oncotarget.

[319] Pabla N, Dong Z (2012). Curtailing side effects in chemotherapy: a tale of PKCdelta in cisplatin treatment. Oncotarget.

[320] Stauber RH, Knauer SK, Habtemichael N, Bier C, Unruhe B, Weisheit S, Spange S, Nonnenmacher F, Fetz V, Ginter T, Reichardt S, Liebmann C, Schneider G, Kramer OH (2012). A combination of a ribonucleotide reductase inhibitor and histone deacetylase inhibitors downregulates EGFR and triggers BIM-dependent apoptosis in head and neck cancer. Oncotarget.

[321] Coley HM, Hatzimichael E, Blagden S, McNeish I, Thompson A, Crook T, Syed N (2012). Polo Like kinase 2 tumour suppressor and cancer biomarker: new perspectives on drug sensitivity/resistance in ovarian cancer. Oncotarget.

[322] Oksenych V, Coin F (2010). The long unwinding road: XPB and XPD helicases in damaged DNA opening. Cell Cycle.

[323] Soleimani R, Heytens E, Darzynkiewicz Z, Oktay K (2011). Mechanisms of chemotherapy-induced human ovarian aging: double strand DNA breaks and microvascular compromise. Aging.

[324] Florian S, Mayer TU (2011). Modulated microtubule dynamics enable Hklp2/Kif15 to assemble bipolar spindles. Cell Cycle.

[325] Lee J, Kim JA, Margolis RL, Fotedar R (2010). Substrate degradation by the anaphase promoting complex occurs during mitotic slippage. Cell Cycle.

[326] Tovar C, Higgins B, Deo D, Kolinsky K, Liu JJ, Heimbrook DC, Vassilev LT (2010). Small-molecule inducer of cancer cell polyploidy promotes apoptosis or senescence: Implications for therapy. Cell Cycle.

[327] Shen S, Kepp O, Martins I, Vitale I, Souquere S, Castedo M, Pierron G, Kroemer G (2010). Defective autophagy associated with LC3 puncta in epothilone-resistant cancer cells. Cell Cycle.

[328] Mancias JD, Kimmelman AC (2011). Targeting autophagy addiction in cancer. Oncotarget.

[329] Pasquier E, Ciccolini J, Carre M, Giacometti S, Fanciullino R, Pouchy C, Montero MP, Serdjebi C, Kavallaris M, Andre N (2010). Propranolol potentiates the anti-angiogenic effects and anti-tumor efficacy of chemotherapy agents: implication in breast cancer treatment. Oncotarget.

[330] Cubillos-Ruiz JR, Rutkowski M, Conejo-Garcia JR (2010). Blocking ovarian cancer progression by targeting tumor microenvironmental leukocytes. Cell Cycle.

[331] Antico Arciuch VG, Russo MA, Dima M, Kang KS, Dasrath F, Liao XH, Refetoff S, Montagna C, Di Cristofano A (2011). Thyrocyte-specific inactivation of p53 and Pten results in anaplastic thyroid carcinomas faithfully recapitulating human tumors. Oncotarget.

[332] Steelman LS, Martelli AM, Nicoletti F, McCubrey JA (2011). Exploiting p53 status to enhance effectiveness of chemotherapy by lowering associated toxicity. Oncotarget.

[333] Rao B, van Leeuwen IM, Higgins M, Campbel J, Thompson AM, Lane DP, Lain S (2010). Evaluation of an Actinomycin D/VX-680 aurora kinase inhibitor combination in p53-based cyclotherapy. Oncotarget.

[334] Hu Y, Spengler ML, Kuropatwinski KK, Comas-Soberats M, Jackson M, Chernov MV, Gleiberman AS, Fedtsova N, Rustum YM, Gudkov AV, Antoch MP (2011). Selenium is a modulator of circadian clock that protects mice from the toxicity of a chemotherapeutic drug via upregulation of the core clock protein, BMAL1. Oncotarget.

[335] Steelman LS, Franklin RA, Abrams SL, Chappell W, Kempf CR, Bäsecke J, Stivala F, Donia M, Fagone P, Nicoletti F, Libra M, Ruvolo P, Ruvolo V, Evangelisti C, Martelli AM, McCubrey JA (2011). Roles of the Ras/Raf/MEK/ERK pathway in leukemia therapy. Leukemia.

[336] McDaid HM, Lopez-Barcons L, Grossman A, Lia M, Keller S, Pérez-Soler R, Horwitz SB (2005). Enhancement of the therapeutic efficacy of taxol by the mitogen-activated protein kinase kinase inhibitor CI-1040 in nude mice bearing human heterotransplants. Cancer Res.

[337] Haass NK, Sproesser K, Nguyen TK, Contractor R, Medina CA, Nathanson KL, Herlyn M, Smalley KS (2008). The mitogen-activated protein/extracellular signal-regulated kinase kinase inhibitor AZD6244 (ARRY 142886) induces growth arrest in melanoma cells and tumor regression when combined with docetaxel. Clin Cancer Res.

[338] Morelli MP, Tentler JJ, Kulikowski GN, Tan AC, Bradshaw-Pierce EL, Pitts TM, Brown AM, Nallapareddy S, Arcaroli JJ, Serkova NJ, Hidalgo M, Ciardiello F, Eckhardt SG (2012). Preclinical activity of the rational combination of selumetinib (AZD6244) in combination with vorinostat in KRAS-mutant colorectal cancer models. Clinical Cancer Research.

[339] Hirai H, Sootome H, Nakatsuru Y, Miyama K, Taguchi S, Tsujioka K, Ueno Y, Hatch H, Majumder PK, Pan BS, Kotani H (2010). MK-2206, an allosteric Akt inhibitor, enhances antitumor efficacy by standard chemotherapeutic agents or molecular targeted drugs in vitro and in vivo. Mol Cancer Ther.

[340] Baumann P, Mandl-Weber S, Oduncu F, Schmidmaier R (2009). The novel orally bioavailable inhibitor of phosphoinositol-3 kinase and mammalian target of rapamycin, NVP BEZ235, inhibits growth and proliferation in multiple myeloma. Exp Cell Res.

[341] Manara MC, Nicoletti G, Zambelli D, Ventura S, Guerzoni C, Landuzzi L, Lollini PL, Maira SM, García-Echeverría C, Mercuri M (2010). Picci. NVP BEZ235 as a new therapeutic option for sarcomas. Clin Cancer Res.

[342] Zhang YJ, Duan Y, Zheng XF (2011). Targeting the mTOR kinase domain: the second generation of mTOR inhibitors. Drug Discov Today.

[343] Engelman JA, Chen L, Tan X, Crosby K, Guimaraes AR, Upadhyay R, Maira M, McNamara K, Perera SA, Song Y, Chirieac LR, Kaur R, Lightbown A, Simendinger J, Li T, Padera RF (2008). Effective use of PI3K and MEK inhibitors to treat mutant Kras G12D and PIK3CA H1047R murine lung cancers. Nature Med.

[344] Gravina GL, Marampon F, Petini F, Biordi L, Sherris D, Jannini EA, Tombolini V, Festuccia C (2011). The TORC1/TORC2 inhibitor, Palomid 529, reduces tumor growth and sensitizes to docetaxel and cisplatin in aggressive and hormone refractory prostate cancer cells. Endocr Relat Cancer.

[345] Diaz R, Nguewa PA, Diaz-Gonzalez JA, Hamel E, Gonzalez-Moreno O, Catena R, Serrano D, Redrado M, Sherris D, Calvo A (2009). The novel Akt inhibitor Palomid 529 (P529) enhances the effect of radiotherapy in prostate cancer. Br J Cancer.

[346] Mabuchi S, Ohmichi M, Kimura A, Hisamoto K, Hayakawa J, Nishio Y, Adachi K, Takahashi K, Arimoto-Ishida E, Nakatsuji Y, Tasaka K, Murata Y (2002). Inhibition of phosphorylation of BAD and Raf-1 by Akt sensitizes human ovarian cancer cells to paclitaxel. J Biol Chem.

[347] Brognard J, Dennis PA (2002). Variable apoptotic response of NSCLC cells to inhibition of the MEK/ERK pathway by small molecules or dominant negative mutants. Cell Death Differ.

[348] Aoki K, Ogawa T, Ito Y, Nakashima S (2004). Cisplatin activates survival signals in UM-SCC-23 squamous cell carcinoma and these signal pathways are amplified in cisplatin-resistant squamous cell carcinoma. Oncol Rep.

[349] Rieber M, Rieber MS (2006). Signalling responses linked to betulinic acid-induced apoptosis are antagonized by MEK inhibitor U0126 in adherent or 3D spheroid melanoma irrespective of p53 status. Int J Cancer.

[350] Sieghart W, Fuereder T, Schmid K, Cejka D, Werzowa J, Wrba F, Wang X, Gruber D, Rasoul-Rockenschaub S, Peck-Radosavljevic M, Wacheck V (2007). Mammalian target of rapamycin pathway activity in hepatocellular carcinomas of patients undergoing liver transplantation. Transplantation.

[351] Ribatti D, Nico B, Mangieri D, Longo V, Sansonno D, Vacca A, Dammacco F (2007). In vivo inhibition of human hepatocellular carcinoma related angiogenesis by vinblastine and rapamycin. Histol Histopathol.

[352] Llovet JM, Ricci S, Mazzaferro V, Hilgard P, Gane E, Blanc JF, de Oliveira AC, Santoro A, Raoul JL, Forner A, Schwartz M, Porta C, Zeuzem S, Bolondi L, Greten TF, Galle PR, SHARP Investigators Study Group (2008). Sorafenib in advanced hepatocellular carcinoma. N Engl J Med.

[353] Choi S, Gamper AM, White JS, Bakkenist CJ (2010). Inhibition of ATM kinase activity does not phenocopy ATM protein disruption: implications for the clinical utility of ATM kinase inhibitors. Cell Cycle.

[354] Kari V, Shchebet A, Neumann H, Johnsen SA (2011). The H2B ubiquitin ligase RNF40 cooperates with SUPT16H to induce dynamic changes in chromatin structure during DNA double-strand break repair. Cell Cycle.

[355] Puca R, Nardinocchi L, Porru M, Simon AJ, Rechavi G, Leonetti C, Givol D, D'Orazi G (2011). Restoring p53 active conformation by zinc increases the response of mutant p53 tumor cells to anticancer drugs. Cell Cycle.

[356] Zhao CY, Grinkevich VV, Nikulenkov F, Bao W, Selivanova G (2010). Rescue of the apoptotic-inducing function of mutant p53 by small molecule RITA. Cell Cycle.

[357] van Vuurden DG, Hulleman E, Meijer OL, Wedekind LE, Kool M, Witt H, Vandertop PW, Wurdinger T, Noske DP, Kaspers GJ, Cloos J (2011). PARP inhibition sensitizes childhood high grade glioma, medulloblastoma and ependymoma to radiation. Oncotarget.

[358] Chung EJ, Brown AP, Asano H, Mandler M, Burgan WE, Carter D, Camphausen K, Citrin D (2009). In vitro and in vivo radiosensitization with AZD6244 (ARRY-142886), an inhibitor of mitogen-activated protein kinase/extracellular signal-regulated kinases 1 /2 kinase. Clin Cancer Res.

[359] Hoglund A, Stromvall K, Li Y, Forshell LP, Nilsson JA (2011). Chk2 deficiency in Myc overexpressing lymphoma cells elicits a synergistic lethal response in combination with PARP inhibition. Cell Cycle.

[360] Carrassa L, Damia G (2011). Unleashing Chk1 in cancer therapy. Cell Cycle.

[361] Noguchi E (2011). Division of labor of the replication fork protection complex subunits in sister chromatid cohesion and Chk1 activation. Cell Cycle.

[362] Kuntziger T, Landsverk HB, Collas P, Syljuasen RG (2011). Protein phosphatase 1 regulators in DNA damage signaling. Cell Cycle.

[363] Lee HJ, Hwang HI, Jang YJ (2010). Mitotic DNA damage response: Polo-like kinase-1 is dephosphorylated through ATM-Chk1 pathway. Cell Cycle.

[364] Smith-Roe SL, Patel SS, Simpson DA, Zhou YC, Rao S, Ibrahim JG, Kaiser-Rogers KA, Cordeiro-Stone M, Kaufmann WK (2011). Timeless functions independently of the Tim-Tipin complex to promote sister chromatid cohesion in normal human fibroblasts. Cell Cycle.

[365] Peddibhotla S, Wei Z, Papineni R, Lam MH, Rosen JM, Zhang P (2011). The DNA damage effector Chk1 kinase regulates Cdc14B nucleolar shuttling during cell cycle progression. Cell Cycle.

[366] Chen Y, Chen CF, Riley DJ, Chen PL (2011). Nek1 kinase functions in DNA damage response and checkpoint control through a pathway independent of ATM and ATR. Cell Cycle.

[367] Piao S, Lee SJ, Xu Y, Gwak J, Oh S, Park BJ, Ha NC (2011). CK1epsilon targets Cdc25A for ubiquitin-mediated proteolysis under normal conditions and in response to checkpoint activation. Cell Cycle.

[368] Bower JJ, Zhou Y, Zhou T, Simpson DA, Arlander SJ, Paules RS, Cordeiro-Stone M, Kaufmann WK (2010). Revised genetic requirements for the decatenation G2 checkpoint: the role of ATM. Cell Cycle.

[369] Wawrousek KE, Fortini BK, Polaczek P, Chen L, Liu Q, Dunphy WG, Campbell JL (2010). Xenopus DNA2 is a helicase/nuclease that is found in complexes with replication proteins And-1/Ctf4 and Mcm10 and DSB response proteins Nbs1 and ATM. Cell Cycle.

[370] Dai Y, Grant S (2010). Targeting Chk1 in the replicative stress response. Cell Cycle.

[371] Golan A, Pick E, Tsvetkov L, Nadler Y, Kluger H, Stern DF (2010). Centrosomal Chk2 in DNA damage responses and cell cycle progression. Cell Cycle.

[372] Pires IM, Bencokova Z, McGurk C, Hammond EM (2010). Exposure to acute hypoxia induces a transient DNA damage response which includes Chk1 and TLK1. Cell Cycle.

[373] Kolupaeva V, Basilico C (2010). FGF inhibits the activity of the cyclin B1/CDK1 kinase to induce a transient G2arrest in RCS chondrocytes. Cell Cycle.

[374] Yu X, Wang H, Liu S, Zhang X, Guida P, Hu B, Wang Y (2010). A small peptide mimicking the key domain of MEPE/OF45 interacting with CHK1 protects human cells from radiation-induced killing. Cell Cycle.

[375] Peart MJ, Poyurovsky MV, Kass EM, Urist M, Verschuren EW, Summers MK, Jackson PK, Prives C (2010). APC/C(Cdc20) targets E2F1 for degradation in prometaphase. Cell Cycle.

[376] Jamil S, Stoica C, Hackett TL, Duronio V (2010). MCL-1 localizes to sites of DNA damage and regulates DNA damage response. Cell Cycle.

[377] Wood MD, Sanchez Y (2010). Deregulated Ras signaling compromises DNA damage checkpoint recovery in S. cerevisiae. Cell Cycle.

[378] McNeely S, Conti C, Sheikh T, Patel H, Zabludoff S, Pommier Y, Schwartz G, Tse A (2010). Chk1 inhibition after replicative stress activates a double strand break response mediated by ATM and DNA-dependent protein kinase. Cell Cycle.

[379] Sorensen CS, Melixetian M, Klein DK, Helin K (2010). NEK11: linking CHK1 and CDC25A in DNA damage checkpoint signaling. Cell Cycle.

[380] Sierant ML, Archer NE, Davey SK (2010). The Rad9A checkpoint protein is required for nuclear localization of the claspin adaptor protein. Cell Cycle.

[381] Varmark H, Kwak S, Theurkauf WE (2010). A role for Chk2 in DNA damage induced mitotic delays in human colorectal cancer cells. Cell Cycle.

[382] Merry C, Fu K, Wang J, Yeh IJ, Zhang Y (2010). Targeting the checkpoint kinase Chk1 in cancer therapy. Cell Cycle.

[383] Quiros S, Roos WP, Kaina B (2010). Processing of O6-methylguanine into DNA double-strand breaks requires two rounds of replication whereas apoptosis is also induced in subsequent cell cycles. Cell Cycle.

[384] Edwards E, Geng L, Tan J, Onishko H, Donnelly E, Hallahan DE (2002). Phosphatidylinositol 3-kinase/Akt signaling in the response to vascular endothelium to ionizing radiation. Cancer Res.

[385] Shinohara ET, Cao C, Niermann K, Mu Y, Zeng F, Hallahan DE, Lu B (2005). Enhanced radiation damage of tumor vasculature by mTOR inhibitors. Oncogene.

[386] Paglin S, Lee NY, Nakar C, Fitzgerald M, Plotkin J, Deuel B, Hackett N, McMahill M, Sphicas E, Lampen N, Yahalom J (2005). Rapamycin-sensitive pathway regulates mitochondrial membrane potential, autophagy, and survival in irradiated MCF-7 cells. Cancer Res.

[387] Moretti L, Attia A, Kim KW, Lu B (2007). Crosstalk between Bak/Bax and mTOR signaling regulates radiation induced autophagy. Autophagy.

[388] Wu JJ, Quijano C, Wang J, Finkel T (2010). Metabolism meets autophagy. Cell Cycle.

[389] Pani G (2011). From growing to secreting: new roles for mTOR in aging cells. Cell Cycle.

[390] Korkaya H, Wicha MS (2011). Inflammation and autophagy conspire to promote tumor growth. Cell Cycle.

[391] Boehrer S, Lainey E, Kroemer G (2011). Coordinated epigenetic regulation of autophagy and apoptosis. Cell Cycle.

[392] Rambold AS, Lippincott-Schwartz J (2010). Starved cells use mitochondria for autophagosome biogenesis. Cell Cycle.

[393] Galluzzi L, Morselli E, Kepp O, Maiuri MC, Kroemer G (2010). Defective autophagy control by the p53 rheostat in cancer. Cell Cycle.

[394] Puissant A, Robert G, Auberger P (2010). Targeting autophagy to fight hematopoietic malignancies. Cell Cycle.

[395] Cluzeau T, Robert G, Puissant A, Jean-Michel K, Cassuto JP, Raynaud S, Auberger P (2011). Azacitidine-resistant SKM1 myeloid cells are defective for AZA-induced mitochondrial apoptosis and autophagy. Cell Cycle.

[396] Watson AS, Mortensen M, Simon AK (2011). Autophagy in the pathogenesis of myelodysplastic syndrome and acute myeloid leukemia. Cell Cycle.

[397] Martinez-Outschoorn UE, Whitaker-Menezes D, Pavlides S, Chiavarina B, Bonuccelli G, Casey T, Tsirigos A, Migneco G, Witkiewicz A, Balliet R, Mercier I, Wang C, Flomenberg N, Howell A, Lin Z, Caro J (2010). The autophagic tumor stroma model of cancer or “battery-operated tumor growth”: A simple solution to the autophagy paradox. Cell Cycle.

[398] Castello-Cros R, Bonuccelli G, Molchansky A, Capozza F, Witkiewicz AK, Birbe RC, Howell A, Pestell RG, Whitaker-Menezes D, Sotgia F, Lisanti MP (2011). Matrix remodeling stimulates stromal autophagy, “fueling” cancer cell mitochondrial metabolism and metastasis. Cell Cycle.

[399] Iozzo RV (2011). Autophagic tumor stroma: a biofuel for cancer growth. Cell Cycle.

[400] Zhou J, Wulkuhle J, Zhang H, Gu P, Yang Y, Deng J, Margolick JB, Liotta LA, Petricoin E, Zhang Y (2007). Activation of the PTEN/mTOR/STAT3 pathway in breast cancer stem-like cells is required for viability and maintenance. Proc Natl Acad Sci USA.

[401] Chapuis N, Tamburini J, Green AS, Willems L, Bardet V, Park S, Lacombe C, Mayeux P, Bouscary D (2010). Perspectives on inhibiting mTOR as a future treatment strategy for hematological malignancies. Leukemia.

[402] Martelli AM, Evangelisti C, Follo MY, Ramazzotti G, Fini M, Giardino R, Manzoli L, McCubrey JA, Cocco L (2011). Targeting the phosphatidylinositol 3-kinase/Akt/mammalian target of rapamycin signaling network in cancer stem cells. Current Medicinal Chemistry.

[403] McCubrey JA, Steelman LS, Abrams SL, Misaghian N, Chappell WH, Bäsecke J, Nicoletti F, Libra M, Ligresti G, Stivala F, Maksimovic-Ivanic D, Mijatovic S, Montalto G, Cervello M, Laidler P, Bonati A (2011). Targeting the cancer initiating Cell: The ultimate target for cancer therapy. Current Pharmaceutical Design.

[404] Song MS, Salmena L, Pandolfi PP (2012). The functions and regulation of the PTEN tumour suppressor. Nature Reviews Molecular Cell Biology.

[405] Bednar F, Simeone DM (2012). Metformin and cancer stem cells: old drug, new targets. Cancer Prev Res.

[406] Bao B, Wang Z, Ali S, Ahmad A, Azmi AS, Sarkar SH, Banerjee S, Kong D, Li Y, Thakur S, Sarkar FH (2012). Metformin inhibits cell proliferation, migration and invasion by attenuating CSC function mediated by deregulating miRNAs in pancreatic cancer cells. Cancer Prev Res (Phila).

